# Connexin 43 is downregulated in advanced Parkinson’s disease in multiple brain regions which correlates with symptoms

**DOI:** 10.1038/s41598-025-94188-7

**Published:** 2025-03-25

**Authors:** Nataly Hastings, Saifur Rahman, Przemyslaw Aleksander Stempor, Matthew T. Wayland, Wei-Li Kuan, Mark R. N. Kotter

**Affiliations:** 1 Department of Clinical Neurosciences, University of Cambridge, Cambridge, CB2 0QQ UK; 2 Wellcome-MRC Cambridge Stem Cell Institute, University of Cambridge, Cambridge, CB2 0AW UK; 3 Electrical Engineering Division, Department of Engineering, University of Cambridge, Cambridge, CB3 0FA UK; 4 Wellcome Trust / Cancer Research UK Gurdon Institute, University of Cambridge, Cambridge, CB2 1QN UK; 5 Department of Zoology, University of Cambridge, Cambridge, CB2 3EJ UK; 6 Alborada Drug Discovery Institute, University of Cambridge, Cambridge, CB2 0AH UK

**Keywords:** Astrocyte, Gap junction, Connexin43, Parkinson’s disease, Microscopy, Mechanisms of disease, Cellular neuroscience, Glial biology, Neural ageing, Parkinson's disease, Imaging

## Abstract

Parkinson’s disease (PD) is a neurodegenerative condition with the greatest increase in disability globally. Dysfunction of dopaminergic neurons is a well-known PD hallmark; however, changes in astrocytes also accompany PD progression. One aspect of astrocyte biology not yet investigated in PD is their network coupling. To assess this, we focussed on the major astrocytic gap junctional protein connexin 43 (Cx43, GJA1). A dataset of 20 post-mortem late-stage PD brain tissue samples from the cortex and basal ganglia alongside 20 age-matched control sets was collected, accompanied by clinical histories and data on α-synuclein, tau, and amyloid-β pathology. Protein levels and intracellular distribution of Cx43 and other key markers were measured. Computational re-analysis of open-source mRNA sequencing datasets from the striatum and midbrain complemented the original findings. Two novel observations were made: first, profound Cx43 loss in late-stage PD, and second, differential manifestation of this pathology in different brain areas, including those outside of the midbrain substantia nigra—the region that is most commonly used in PD research. Cx43 downregulation in specific regions correlated with non-motor symptoms of PD such as depression and sleep disturbance. Astrocytic tree simplification in the frontal cortex was further observed. In conclusion, astrocytic network decoupling through Cx43 downregulation in PD may contribute to astrocytic dysfunction and PD symptom development.

## Introduction

Parkinson’s disease (PD) typically affects older individuals over 65 years of age, with approximately 10 million people living with PD globally. With the highest increase in prevalence among neurodegenerative disorders that is linked to the population ageing^1^, PD diagnoses in the UK are predicted to rise by 1/5th by 2025^2^. Currently available treatments focus on replacing dopamine in the brain since dopaminergic neurons of the midbrain substantia nigra (SN) suffer and die in PD, resulting in decreased dopaminergic innervation of the striatum and ultimately the characteristic motor symptoms such as tremor, rigidity, bradykinesia, and postural instability. The cause of this pathology is not known, and dopamine replacement does not stop PD from worsening over time; furthermore, serious side effects including hallucinations and dyskinesias can limit its benefits^3^. In addition, non-motor symptoms (NMS) which are not directly associated with dopamine deficits and include dementia, depression, speech disturbance, slower gastrointestinal transit, and sleep problems, are not specifically addressed; these symptoms substantially affect quality of life of at least half of the people with PD^4^. Neuroprotective strategies that have been successful in toxin models of acute dopaminergic denervation thus far have largely not translated into the clinic, suggesting that additional mechanisms may trigger PD progression in humans. Thus, there is a large unmet clinical need to understand the core causes of PD pathology to devise innovative treatment strategies that do not only mask the symptoms but also modify the disease progression by tapping into the core PD pathology.

The involvement of other cell types outside of the nigrostriatal system has been previously suggested and explored. For instance, cholinergic deficits have been described^5^, and immune cell infiltration into the brain has been proposed as a driver of PD^6^. Astrocytes represent another brain cell type that has recently gained prominence as a key regulator of cognitive^7,8^ and motor^9^ functions, and dysfunction of these cells has been reported in human PD and models. For example, astrocytes express almost all familial PD-associated genes as much, or more than neurons do^10^—this includes α-synuclein (a-syn), the astrocytic expression of which may be enhanced by PD-associated mutations^11,12^. Abnormal astrocytic activation has been shown in human PD^13,14^ and models^15^, and astrocytic a-syn inclusions have been described in human PD by Braak^16^ and others; their presence in the midbrain SN parallels dopaminergic neuron loss^17^. Causal involvement of this cell type in PD symptom development has been demonstrated in vitro and in vivo, where healthy astrocytes can rescue the functions of neurons carrying PD mutations^11^ and reduce motor symptoms in rodent models^18,19^, and diseased astrocytes can induce PD-resembling dysfunction in healthy neurons^11^ or animals^20,21^. For example, the overexpression of a-syn in astrocytes, not in neurons, was sufficient to generate severe motor dysfunction in rodents^20^, whereas the transplantation of healthy astrocytes ameliorated motor deficits in a model of PD generated by 6-hydroxydopamine (6-OHDA) lesions^18^.

The ability to form large tessellated networks, the syncytium, is an integral part of astrocyte biology; these networks have two main modes of signal transmission—chemical transmitter release followed by the engagement of membrane-bound receptors akin to neuronal synaptic communication, and direct cytoplasmic linkage via proteins called connexins^22^. While other brain cells also express connexins, gap junctional (GJ) coupling is particularly prominent in astrocytes—GJs are structures formed by connexins that link opposing cell membranes and allow for the passage of second messengers and metabolites up to 1 kDa such as inositol triphosphate and glucose, as well as electrical coupling^23^. These networks might contribute to long-range information transfer in the brain in parallel with neuronal networks^24^.

Connexin43 (Cx43, encoded by the GJA1 gene) is the major member of the connexin protein family expressed predominantly by astrocyte-lineage cells within the nervous system^25^. Previous research has suggested that Cx43 pathology might contribute to PD progression but the reports on the *direction of change* in Cx43 function in PD have been inconsistent. The upregulation of astrocytic Cx43 and phospho-Cx43 has been reported in rotenone models of PD in rat models in vivo and *in vitro*^26,27^; an increase in GJA1 mRNA and Cx43 immunoreactivity has been shown in a mouse 1-methyl-4-phenyl-1,2,3,6-tetrahydropyridine (MPTP) model^28^, and increased Cx43 expression has been observed in a lipopolysaccharide (LPS)-induced rat model of PD^29^. In contrast, in another study, rotenone was found to downregulate Cx43 expression and GJ coupling of rat astrocytes *in vitro*^30^, and impaired GJ coupling was found in a-syn-challenged mouse astrocytes in culture^31^. The therapeutic effects of functional Cx43 modulation have not yet been fully established. While enhancing GJ opening prevented rat astrocyte apoptosis in a rotenone-induced model *in vitro*^30^, blocking Cx43 with GAP27 peptide had neuroprotective effects in mouse models induced by 6-OHDA^32^ or LPS^33^.

We hypothesised that the following factors could have resulted in the discrepancies:

1. PD model used—many in vitro and in vivo models of PD focus on dopaminergic toxicity and do not recapitulate the underlying core pathology behind PD that leads to dopaminergic loss among other symptoms; these include 6-OHDA, MPTP, and rotenone. Whilst their dopaminergic toxicity is well-documented, their ability to recreate the astrocytic pathology has not been confirmed.
2. The brain region studied—astrocytes represent a diverse cell type, both between^34^ and within^35,36^ different brain regions; just like GABAergic neurons respond differently to dopaminergic neurons in PD, so could unique regional astrocyte subtypes.
3. mRNA vs protein levels vs function—the Cx43 pathway is complex, where no direct equivalence can be made between the levels of mRNA, protein, and functional channel opening. For example, pharmacological blockade of Cx43 channels might paradoxically increase mRNA levels^37^. Moreover, functionally Cx43 can exist in two main conformational states regulated by post-translational modifications—GJs linking opposing cells in networks, and unopposed hemichannels (HCs) open to the extracellular milieu. While the consensus on the latter is that HCs represent the largely “pathological” form that becomes upregulated in disease including a-syn and MPTP models^31,38^, the roles of the former lack consensus in the context of PD.

To address some of the existing inconsistencies in the literature on Cx43 in PD, we set out to study the distribution patterns of this protein in the human post-mortem brain tissue as opposed to animal models of PD; in this way, we could ensure that the underlying core PD pathology that is yet to be fully defined and modelled is captured in our dataset as opposed to dopaminergic denervation only. We also sampled a greater range of brain regions including several cortical areas (parietal, frontal, and insular) and multiple parts of the basal ganglia system (striatum, globus pallidus, caudate, and midbrain SN) to obtain a broader overview of the Cx43 pathology distribution. The results were corroborated through the use of complimentary protein assessment techniques—immunostaining and western blotting. Multiple correlations between Cx43 pathology and other astrocytic markers, microglial marker (Iba1) expression in the cortex, as well as NMS, Braak stages (for a-syn, amyloid, and tau), cerebral amyloid angiopathy (CAA), and SN depigmentation were explored, resulting in a rich dataset that is summarised here and can be accessed in full via our user-friendly interactive application (https://www.cx43pd.com). Existing single cell sequencing datasets from the human midbrain and striatum were re-analysed by us to compare our observations of Cx43 protein levels with the GJA1 mRNA expression changes in PD. Finally, astrocytic morphology in the frontal cortex was evaluated in PD and control cases.

Overall, this work provides a comprehensive initial overview of Cx43 protein pathology in human PD, providing justification for further functional exploration of this protein as a possible therapeutic target.

## Results

### Cx43 gap junctional and total staining is decreased in PD in a brain region-specific pattern

The Cx43 pathway is composed of two main functional components—GJs and HCs, which present with differential subcellular distributions. While HCs are smaller structures comprising individual channels composed of Cx43 hexamers on the cell surface, GJs often accumulate in large (up to several μm) dynamic structures called junctional plaques, which reside on the cell membrane and interact with the cytoskeleton^39,40^. These plaques tend to give rise to distinct punctate staining as opposed to the diffuse membrane staining, which is more likely to be indicative of HCs, or intracellular staining which can represent internalised Cx43 or the protein en route to the membrane from the Golgi complex.

An immunofluorescence staining of FFPE human brain slices from seven brain areas (frontal cortex, insular cortex, parietal cortex, striatum, globus pallidus, caudate, and midbrain SN; see Methods for patient demographics) was performed; this staining of 40 cases in total was split into 6 staining batches, each containing equal numbers of control and PD cases. Fluorescence images were acquired in the photon counting mode on a Leica Stellaris 8 microscope to make the imaging analysis maximally comparable between the batches, and statistical batch effect correction was introduced during the analysis.

**Fig. 1:**
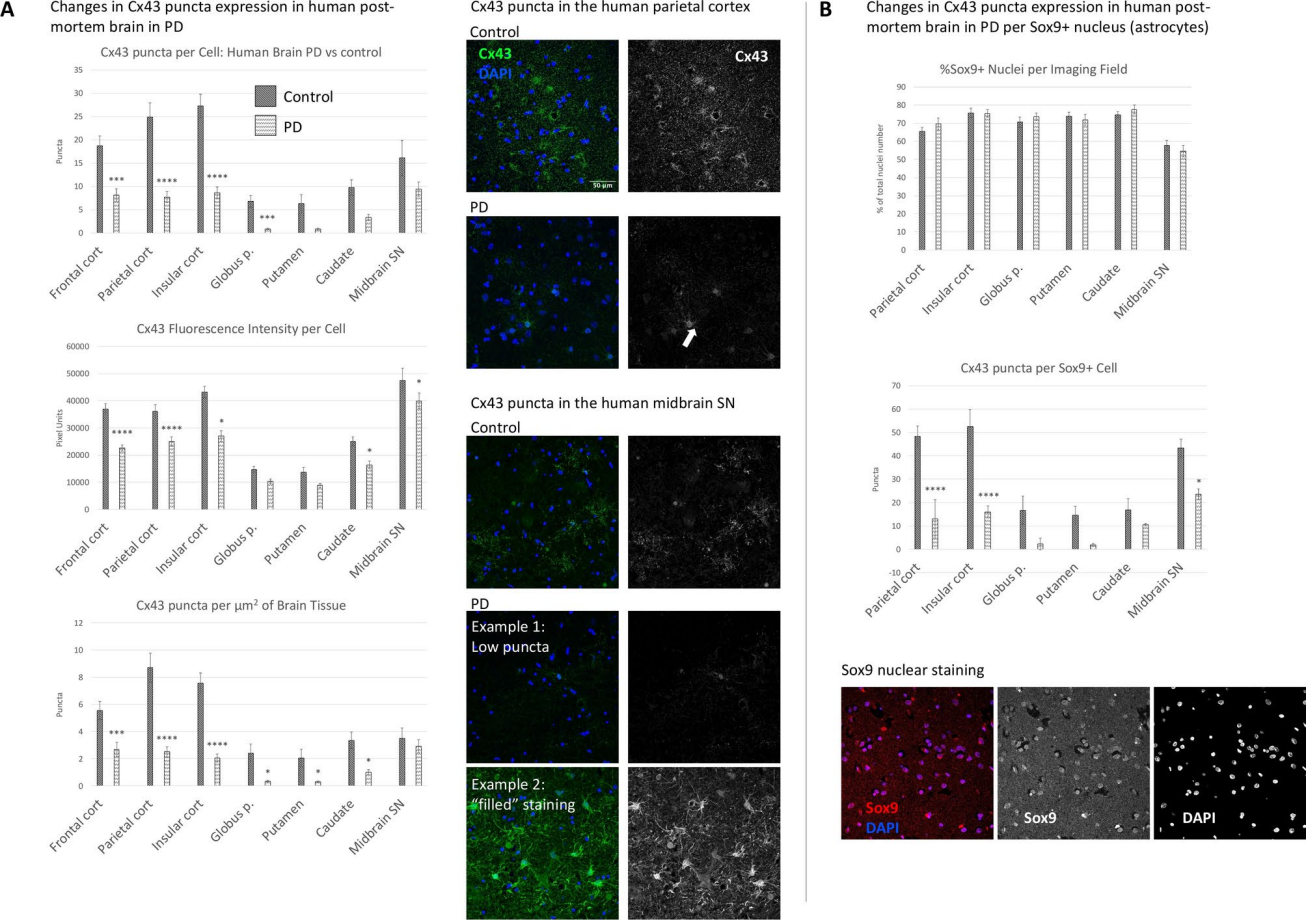
Changes in Cx43 puncta between PD patients and controls within selected brain regions. (**A**) Cx43 puncta and total fluorescence intensity were estimated and normalised per nucleus, or per μm^2^ of brain tissue. Profound Cx43 downregulation has been observed in PD, especially across the cerebral cortex. Midbrain SN Cx43 staining presented with an inconsistent pattern, often not corresponding to the punctate staining indicative of the GJ and presenting with a “filled” appearance where cell bodies and major processes are largely uniformly stained with Cx43. The arrow indicates an example of an astrocytic process filled with diffuse Cx43 staining. n = 20 per group, at least 3 microscopic images per sample. Note that for some individuals, not all sub-regional samples were available, making the total n for select regions as follows: Parietal Cort Control n = 19; Insular Cort Control n = 18; Putamen Control n = 19; Globus p. Control n = 16; Globus p. PD n = 15; Caudate Control n = 15; Caudate PD n = 14. (**B**) The proportion of astrocytes among the sampled cells was estimated via nuclear Sox9 staining. Compared with other brain regions, the midbrain SN presented with a lower average proportion of Sox9 + cells (astrocyte lineage), and Cx43 downregulation per astrocyte-lineage cell in PD was seen in the midbrain and cerebral cortex. n = 10 per group. Statistical analysis for all graphs: ANOVA with FDR correction; error bars: SEM; “Control” vs “PD” values are compared *within* each brain region. *p < 0.05, **p < 0.005, ***p < 0.0005, ****p < 0.0001. PD—Parkinson’s disease; Cx43—connexin 43; Frontal cort—frontal cortex; Parietal cort—parietal cortex; Insular cort—insular cortex; Globus p.—globus pallidus; Midbrain SN—midbrain substantia nigra.

The images demonstrated a clear punctate Cx43 distribution that was particularly evident in the cortical control samples, whereas midbrain SN samples often presented with a non-punctate “filled” cellular staining (Fig. 1A), which was more common in PD than in controls (55% vs 23%, respectively). Negative staining controls containing secondary antibodies only were introduced to ensure that the punctate pattern was not due to imaging artefacts and to estimate the non-specific background (data not shown). These Cx43 puncta (assumed to largely represent GJ plaques) were analysed via the “3D Object Counter” function in Fiji alongside the total Cx43 fluorescence intensity, which is likely to represent the total Cx43 protein level. No cells or images, including those with the “filled” Cx43 morphology, were excluded from the automated Cx43 puncta analysis.

**Fig. 2:**
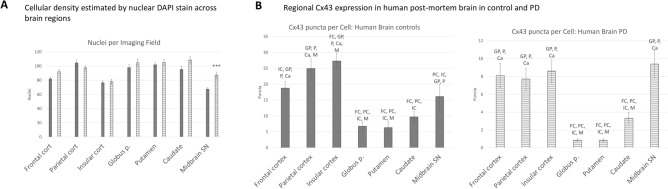
Cellular density within selected brain regions and the distribution of Cx43 puncta across brain regions. (**A**) Cellular density was estimated by counting the number of DAPI-stained nuclei per imaging field. Only the midbrain SN presented a different cellular density in PD vs control samples, with a higher density of cells in PD. n = 20 per group, at least 3 microscopic images per sample. ***p < 0.0005 (*p* = 0.0004). (**B**) Cx43 puncta expression was compared *between* selected brain regions, independently in control and PD brains. n = 20 per group, at least 3 microscopic images per sample. Note that for some individuals, not all sub-regional samples were available, making the total n for select regions as follows: Parietal Cort Control n = 19; Insular Cort Control n = 18; Putamen Control n = 19; Globus p. Control n = 16; Globus p. PD n = 15; Caudate Control n = 15; Caudate PD n = 14. Statistical analysis for all graphs: ANOVA with FDR correction; error bars: SEM. Statistical significance (at least *p* < 0.05) is shown for each region compared with all other regions; significance is shown for each region relative to the following: FC—Frontal cortex, PC—Parietal cortex, IC—Insular cortex, GP—Globus p., P—Putamen, Ca—Caudate, M—Midbrain SN. In control cases, there was a significant difference between parietal and insular cortical samples and samples from the midbrain SN, but this difference disappeared in PD. PD—Parkinson’s disease; Cx43—connexin 43; Globus p.—globus pallidus; Midbrain SN—midbrain substantia Nigra.

Cx43 puncta and total fluorescence (capturing total Cx43 staining) normalised to the number of DAPI-labelled cell nuclei demonstrated a profound downregulation of Cx43 in the cortical regions of PD patients (Fig. 1A). The basal ganglia also presented with a PD-associated Cx43 decrease, but only the globus pallidus presented significant Cx43 puncta loss, while the caudate and midbrain SN exhibited significant decreases in Cx43 fluorescence intensity per cell. Cx43 puncta were also estimated per μm^2^ of brain tissue, where all regions except the midbrain SN presented with a significant decrease in PD (Fig. 1A). To evaluate which proportion of the cells in each sampled region were likely to be astrocytes, Sox9 nuclear staining^41^ was employed on a subset of samples from 10 PD and 10 control cases. No significant differences in the Sox9 + to Sox9- nuclei ratios were observed between PD and controls within each region, with a curious trend toward a lower overall proportion of Sox9 + cells in the midbrain SN (55–60% in the midbrain SN vs 70–80% in other regions, Fig. 1B). Cx43 puncta were significantly downregulated per Sox9 + nucleus (Fig. 1B) in PD in the cortical regions and midbrain SN. Sox9 + cells represented over half of nuclei in most brain regions, which contrasts with previously published observations of only 10–20% of total cells being Sox9 + ^41^, inviting a cautious interpretation. Interestingly, midbrain SN samples in our dataset presented a significantly greater overall cell density in PD, suggesting a possible immune cell infiltration, microglial migration or proliferation, and a possible compensatory proliferation of astrocytic lineage subsets (Fig. 2A).

Inter-regional Cx43 puncta distribution patterns were examined separately in the control and PD datasets, which revealed a greater density of Cx43 puncta per cell in the cortical areas and significantly sparser Cx43 puncta in the globus pallidus, putamen, and caudate (Fig. 2B). The midbrain SN presented a significantly lower Cx43 puncta density per cell than did the parietal and insular cortical samples in controls, but this difference disappeared in PD (Fig. 2B).

The full imaging dataset including raw files and snapshots is accessible via https://www.ebi.ac.uk/biostudies/bioimages/studies/S-BIAD1133 (10.6019/S-BIAD1133).

## Cx43 protein levels are reduced in PD in a brain region-specific pattern

To complement the fluorescence staining of GJ puncta, we measured Cx43 protein levels via Western blotting; the Triton X-100-soluble fraction was captured, which could represent non-junctional Cx43^42^, although intracellular non-junctional Cx43 puncta may also present with Triton X-100 insolubility^43^. Thus, this method allowed us to estimate the levels of the “non-punctate” Cx43 potentially not well-captured in the microscopic analysis (due to the possible contribution of low levels of residual background autofluorescence), which might represent the intracellular protein en route to its destination^42^, and thus the ability of cells to produce Cx43. Densitometry analysis of the ECL-visualised bands followed by the normalisation to the total protein levels was performed, and all densitometry measurements of the bands on a selected membrane were normalised to an Expression Control sample (from a control parietal cortex) which was loaded at the same protein concentration across all the experiments to allow for a semi-quantitative comparison of the bands ran on separate membranes.

**Fig. 3:**
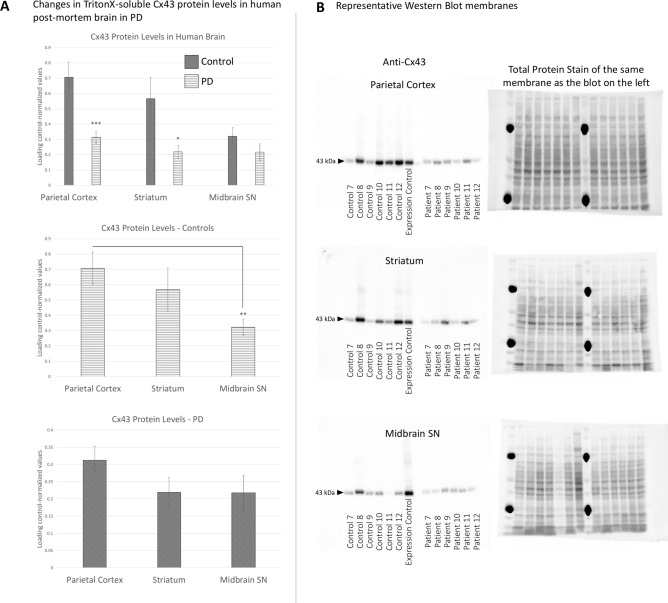
Changes in Triton X-100-soluble Cx43 levels between PD patients and controls within selected brain regions. (**A**) Densitometry values of the Cx43 bands were normalised to the total protein far red (700 nm) fluorescence staining, and then further to the Expression Control sample from a control parietal cortex (representing a relative value of 1) which was run on each membrane among the experimental samples. Relative values were then semi-quantitatively compared within selected regions between PD and controls, or between selected regions in each condition separately. Significant Cx43 downregulation was found in PD in the parietal cortex and striatum. In healthy individuals, the parietal cortex expressed significantly higher Cx43 than the midbrain SN, but this difference was diminished in PD. n = 20 per group, except Control Midbrain SN samples, where total n = 18. Statistical analysis for all graphs: ANOVA with FDR correction; error bars: SEM; “Control” vs “PD” values are compared *within* each brain region. *p < 0.05, **p < 0.005, ***p < 0.0005. (**B**) Representative Western blot membranes were stained against total protein (visualised at 700 nm) and counterstained with anti-Cx43 (visualised via ECL). All Western blot membranes presented are full images as obtained; no editing, cropping, or other image manipulation was introduced (other than the digital inverting for “dark bands on light background” conventional Western blot representation purposes). Exposure times were selected so that none of the sample bands were overexposed, which was assessed with the Image Studio software (Li-Cor). Full Western blot dataset is provided in the Supplementary materials. PD—Parkinson’s disease; Cx43—connexin 43; Midbrain SN—midbrain substantia nigra.

We detected a significant decrease in the level of the Triton X-100-soluble Cx43 in the parietal cortex and striatal samples, whereas the trend toward lower Cx43 in the midbrain SN did not reach significance (Fig. 3). In the control cases, there was a significant difference between the cortical and midbrain SN Cx43 protein levels, which was lost in PD (Fig. 3A).

Notably, the pattern of the Triton X-100-soluble Cx43 protein loss in PD overall followed the pattern observed via the microscopic GJ analysis. Striatal Cx43, however, was greater in the Western blot assay (relative to other regions) than in the microscopic images. This may suggest that different proportions of total Cx43 may be sequestered in the insoluble fraction depending on the brain region.

## Correlation analysis of Cx43 and other molecular hallmarks of PD

Next, it was investigated whether Cx43 downregulation that is associated with PD exhibits correlation trends with known molecular hallmarks of the disease such as aggregated protein inclusions and SN depigmentation, as well as our Western blot protein analysis of other prominent astrocytic markers in addition to a microglial marker. FDR correction of the p-value was performed and is presented in the correlation tables; however, correction may not be representative in small exploratory datasets such as the one discussed here^44^ hence raw p-values and Spearman’s rank order correlation coefficient (rho) were also taken into consideration. The effect size (rho) is the key statistic in an exploratory analysis such as the one presented here (where many tests are performed on a relatively small sample) because it outlines the strength of the relationship between the two variables. The full dataset and various correlations can be explored further in depth using an interactive web application: (https://www.cx43pd.com).

**Fig. 4:**
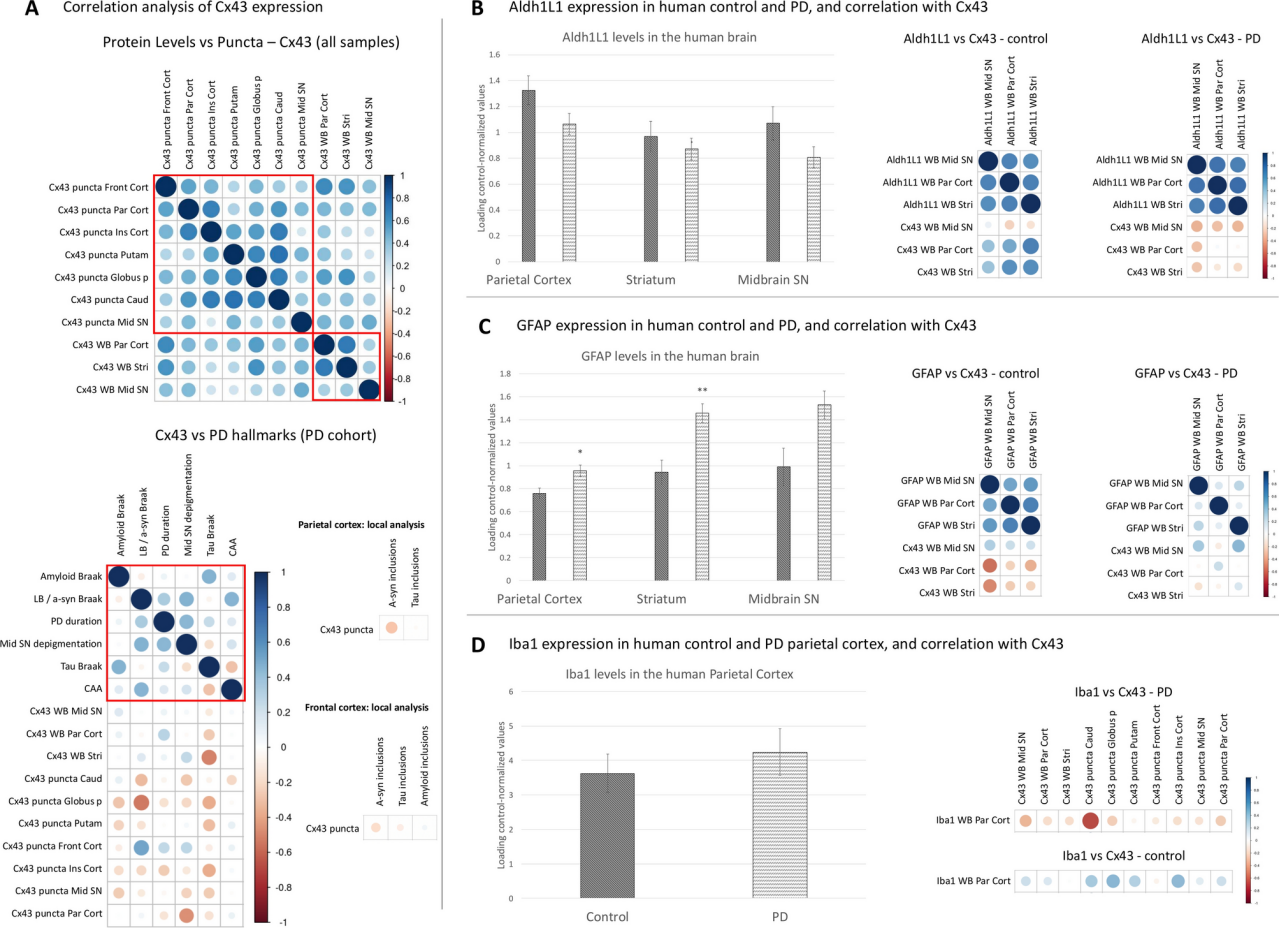
Correlations between Cx43 expression and various PD hallmarks or other cellular markers. (**A**) Correlation analysis of Cx43 expression within individuals across all samples (n = 40), and between Cx43 expression and hallmarks of PD pathology (n = 20, PD cohort only). The heatmap scale represents the value of the Spearman’s correlation coefficient, with blue colour indicating a positive correlation and red—negative correlation (or anti-correlation); the respective correlation coefficients and p-values are shown in Supplementary Tables 3 and 4. The red squares represent related grouped variables for the ease of visual reference. (**B**) Expression and correlation (with Cx43) analysis of the Aldh1L1 astrocytic marker. (**C**) Expression and correlation (with Cx43) analysis of the GFAP astrocytic marker. (**D**) Expression and correlation (with Cx43) analysis of the Iba1 microglial marker. For (**B–D**) Protein expression in control and PD brains was evaluated via Western blot analysis of the Triton X-100-soluble fraction. The respective correlation coefficients and p-values for (**B–D)** are shown in Supplementary Table 5. n = 20 per group (except PD midbrain SN samples, where total n = 18). Representative Western blot membranes for GFAP, Aldh1L1, and Iba1 proteins can be found in the Supplementary materials. Statistical analysis for all graphs: ANOVA with FDR correction; error bars: SEM. *p < 0.05, **p < 0.005. PD—Parkinson’s disease; Cx43—connexin 43; GFAP—glial fibrillary acidic protein; Front Cort—frontal cortex; Par Cort—parietal cortex; Ins Cort—insular cortex; Putam—putamen; Globus p—globus pallidus; Caud—caudate; Mid SN—midbrain substantia nigra; Stri—striatum; LB—Lewy body; a-syn—α-synuclein; CAA—cerebral amyloid angiopathy.

Cx43 levels generally correlated *within* individuals; i.e., higher Cx43 protein levels measured by Western blot were predictive of greater chances of prominent microscopic GJ pattern detection in a single individual (Fig. 4A, Suppl. Table 3). In the PD cohort, the Braak stages of Lewy bodies / a-syn (LB), tau pathology, and amyloid-β pathology, as well as SN depigmentation showed anti-correlation trends with Cx43 expression which was significant in selected regions: the LB Braak stage anti-correlated most strongly with Cx43 puncta in the globus pallidus but correlated positively with Cx43 puncta in the frontal cortex, the tau Braak stage anti-correlated with Cx43 protein expression in the striatum, and SN depigmentation anti-correlated with Cx43 puncta in the parietal cortex. CAA scores did not show a consistent correlation pattern with Cx43 loss in PD (Fig. 4A, Suppl. Table 4). Given that only two LB Braak stages were analysed with almost three times more PD individuals presenting with Braak stage 6 than 5, it is possible that the correlation analysis might not be representative of the relationship between a-syn inclusions and Cx43. To address this, further analysis of the correlation between cortical Cx43 puncta (parietal and frontal cortices) and local a-syn, amyloid, and tau inclusions was performed, revealing mild anti-correlation trends between the aggregated protein load and changes in puncta numbers (Fig. 4A, Suppl. Table 4).

Aldh1L1 is one of the most ubiquitous markers of the astrocytic lineage and was measured in our samples via Western blotting. This marker tended to be downregulated in PD and showed a correlation trend with Cx43 expression in healthy individuals (except for the midbrain SN) but not in PD (Fig. 4B, Suppl. Table 5, Suppl. Fig. 1).

Glial fibrillary acidic protein (GFAP) predominantly labels reactive astrocytes that have undergone astrogliosis, which is typically associated with inflammation, and its upregulation in PD has been described previously^44^. Our current results confirmed and extended those findings as GFAP protein levels were significantly elevated in the parietal cortex and striatum of PD patients (Fig. 4C). GFAP expression, especially in the midbrain SN, was inversely correlated with the Cx43 protein level in the parietal cortex and striatum of healthy individuals, but this pattern was disrupted in PD (Fig. 4C, Suppl. Table 5, Suppl. Fig. 1), possibly because an increased number of astrocytes begin to express this marker under disease conditions.

Overall, it was noted that Cx43 was dysregulated in PD in a pattern that was unique compared to other astrocytic markers like Aldh1L1 and GFAP. Cx43 expression, when adjusted to Aldh1L1 or GFAP, still expressed similar trends towards downregulation in the PD group, arguing against the pan-astrocytic protein decline in PD (Suppl. Fig. 1).

Iba1 is a microglial marker that has been shown to be elevated in PD in the past^44^, thus it was included in the Western blot analysis of the parietal cortex samples since this region presented the greatest degree of Cx43 pathology in PD. Despite a slight trend towards elevated Iba1 in the parietal cortex in PD, this parameter only showed a strong correlation with Cx43 puncta loss in the caudate region in the PD cohort and no anti-correlation with Cx43 expression in healthy individuals (Fig. 4D, Suppl. Table 5, Suppl. Fig. 1). Increased protein levels of Iba1 showed correlation trends with LB / a-syn Braak stage and SN depigmentation (Suppl. Fig. 2A, Suppl. Table 6).

## Correlation analysis of Cx43 loss and clinical features of PD

Clinical histories were examined for all patients (except two PD patients and six control individuals for whom clinical records were not available), and non-motor symptoms were noted; motor and gastrointestinal symptoms were not included in the analysis since they were present in all sampled PD cases. Age at death and duration of PD did not appear to be strong predictors of Cx43 downregulation in the PD cohort (Suppl. Fig. 2B, Suppl. Table 7).

Raw *p*-values, FDR-adjusted *p*-values, and effect sizes estimated by Cohen’s D were considered. Equality of variance was evaluated to choose an appropriate parametric / non-parametric test.

Cx43 puncta loss showed a general correlation trend with a limbic type of PD, except in the frontal cortex, where a decrease in the number of Cx43 puncta was strongly associated with the neocortical pattern (Table 1).

**Table 1:** Correlation between Cx43 and clinical symptoms in PD. Relationships between astrocytic marker expression (with a focus on Cx43) and a number of non-motor symptoms; samples from the PD cohort were included in the analysis.

Independent variable	Dependent variable	Categories	Category n	Equal variances?	Raw *p*-value	Adj. *p*-value (FDR)	Cohens D	Effect size
PD cohort, protein expression (WB) data								
Depression	Par Cort Cx43	0/1	8/10	True	0.034	0.327	1.099	Large
Sleep disturbance		0/1	3/15	True	0.15	0.858	0.957	Large
Aggression		0/1	11/7	True	0.083	0.662	−0.895	Large
Depression	Stri Cx43	0/1	8/10	True	0.023	0.327	1.194	Large
Sleep disturbance		0/1	3/15	True	0.015	0.327	1.714	Large
Sleep disturbance	Mid SN Cx43	0/1	3/15	True	0.148	0.858	0.962	Large
PD cohort—Cx43 puncta (IHC) data								
LB disease type	Front Cort Cx43 puncta	limbic/neocortical	4/15	True	0.158	0.544	−0.832	Large
Depression		0/1	8/10	True	0.032	0.494	1.115	Large
Sleep disturbance		0/1	3/15	True	0.06	0.494	1.28	Large
LB disease type	Par Cort Cx43 puncta	limbic/neocortical	4/15	True	0.133	0.544	0.888	Large
Depression		0/1	8/10	True	0.02	0.494	1.221	Large
LB disease type	Ins Cort Cx43 puncta	limbic/neocortical	4/15	True	0.04	0.494	1.228	Large
Memory problems		0/1	9/9	True	0.054	0.494	0.979	Large
LB disease type	Putamen Cx43 puncta	limbic/neocortical	4/15	True	0.115	0.544	0.935	Large
Psychotic symptoms		0/1	13/5	True	0.061	0.494	1.059	Large
LB disease type	Globus P Cx43 puncta	limbic/neocortical	3/11	True	0.136	0.544	1.042	Large
Aggression		0/1	9/5	True	0.155	0.544	−0.846	Large
LB disease type	Caudate Cx43 puncta	limbic/neocortical	2/12	True	0.111	0.544	1.314	Large
Dementia (late-stage)		0/1	3/9	True	0.169	0.544	0.989	Large
Depression		0/1	5/7	True	0.078	0.539	1.148	Large
Memory problems		0/1	6/6	True	0.182	0.544	0.829	Large
Hallucinations		0/1	3/9	True	0.169	0.544	0.989	Large
Psychotic symptoms		0/1	9/3	True	0.063	0.494	1.395	Large
LB disease type	Mid SN Cx43 puncta	limbic/neocortical	4/15	True	0.097	0.544	0.99	Large

Only noteworthy differences are presented, defined by statistically significant p values (*p* < 0.05) and/or large effect sizes estimated by Cohen’s D. Where categories are “0/1”, “0” indicates the absence of the symptom in the clinical history, and “1” indicates that the symptom was recorded. “Category n” shows the number of samples available in each category. Positive values of the Cohen’s D value indicate a positive correlation between the first category of the independent variable and the dependent variable (i.e. category “0” of Depression and Cx43 in the parietal cortex are positively correlated—the more Cx43 is in the parietal cortex, the more likely that the value for Depression would be “0”, or no depression); and negative values of the Cohen’s D value indicate a negative correlation between the first category of the independent variable and the dependent variable (e.g. category “limbic” of LB disease type and Cx43 puncta in the frontal cortex—the more Cx43 puncta there are in the frontal cortex, the less likely that the value for the LB disease type would be “limbic”). WB—Western blot; PD—Parkinson’s disease; Cx43—connexin 43; LB—Lewy body; Par Cort—parietal cortex; Front Cort—frontal cortex; Ins Cort—insular cortex; Globus P—globus pallidus; Stri—striatum, Mid SN—midbrain substantia nigra. The full dataset can be explored further using an interactive web application: (https://www.cx43pd.com).

Depression showed the highest correlation with Cx43 decrease in the frontal and parietal cortices; for the latter, the trend was evident at both the level of GJ puncta and Triton X-100-soluble protein levels. Lower Cx43 protein levels were predictors of sleep disturbance across brain regions. Cx43 puncta downregulation in the insular cortex showed a strong correlation trend with memory problems. On the other hand, higher Cx43 in the parietal cortex (protein levels) and globus pallidus (puncta) showed a trend towards correlation with aggression (Table 1).

## Morphological complexity analysis of astrocytes in the frontal cortex

Previous research using induced pluripotent stem cell (iPSC)-derived astrocytes indicated that PD-associated mutations in leucine-rich repeat kinase 2 (LRRK2) led to decreased morphological complexity of the resulting cells^45^. To examine this feature in the present dataset, astrocytes of the frontal cortex were stained with GFAP, which labels major branches of the astrocytic process tree. Only the main primary plus secondary branches of the tree (“major processes”) were counted per cell.

**Fig. 5:**
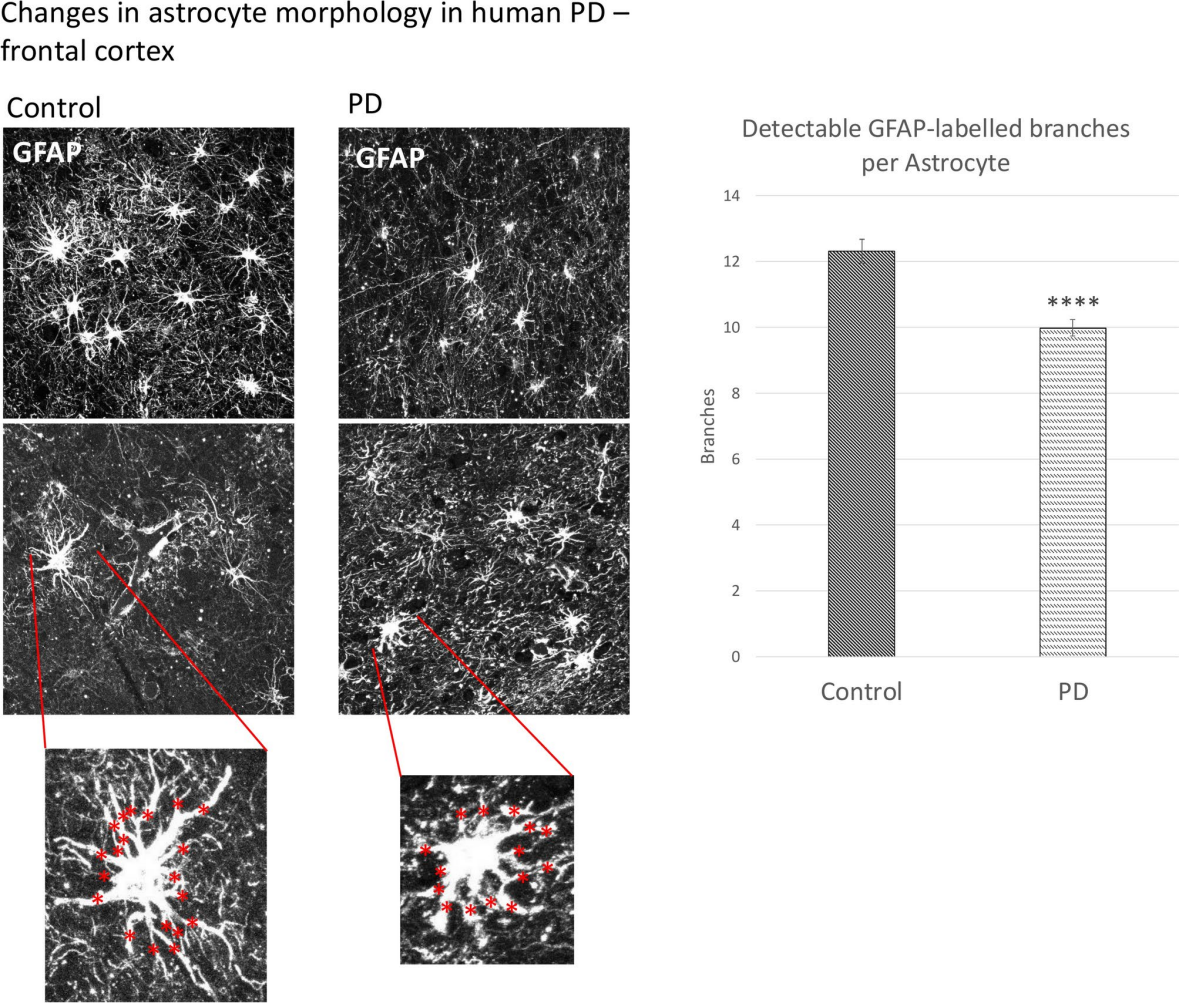
Morphological complexity analysis of astrocytes from the frontal cortex. GFAP staining was performed to identify major branches (primary and secondary), which were manually counted per astrocyte. n = 20 per group, at least 3 microscopic images per sample. Statistical analysis: Mann–Whitney U test; error bars: SEM. ****p = 0.000001225. PD—Parkinson’s disease. GFAP—glial fibrillary acidic protein.

Compared with control samples, PD samples presented with a significantly reduced number of major processes (Mann–Whitney U test due to non-normal distribution, *p* = 0.000001225), indicating a reduced morphological complexity and astrocytic atrophy (Fig. 5).

### GJA1 mRNA analysis in human PD

Previously published sequencing datasets were used to complement our data on Cx43 protein expression to investigate the relationship between GJA1 mRNA and Cx43 protein in PD. For the single nucleus datasets, “astrocytes” were identified by us based on de novo clustering where relevant cell clusters were identified via Aldh1L1 and GFAP co-expression patterns (Suppl. Figure 4).

**Fig. 6:**
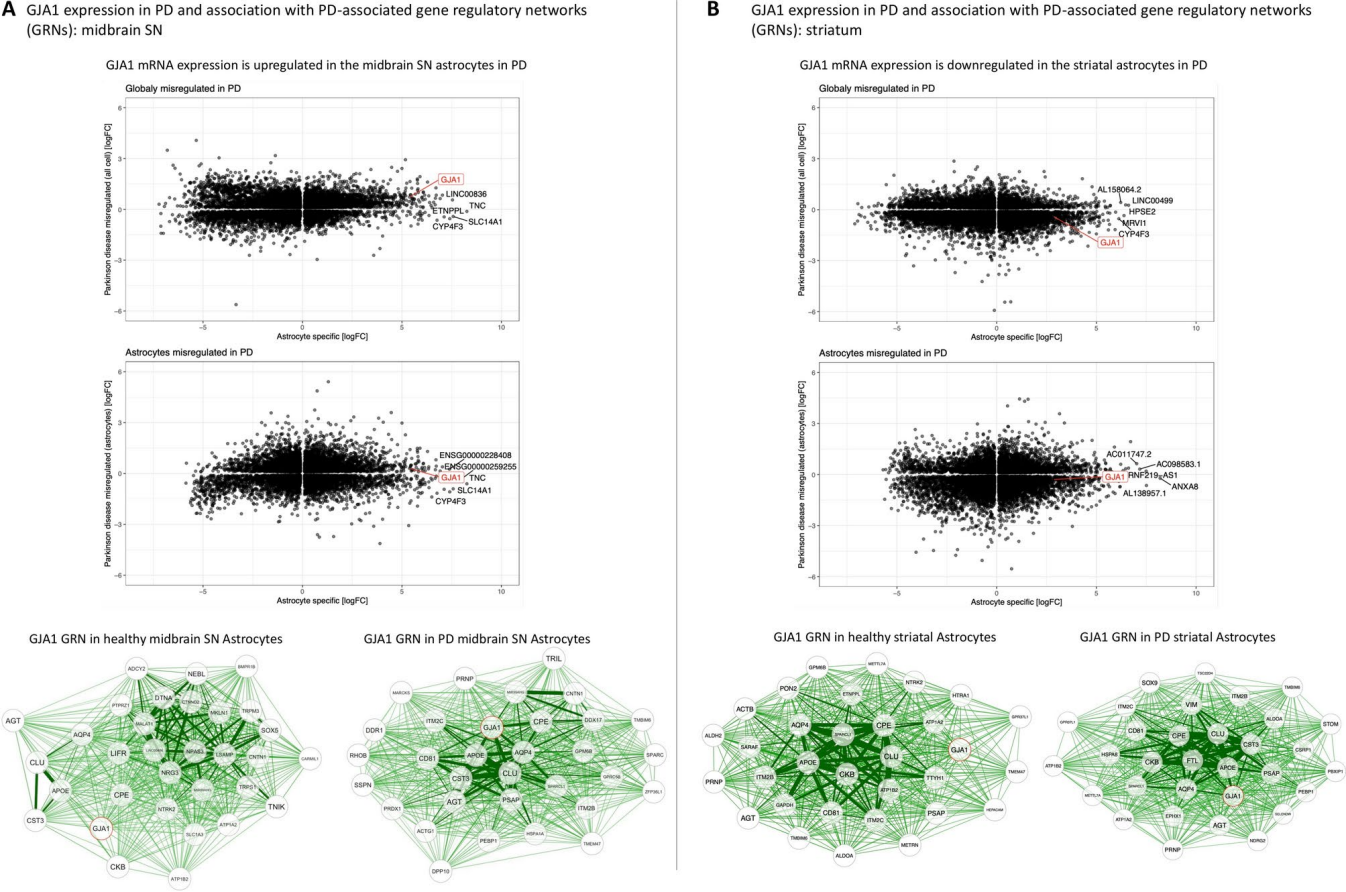
GJA1 (Cx43 encoding gene) mRNA expression patterns in the human midbrain SN and striatum. (**A**) GJA1 expression and gene regulatory networks (GRNs) in midbrain SN-derived astrocytes (n = 6 control individuals, n = 5 PD, re-analysis of a published dataset^13^). GJA1 expression exhibited a trend towards upregulation in PD midbrain SN (FDR-adjusted *p* = 4.320532e-27 for global upregulation, FDR-adjusted *p* = 1 for astrocyte-specific upregulation). (**B**) GJA1 expression and GRNs in striatal astrocytes (n = 4 control individuals, n = 4 PD, re-analysis of a published dataset^14^). GJA1 expression was downregulated in the PD striatum (FDR-adjusted *p* = 0.0001038676 for global downregulation, FDR-adjusted *p* = 1.959544e-07 for astrocyte-specific downregulation). Gene Regulatory Network (GRN) network analysis highlighted closer association of GJA1 with APOE, CLU, AQP4, CPE, CST3, and PSAP in PD astrocytes from both regions, and additionally with FTL in the striatal astrocytes. GJA1 is highlighted in a red circle on both types of diagrams. PD—Parkinson’s disease; Cx43—connexin 43; midbrain SN—midbrain substantia nigra.

A single nucleus sequencing dataset from the midbrain SN (n = 6 controls and n = 5 PD) of late-stage PD patients revealed that GJA1 expression was elevated (FDR-adjusted p = 4.320532e-27 for global upregulation, FDR-adjusted *p* = 1 for astrocyte-specific upregulation) in the PD cohort^13^; the discrepancy between the global vs astrocyte-specific significance values could be due to the low number of cells in the “astrocyte” group (Fig. 6) since re-analysis of a larger midbrain SN dataset^46^ (n = 14 controls and n = 15 PD) revealed a significant GJA1 mRNA upregulation in PD (Suppl. Fig. 5, the dataset for the midbrain SN to highlight in the main text was chosen on the basis of its similarity with the striatal dataset in terms of the patient and cell numbers to make statistics more comparable). On the other hand, analysis of another single nucleus sequencing experiment performed in the striatum^14^ (n = 4 controls and n = 4 PD) revealed that GJA1 expression in PD was significantly decreased (FDR-adjusted *p* = 0.0001038676 for global downregulation, FDR-adjusted *p* = 1.959544e-07 for astrocyte-specific downregulation). These findings corroborated our observation of the regional heterogeneity of the astrocytic response to PD.

To address whether GJA1 expression changes with PD progression, a sequencing array experiment was re-analysed where bulk (not astrocyte-specific) gene expression in the midbrain SN of the control cases (n = 8) was compared with that in early-stage PD (Braak stages 1–2, n = 5), mid-stage PD (Braak stages 3–4, n = 7), and late-stage PD (Braak stages 5–6, n = 8). This revealed that GJA1 expression in the SN increased gradually with PD progression, but this increase did not reach significance in the bulk sequencing dataset^47^ (Suppl. Fig. 3).

In addition to the expression levels, de novo gene regulatory networks (GRNs) were constructed for astrocytic GJA1 measured in the single nucleus experiments to decipher possible co-expression patterns between GJA1 and genes known to be associated with PD progression. Both the striatal and midbrain datasets showed that GJA1 consistently became more closely associated with aquaporin 4 (AQP4, a water channel protein the deficiency in which can exacerbate inflammatory astrocyte activation and a-syn aggregation^48^), apolipoprotein E (APOE, protein involved in lipid metabolism with varied effects on neurodegeneration based on the allele^49^), clusterin (CLU / APOJ, an extracellular chaperone protein that can reduce a-syn aggregation^50^), cystatin 3 (CST3, a cysteine protease inhibitor with dopaminergic neuroprotective effects^51^) and carboxypeptidase E (CPE, involved in protection from dementia and depression^52^) as well as brain-derived neurotrophic factor (BDNF, biogenesis^53^) in PD samples compared to controls (Fig. 6). This could represent the activation of protective mechanisms by Cx43-expressing diseased astrocytes. The increased association of GJA1 with ferritin light chain (FTL, an iron storage protein that can promote NFκB activation^54^) expression in the striatal astrocytes may represent an inflammatory response, whereas a tendency towards closer relationship between prosaponin (PSAP, a protein with roles in lipid homeostasis and anti-parkinsonian effects in models^55^) and GJA1 expression in both regions could be indicative of an increased lysosomal degradation of misfolded proteins in PD astrocytes.

## Discussion

The present work describes a novel aspect of PD pathology that is not directly associated with dopaminergic denervation—Cx43 dysregulation across the brain. There are two main differentiating features of this dataset: first, the Cx43 pathway was investigated in the context of human disease as opposed to animal or in vitro models; and second, brain tissue was sampled from multiple brain regions including the cortex as opposed to focussing on the ventral midbrain.

Cx43 loss in PD was most pronounced across the cortical areas, where it was strongly correlated with depression. Depression has been linked to Cx43 deficits through previous research as decreased Cx43 was found in the post-mortem brains of people diagnosed with major depressive disorder and suicide completers^56^ as well as in animal models of depression such as chronic unpredictable stress in rats where reduced Cx43 protein levels and GJ coupling were found^57,58^; notably, the cortex and caudate nucleus in particular are the brain regions where Cx43 deficiency has been described^56^, which is consistent with the present findings. Experimental pharmacological blockade of Cx43 GJs in the rodent prefrontal cortex with carbenoxolone, GAP27, and GAP26 led to depressive symptoms^58^, and a number of known antidepressants in addition to novel compounds with antidepressant activity were found to have a tendency to promote Cx43 expression, close HCs, and sometimes enhance GJ opening^58–60^. Alcohol dependence linked to depressive behaviour was also found in association with reduced cortical GJ puncta in humans^61^, and alcohol preference in rats was enhanced through cortical pharmacological Cx43 blockade^62^. Adeno-associated virus (AAV)-mediated overexpression of Cx43 in the hippocampus could increase the resistance of mice to depression-like behaviours after early-life stress through maternal separation as assessed via the sucrose preference test, forced swim, and Morris water maze^63^. Moreover, genetic Cx43 deficiency in the mouse hippocampus was associated with anxiolytic effects in another study^64^, potentially highlighting the duality of HC vs GJ functions of the protein where excessively open hippocampal HCs are deleterious such as in a mouse model of prenatal LPS exposure^65^. Depressive symptoms affect around 50% of people with PD with a negative effect on the quality of life^66^, and on the basis of the evidence presented above, Cx43 system dysfunction in the PD cortex possibly plays a role in the development of this symptom.

Important links between Cx43, depression, and inflammation have been noted^67^. For example, increased levels of pro-inflammatory cytokines were found in the cerebrospinal fluid (CSF) of depressed individuals^68^, and experimentally-administered pro-inflammatory factors are known to induce depressive behaviours in humans and animals^69^. Cx43 system disruption, particularly abnormal HC opening, is linked to inflammasome activation followed by increased cytokine release^70^, while complete knock-out of gap junctional proteins Cx43 and Cx30 in the mouse hippocampus also led to astrocytic and microglial activation^8^. At the same time, diverse pro-inflammatory factors such as LPS, IL1β, and TNFα, downregulate Cx43 expression, open HCs, and promote GJ closure^71,72^. Increased pro-inflammatory cytokine levels have been detected in the CSF of people with PD^73^; thus, there is a possible link between an abnormal immune response in PD and dysregulation of the Cx43 system. Increased microglial infiltration has been suggested in the midbrain SN and amygdala in PD^44^, but in our dataset, the greatest extent of Cx43 loss was observed in the cortical regions. We measured the levels of a microglial protein marker Iba1 in the parietal cortex to assess the possibility of enhanced microglial infiltration into this region in PD, which could potentially lead to a more severe decrease in Cx43 levels. Curiously, the anti-correlation between cortical Iba1 and cortical Cx43 loss in PD was rather mild, and instead, a strong correlation with Cx43 puncta loss in the caudate region was evident. It is possible that despite the lack of increased microglial infiltration in the PD cortex, infiltration by other immune cells and/or increased cytokine release by resident brain cells such as microglia, neurons, or astrocytes themselves could contribute to a pro-inflammatory cortical milieu. Further studies of brain and CSF cytokines in PD in correlation with Cx43 will help elucidate possible mechanisms underlying extensive cortical Cx43 depletion. Notably, despite the trend toward an anti-correlation between Iba1 and Cx43 expression in PD, this trend was reversed in the control brains. This observation is consistent with the body of literature on the dual roles of microglia, which can exert protective effects under healthy conditions and promote degenerative processes in disease^74^.

The correlation between cortical Cx43 loss and aggregated protein inclusions was rather mild. A-syn pathology is typically found across the cortex in late-stage PD, often co-occurring with amyloid and tau inclusions, as was the case in many samples from our dataset; however, it was shown that the extent of the cortical Lewy pathology may not directly correlate with the degree of cognitive impairment^75^. On the other hand, oxidative stress, mitochondrial damage, and abnormal metabolism, such as decreased glucose uptake, are found in the Parkinsonian cortex earlier in disease^75^. Abnormal Cx43 HC opening has been shown to contribute to oxidative stress^76^; Cx43 coupling regulates nutrient and metabolite distribution within brain cell networks^77^; and mitochondrial Cx43 regulates adenosine triphosphate (ATP) production^78^—making imbalance in the HC vs GJ parts of the Cx43 pathway a possible cause underpinning early cognitive changes. Inclusion of earlier PD stages in future work will be conducted to understand causal relationships.

Memory problems showed a strong correlation trend with a large effect size for Cx43 puncta loss in the insular cortex, which is the region of the brain involved in object recognition and social memory. Changes in memory and thinking are experienced by approximately half of the people with PD within 10 years of diagnosis according to Parkinson’s UK, and could represent initial changes leading to PD dementia. Cx43-coupled astrocytic networks have been previously studied in the context of hippocampal memory formation in rodents where experimental astrocytic decoupling via Cx43/Cx30 knock-out led to the disruption of memory acquisition^8^. Extending the post-mortem Cx43 study in human PD to a wider range of regions, including the hippocampus, will shed more light on the correlations between memory loss and astrocytic coupling in future studies.

Sleep disorders are another extremely common non-motor PD symptom category affecting up to 2/3rds of people with PD. While some sleep disturbances could be attributed to the side effects of antiparkinsonian medications, it is also possible that inherent PD pathophysiology contributes to abnormal sleep patterns. Cx43 protein downregulation in the striatum showed the strongest degree of correlation with sleep disturbance in our dataset, and Cx43 protein expression and GJ puncta downregulation in other regions presented with correlation trends with this symptom. Accordingly, published literature suggests that Cx43 networks are involved in sleep–wake cycle regulation in rodents and that their disturbance can lead to affected sleep patterns^77^; this is corroborated by findings in humans in which chronic insomnia was shown to be associated with decreased plasma Cx43 levels^79^. It must be noted that “sleep disturbance” is a broad and multifactorial term which is likely to have a number of causes beyond a single cell type or a protein pathway. In this study, this term was used to summarise diverse clinical notes where observations were not conducted under the same conditions, which presents a limitation.

Interestingly, higher Cx43 in some regions showed positive correlation trends with aggression. Given that aggression could result from anti-Parkinsonian medication side effects, further research into drug interactions with the Cx43 pathway is needed.

Overall, correlations between Cx43 dysregulation and symptoms should be interpreted with caution as these alone do not indicate causation. This study will be supplemented by further functional work using Cx43-modulating interventions, for example with Cx43-modulating compounds that distinguish between GJ and HC states such as GAP19, as well as total Cx43 pathway blockers like GAP27 and Cx43/GJA1 knock-down using shRNA. In vivo models of PD are often poor predictors of NMS^80^, so a careful consideration of human-relevant model systems is required.

All PD patients in our dataset presented with motor and gastrointestinal (GI) symptoms; thus, a correlation between Cx43 downregulation and these most common PD manifestations could not be established. Of note, mice with astrocyte-directed Cx43 deletion presented with decreased motor coordination in the rotarod test^9^, which resembles the Parkinsonian phenotypes often observed in PD models, and hippocampal deletion of Cx43 and Cx30 resulted in impaired sensorimotor performance in mice^8^. Cx43 was also found to play key roles in gut motility^81^, and GI transit decreases in PD. As the next step, we will extend the characterisation of Cx43 deficits in human PD to earlier PD stages and prodromal PD when motor and GI symptoms are not yet ubiquitously present.

Surprisingly, Cx43 disturbance in PD was most pronounced outside of the midbrain SN—the brain region most widely studied in the context of PD. This result was corroborated by our bioinformatics analysis of the single-cell sequencing datasets where GJA1 expression was decreased in the PD striatum^14^ but slightly increased in the PD SN^13^ compared to healthy controls. There are two main non-exclusive explanations. First, our dataset specifically captured late-stage PD, which may not represent the mechanisms *driving* the disease progression but rather the *aftermath* of many decades of pathology. Given that a-syn inclusions tend to affect the SN prior to other brain areas, such as the cerebral cortex, it is possible that the SN in late-stage PD presents with a “burnt-out” pathology. The Cx43 system might have thus been disrupted in the SN for many years, which could lead to the paradoxical mRNA and protein expression upregulation by resident astrocytes in a compensatory attempt, as it was shown in cultured astrocytes that pharmacological Cx43 blockade or metabolic stress due to growth in high glucose can lead to a compensatory increase in Cx43 expression despite functional GJ block^37,82^. The increased GJA1 mRNA co-expression in the SN with other neuroprotective genes such as CLU, CST3, CPE, AQP4, and PSAP, revealed by the GRN analysis of PD astrocytes, might suggest that Cx43 is expressed as part of the protective response gene network. In accordance with the view of a possible functional dysregulation despite continued Cx43 protein presence, it was noted that the midbrain SN samples of the PD cohort presented more frequently with a Cx43 immunostaining pattern that was not suggestive of the GJ staining but rather of a more uniform “filled” labelling of the cell bodies and processes. This finding is consistent with observations in an inflammatory model of PD induced by LPS injection into the SN in rats, where SN astrocytes presented with an enhanced, but more diffuse Cx43 immunoreactivity^29^. The cells with the “filled” Cx43 appearance were not excluded from the automated Cx43 puncta counting to keep the analysis unbiased; however this type of analysis does not differentiate between true GJ plaques, fragments of “filled” processes with varying fluorescence intensities, and internalised Cx43 which might be destined for degradation (annular junctions). At the same time, some GJ plaques can be functionally open despite their small size. Further studies using more specific GJ vs HC detection techniques would be necessary to address these issues.

Second, astrocytes are highly heterogenous among brain regions^83^, and it is feasible that, compared with cortical cells, midbrain astrocytes have unique responses to PD. This view is corroborated by the observation that midbrain astrocytes presented unique protein co-expression patterns where increased Cx43 showed correlation trends with higher GFAP, whereas cortical and striatal astrocytes presented the opposite trend.

In either case, it must be noted that Cx43 mRNA and protein expression do not directly correlate with the functions of the Cx43 system since there are few reliable markers for the open vs closed state of GJs and HCs that can be studied in fixed tissues. Various phosphorylation sites on Cx43 have been proposed as “gatekeepers” for open Cx43 GJ states, such as S368^84^ or S365^85^, but these observations were often found to be non-reproducible based on the model and/or region used. Furthermore, studying post-translational modification (PTM) states of proteins is particularly challenging in human post-mortem tissues where the post-mortem interval can be large, is not always recorded, and is very difficult to control for, which can lead to PTM degradation. As the next step, conformation-specific Cx43 antibody staining^86^ can be explored in PD vs controls.

Astrocytic atrophy with a marked decrease in morphological complexity has been described in iPSC-derived cells with PD-associated LRRK2^(G2019S)^ mutation *in vitro*^45^, and we sought to validate this observation in human PD. GFAP staining, which reveals the major astrocytic process tree, was chosen for this analysis; staining was conducted in the frontal cortex since cortical astrocytes present with clearer non-overlapping domains. There are three main technical challenges associated with the morphology analysis: generally higher GFAP levels in PD which might skew the results of the analysis (for example, if finer branches are more clearly labelled in PD but are not visible in controls due to lower GFAP), the depth of the slices (5 μm), which does not capture the whole astrocytic process tree, and potential morphological heterogeneity among astrocytic subtypes^87^, which may lead to uneven subtype selection across individuals due to the lack of subtype biomarkers. Nevertheless, cortical astrocytes in PD patients presented with a simplified morphology on average, which was analysed semi-quantitatively via manual major branch, or process, counting. The reduced complexity of the astrocytic tree alongside decreased Cx43 expression aligns with the stabilising function of GJs^88^, where atrophic cells are likely to make fewer process contacts with neighbouring cells in a network and thus less likely to participate in extensive coupling. Reduced astrocytic complexity has also been reported as a feature of aged cortical astrocytes in humans^89^ and rodents^90^ alongside decreased GJ coupling; this ageing-related process might be enhanced in PD. Subsequent studies using Cx43/GJA1 knock-down tools and GJ decoupling compounds will help elucidate any causal relationship between Cx43 and astrocytic morphology.

## Conclusions

The present report describes brain region-specific Cx43 downregulation and dysfunction in human PD, which is likely to also result in an imbalance between GJ and HC components of the Cx43 system. Cx43 disruption was correlated with key NMS of PD. Further functional studies will help elucidate whether the restoration of GJ vs HC balance in PD could prove disease-modifying. Inclusion of earlier stage PD cases in future studies will establish the causality between Cx43 loss and other PD hallmarks like a-syn aggregation. These data also emphasise the crucial importance of the PD model choice when studying disease mechanisms or disease-modifying treatments since Cx43 downregulation is not evident in rotenone or MPTP models.

## Methods

### Demographics of the patient and control populations for post-mortem tissue analysis

Human post-mortem samples were obtained from the Imperial College London Multiple Sclerosis and Parkinson’s Tissue Bank.

All experimental protocols involving human data and samples were approved by the national Wales Research Ethics Committee. All methods including sample handling and storage were carried out in accordance with the Human Tissue Act (HTA, 2004 UK) and in compliance with the 1964 Helsinki Declaration and its later amendments.

Written informed consent for the use of brain tissue in research and publication of the results was obtained from all brain tissue donors during life. Patients’ identities have been encoded and anonymised, and no personally identifiable images nor other personal details are included in this publication.

**Fig. 7:**
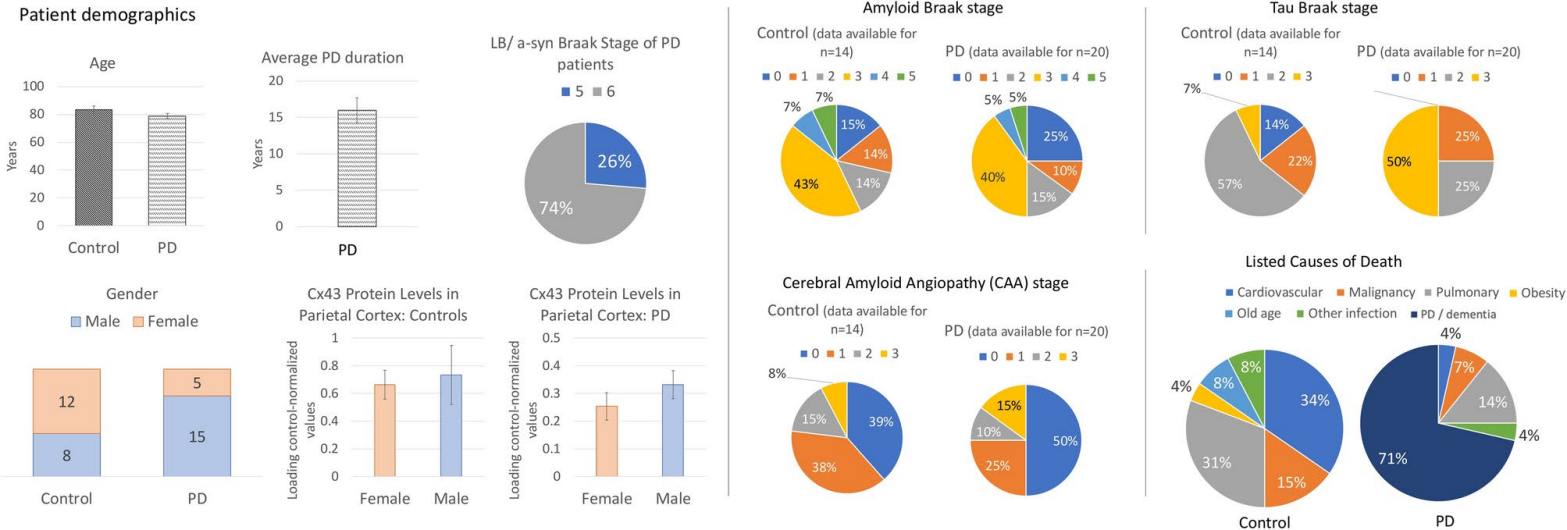
Patient demographics. A total of 20 control and 20 age-matched PD patients were included in the study with an average age of approximately 80 years. A greater prevalence of males in the PD group was noted, but no significant gender effect on the brain Cx43 levels was observed. Braak stages for Lewy Body / α-synuclein, amyloid, and tau, as well as cerebral amyloid angiopathy scores, were recorded. Listed causes of death were summarised with cardiovascular and pulmonary causes most prevalent in the controls, and PD or PD-related dementia being the leading cause of death in the patient group. Statistical analysis: t-tests; error bars: SEM. PD—Parkinson’s disease; Cx43—connexin 43; LB—Lewy body; a-syn—α-synuclein.

Patients’ and controls’ demographic and clinical characteristics were examined alongside the neuropathological reports on other aspects of brain pathology including Braak stages and region-specific scores for Lewy Body (LB)/a-syn, amyloid, and tau; SN depigmentation; and CAA (Suppl. Table 1, Fig. 7). The control (n = 20) and PD (n = 20) groups were age-matched (83y.o. on average in the control group vs 79y.o. for PD) and had relatively similar patterns of amyloid pathology Braak stages as well as CAA scores; the PD group had a higher average tau Braak stage compared to controls (50% score 3 in PD vs 7% in controls). PD patients in this dataset presented with a late-stage/advanced PD corresponding to Braak stages 5–6 (74% stage 6) where PD was diagnosed for approximately 15 years on average, all of whom experienced motor and gastrointestinal symptoms and received dopamine-replacement as well as other symptomatic treatments (full clinical histories were available for examination in 18 PD cases and 14 controls). The PD group also presented with various NMS including cognitive impairment, depression, memory problems, sleep disturbance, aggression, hallucinations, and psychotic symptoms (Suppl Table 1). The PD group contained a greater percentage of males (75% in the PD group vs 40%, in the control group), so Cx43 levels were compared between genders in both groups to eliminate the possibility that the gender imbalance may have masked the study results; no significant gender effects on cortical Cx43 protein levels were found with a slight trend toward greater Cx43 in males (Fig. 7), and a similar Cx43 distribution was observed in other brain regions (data not shown). Thus, any PD-related downregulation of Cx43 is unlikely to be due to the higher prevalence of males in the PD group.

Cardiovascular and pulmonary diseases were the leading causes of death in the control group (34% and 31%, respectively) whereas PD or PD dementia were the leading causes of death in the patient group, accounting for 71% of the listed causes of death (Suppl. Table 1, Fig. 7). Extended notes on the clinical presentations and medications taken during life can be found in Suppl. Table 2.

## Immunofluorescence staining of human brain tissue

The human brain samples used for imaging were supplied as formalin-fixed paraffin-embedded (FFPE) 5 μm-thick brain slices (three sections per patient per brain region). The sections were deparaffinised and rehydrated via incubation at 60 C° for 2 h followed by incubations at room temperature for 10 min each in xylene × 2, 100% ethanol, 96% ethanol, 70% ethanol, 50% ethanol, and phosphate-buffered saline (PBS). Antigen retrieval was performed in the Tris-ethylenediaminetetraacetic acid (EDTA) pH 9 buffer (Abcam) followed by blocking and permeabilization in the buffer containing 5% donkey serum (Abcam), 5% bovine serum albumin (BSA, Sigma), and 0.5% Triton X-100 (Sigma) in PBS for 1 h at room temperature. Primary antibody incubation was conducted in a staining buffer containing 2% donkey serum, 2% BSA, and 0.3% Triton X-100 for 48 h at 4 C° followed by three 1 h washes with PBS. Lipofuscin quenching was performed using TrueBlack (Biotium), followed by a secondary antibody incubation in a staining buffer containing 2% donkey serum and 2% BSA for 20 h at 4 C°. Nuclei were visualised with 4′,6-diamidino-2-phenylindole (DAPI, Thermo Fisher Scientific) added to the secondary antibody solution. Stained brain slices were mounted in FluoroMount-G (Thermo Fisher Scientific), coverslipped, and imaged via confocal microscopy in photon counting mode using a Stellaris (Leica) microscope to ensure minimal batch-to-batch variability. The samples was split into six staining batches; each staining batch contained an equal number of PD patients and control individuals. The images were processed via ImageJ software and analysed statistically. Patients’ identities and disease statuses were coded, and researchers conducting the analysis were blinded to the experimental conditions. 20 individuals were included in each group (PD patients and age-matched controls).

Primary antibodies used for fluorescence immunostaining:

- Rabbit anti-connexin43 (Abcam ab11370) 1:500
- Mouse anti-Sox9 (Abcam ab76997) 1:250
- Chicken anti-glial fibrillary acidic protein (GFAP, GeneTex GTX85454) 1:500

Secondary antibodies used for fluorescence labelling:

- AlexaFluor 488 and 568 raised in donkey (Invitrogen) 1:500

Dyes used for fluorescence staining:

- DAPI (Sigma D9542) 10μg/ml

## Microscopic analysis of the gap junctional puncta

Gap junctional puncta were identified in Fiji/ImageJ (version 2.1.0, https://imagej.net/ij/) via the “3D object counter” functionality. Thresholds were set to 10–100 voxels (size threshold) and 150 (intensity threshold) using a negative staining control sample (secondary antibody only) as a guide, then the same intensity and particle size thresholds were propagated to analyse all images.

## Western blot protein analysis

The brain tissue samples were weighed and homogenised via a glass Dounce homogeniser in 1:4 w:v lysis buffer (with Thermo Scientific Halt protease inhibitors) and collected in tubes for further incubation on ice overnight to complete the lysis. The lysates were then centrifuged at 4 C° for 10 min at 10,000 g, and the supernatants were saved and analysed for protein concentration via the Pierce Bicinchoninic Assay kit (Thermo Fisher). Normalised protein amounts (15 μg) were then prepared for a denaturing SDS-PAGE analysis by mixing the samples with a NuPAGE sample reducing agent and NuPAGE LDS sample buffer (all Invitrogen), heating to 95 C° for 5 min, and running on the 4–12% NuPAGE mini gels (Invitrogen) followed by a transfer to a Cytiva Amersham Hybond 0.45 μm pore size PVDF membrane (VWR International). A Precision Plus protein molecular weight ladder (Bio-Rad) was run alongside the experimental samples to confirm the molecular weights. Protein-containing membranes were stored in TBST and analysed within 1 week. The total protein content per lane was estimated prior to antibody staining via Revert Total Protein stain (Li-Cor). Non-specific antibody binding to the membranes was blocked by incubation in 5% non-fat milk in TBST for 1 h at room temperature, followed by an incubation with a primary antibody and an horseradish peroxidase (HRP)-conjugated secondary antibody each for 1 h in 1% non-fat milk in TBST at room temperature, with TBST washes after each antibody incubation. The protein bands were visualised via Enhanced Chemiluminescence (ECL) Prime reagent (VMR International) in a GelDoc imaging system (Bio-Rad). Semi-quantitative densitometry analysis of the bands was conducted via Image Studio Lite software (version 5.2.5, https://www.licorbio.com/image-studio). The signals of the bands were normalised to the total protein content of the relevant lanes.

To compare batches of samples ran of separate membranes, an Expression Control sample was introduced per each membrane which was derived from a control parietal cortex sample. All other samples per given membrane were then normalised to the Expression Control, which remained the same for all the Western blot membranes.

Lysis buffers used for western blotting:

For the determination of the Triton X-100-soluble Cx43 fraction, the Non-Denaturing Lysis Buffer (20 mM Tris–HCl, 137 mM NaCl, 1% Triton X-100, 2mM EDTA, pH to 8) was used. Halt Protease Inhibitor Cocktail (Thermo Scientific) was added to all the lysates.

Primary antibodies used for western blot immunostaining:

- Rabbit anti-connexin43 (Abcam ab11370) 1:1000–1:2500
- Rabbit anti-Aldh1L1 (Abcam ab190298) 1:1000
- Mouse anti-GFAP (ProteinTech 60,190–1) 1:5000
- Rabbit anti-Iba1 (Abcam ab178846) 1:1000

Secondary antibodies used for western blot immunostaining:

- Goat anti-mouse HRP-conjugated (Agilent P044701-2) 1:5000
- Goat anti-rabbit HRP-conjugated (Agilent P044801-2) 1:5000

## Statistical analysis

Data normality was verified via the Shapiro–Wilk test. The assumption of homogeneity of variance was tested via Levene’s Test. The data were analysed via R^91^ (https://www.R-project.org/), version 4.2.1, repository can be accessed here: https://github.com/WaylandM/Connexin_43_pathology_in_late-stage_human_Parkinsons_brains. Puncta per cell data were transformed to normality via the Box-Cox method implemented in the R package MASS^92^ (https://cran.r-project.org/web/packages/MASS/index.html), version 7.3–58.1. Analysis of variance (ANOVA) was used to identify brain regions where PD patients differed from controls, while controlling for the effects of confounding variables. Briefly, three potential confounders were considered: batch, age of donor and gender of donor. For each brain region, eight candidate linear models were considered, comprising all combinations of the confounders and group (PD patient/control) as the main effects; puncta per cell was the dependent variable in these models. Akaike’s information criterion was used to select the best model. A Type II sums of squares ANOVA, implemented in the R package car^93^ (https://cran.r-project.org/web/packages/car/index.html), version 3.1.0, was used to compare the mean number of puncta per cell in Parkinson’s disease patients and controls for each brain region while controlling for the effects of any confounding variables in the model. The false discovery rate (FDR) was controlled via a previously published procedure^94^. Both raw and FDR-adjusted p-values are reported in the tables. Effect size (ϵ2), the proportion of the variation in puncta per cell explained by group membership (control vs PD), was calculated via the R package effectsize^95^ (https://cran.r-project.org/web/packages/effectsize/index.html), version 1.0.0. Effect sizes were interpreted according to the previously established rules^96^. The same protocol was used to identify which of the assayed proteins (ALdh1L1, Cx43, and GFAP) were differentially expressed in each of the brain regions.

Correlation matrices were constructed to explore the associations between all pairs of continuous and ordinal variables. Spearman’s rank order correlation coefficient was used to measure the strength of any monotonic relationship (linear or non-linear).

Partial data were available for a number of dichotomous symptom variables, namely presence (1)/absence (0) of: dementia (cognitive impairment), depression, sleep disturbance, aggression, hallucinations and psychotic symptoms. Lewy body disease type description (limbic/neocortical) was also available for PD patients. Since each of these variables had missing observations for 1–2 donors, they were not included in the models used to compare the protein expression or puncta counts of the PD and control groups. Instead, an exploratory analysis of the effects of these variables on protein expression and puncta counts was conducted. For each variable, a t-test was used to compare the two categories. If the variances of the two categories were equal, a pooled variance t-test was used; otherwise a Welch t-test was applied. Cohen’s D was computed to measure effect size^95^.

Data collection, but not data analysis, was performed in a blinded manner to the conditions of the experiments. No collected data that matched the technical quality control requirements were excluded from the analysis.

## Bioinformatics analysis

### Data acquisition and pre-processing

Raw single cell expression data in the raw Unique Molecular Identifier (UMI) count matrix, gene metadata and cell metadata format were obtained from GEO for two datasets: (1) GSE161045: “Human striatal glia differentially contribute to AD and PD-specific neurodegeneration”, PD:n = 4, CTRL:n = 4 (https://www.ncbi.nlm.nih.gov/geo/query/acc.cgi?acc=GSE161045) and (2) GSE157783: “Single cell atlas of the human midbrain reveals cell state specific to Parkinson’s disease”, IPD:n = 5, CTRL:n = 6 (https://www.ncbi.nlm.nih.gov/geo/query/acc.cgi?acc=GSE157783).

Both datasets were imported into R and processed via the Seurat package (https://satijalab.org/seurat/), version 5.0.1:

- Normalised counts via the “LogNormalize” method;
- Identified features that are outliers;
- Scaled and cantered features in the dataset, without regressing variables;
- Run Principal Component Analysis (PCA), Uniform Manifold Approximation and Projection (UMAP) and t-SNE dimensionality reduction methods;
- Computed the k.param nearest neighbours using the PCA reduction as input;
- Identified clusters of cells by a shared nearest neighbour (SNN) modularity optimization.

The cluster(s) representing astrocytes were then manually annotated based on marker gene expression (Aldh1L1, GFAP; Suppl. Fig. 4) and checked against the cell ontology provided by dataset authors where such ontologies were available in the cell metadata.

### Differential expression analyses

The features (expression values) were grouped in two ways:

- Astrocyte clusters vs. all other cell-type clusters;
- Parkinson’s disease (PD/IPD) samples vs. control samples.

Differentially expressed genes between two groups of cells were identified via the Wilcoxon Rank Sum test. Statistical significance (p-values) and average log2 fold changes (avg_log2FC) were organised into differential expression tables.

### Correlation analyses—gene regulatory networks (GRNs)

The correlation-based gene networks were nucleated using GJA1 (Cx43) single-cell expression values. The networks were calculated separately for Parkinson’s disease (PD/IPD) samples and control samples.

The input for the graphical model was prepared in the following steps:

- Created an n × m numeric matrix of expression measures representing single-cell RNA sequencing (scRNA-seq) counts, where n is the number of individual cells and m is the number of assessed genes;
- Correlate all genes to the GJA1 (Cx43) expression profile and select the 32 most similarly expressed genes for further analyses based on Pearson correlation coefficient value;
- Create the correlation matrix of these 32 plus GJA1 (Cx43) genes (Spearman ranked correlation coefficient) and use it as the input to build a graphical model estimation network.

### Graphical model estimation for correlation networks

Correlation graphs represent the correlation matrix with nodes that indicate genes of interest and edges that represent correlation values. The green edges indicate positive correlations; and the red edges indicate negative correlations. The width of the edges and their colour saturation correspond to the absolute value of the correlations and scale relative to the strongest weight in the graph. The graphs are organised in a “spring” layout, which uses the Fruchterman–Reingold algorithm to obtain a force-directed layout. In this solution, each node (connected and unconnected) repulses the other, while connected nodes attract each other. After 500 iterations, a final logout is reached; the distance between the nodes corresponds well to the correlation between the nodes, where correlated nodes are close to each other, whereas anticorrelated (negative correlation) nodes are moved to distant parts of the graph.

## Supplementary Information

Supplementary Information 1.

Supplementary Information 2.

## Data Availability

The imaging dataset generated and analysed during this study is available in the BioImage Archive repository, https://www.ebi.ac.uk/biostudies/bioimages/studies/S-BIAD1133 (10.6019/S-BIAD1133). The code used for the statistical analysis is available via GitHub, https://github.com/WaylandM/Connexin_43_pathology_in_late-stage_human_Parkinsons_brains. The Western blot dataset for all Cx43, GFAP, and Aldh1L1 staining is provided as a Supplementary file. All other raw data and analysis details are available from the corresponding author upon reasonable request.

## Abbreviations

a-syn: α-Synuclein
ANOVA: Analysis of variance
APOE: Apolipoprotein E
AQP4: Aquaporin 4
BDNF: Brain-derived neurotrophic factor
BSA: Bovine serum albumin
CAA: Cerebral amyloid angiopathy
CLU: Clusterin
CPE: Carboxypeptidase E
CSF: Cerebrospinal fluid
CST3: Cystatin 3
Cx30: Connexin 30
Cx43: Connexin 43
DAPI: 4′,6-Diamidino-2-phenylindole
ECL: Enhanced chemiluminescence
EDTA: Ethylenediaminetetraacetic acid
FDR: False discovery rate
FFPE: Formalin-fixed paraffin-embedded
FTL: Ferritin light chain
GFAP: Glial fibrillary acidic protein
GI: Gastrointestinal
GJ: Gap junction
GJA1: Gap junction α-1 protein (encoding the connexin 43 gene)
GRN: Gene regulatory network
HC: Hemichannel
HRP: Horseradish peroxidase
iPSC: Induced pluripotent stem cell
LB: Lewy body
LPS: Lipopolysaccharide
LRRK2: Leucine-rich repeat kinase 2
MPTP: 1-Methyl-4-phenyl-1,2,3,6-tetrahydropyridine
NMS: Non-motor symptoms
6-OHDA: 6-Hydroxydopamine
PBS: Phosphate-buffered saline
PCA: Principal component analysis
PD: Parkinson’s disease
PSAP: Prosaponin
PTM: Post-translational modification
SEM: Standard error of the mean
SN: Substantia nigra
WB: Western blot
