# Supplementary material for: Connexin 43 is downregulated in advanced Parkinson’s disease in multiple brain regions which correlates with symptoms
Source: Sci Rep. 2025 Mar 25;15:10250. doi: 10.1038/s41598-025-94188-7 (PMC11937269; doi:10.1038/s41598-025-94188-7)

Western Blot Dataset

For manuscript: “Connexin 43 is downregulated in advanced Parkinson’s disease in multiple brain regions which correlates with symptoms”

Prepared by Dr N. Hastings

Human tissue samples
20 Controls; 20 Parkinson’s Disease

Parietal cortex batch 1: 1-6

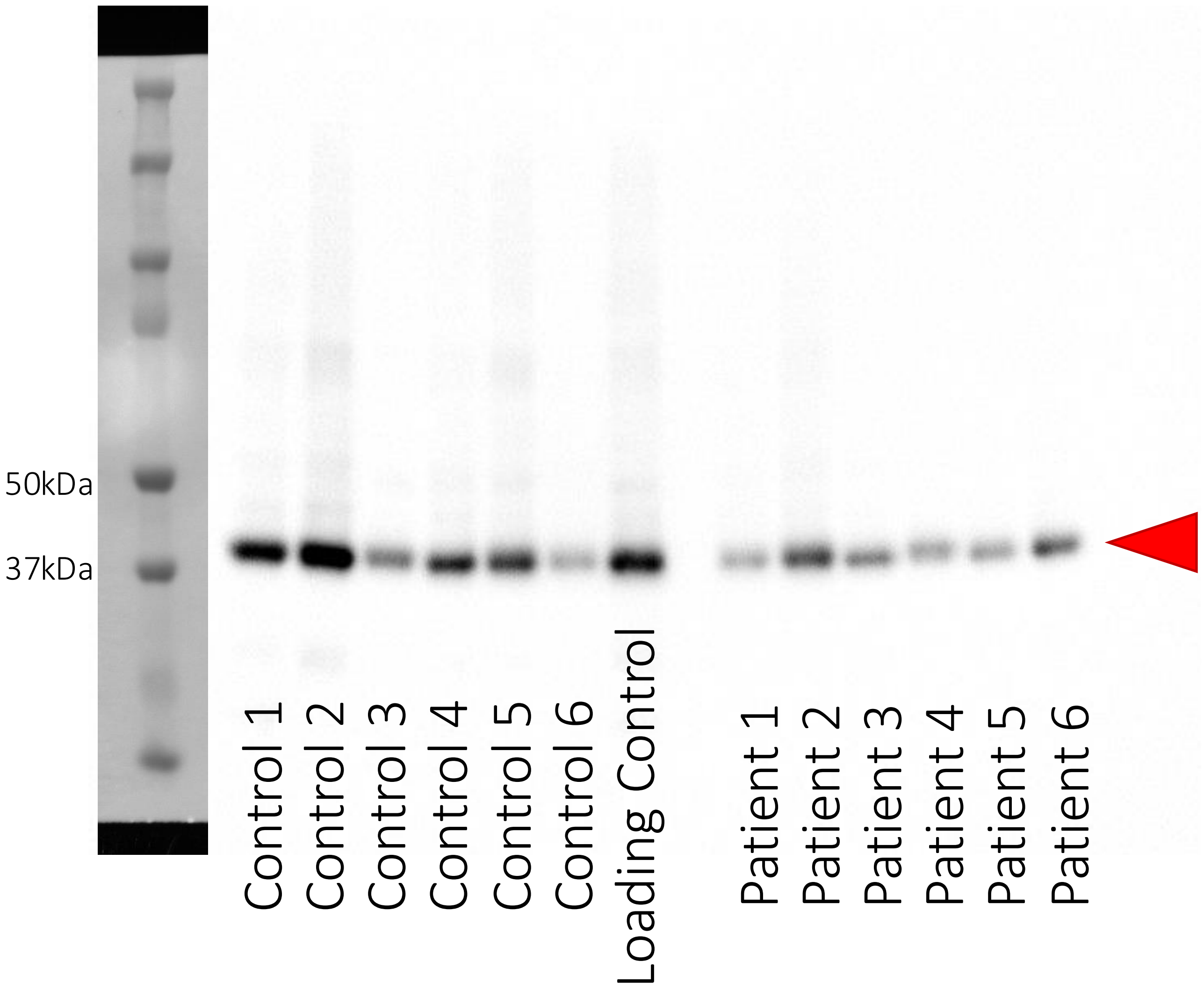

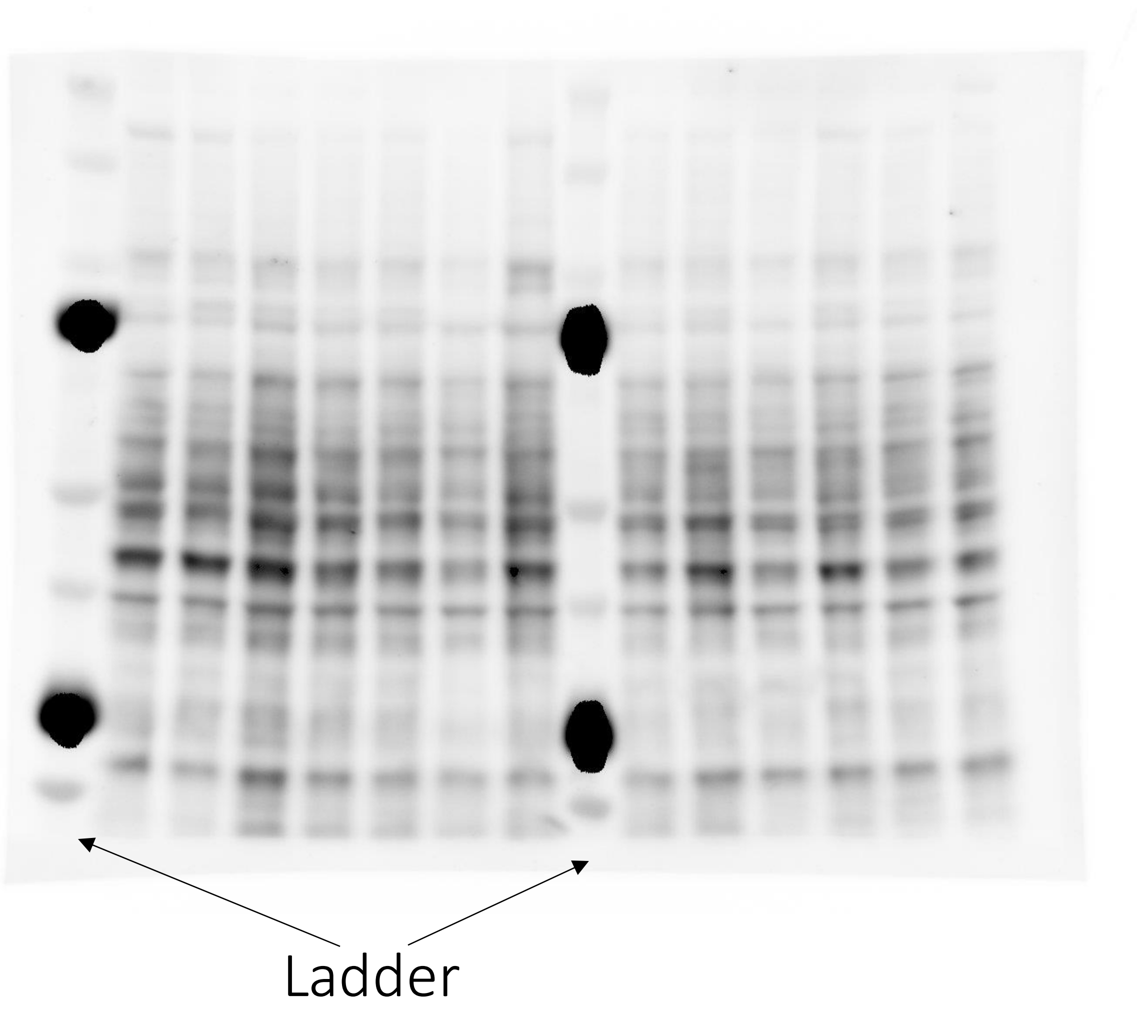

Parietal cortex batch 1: 1-6

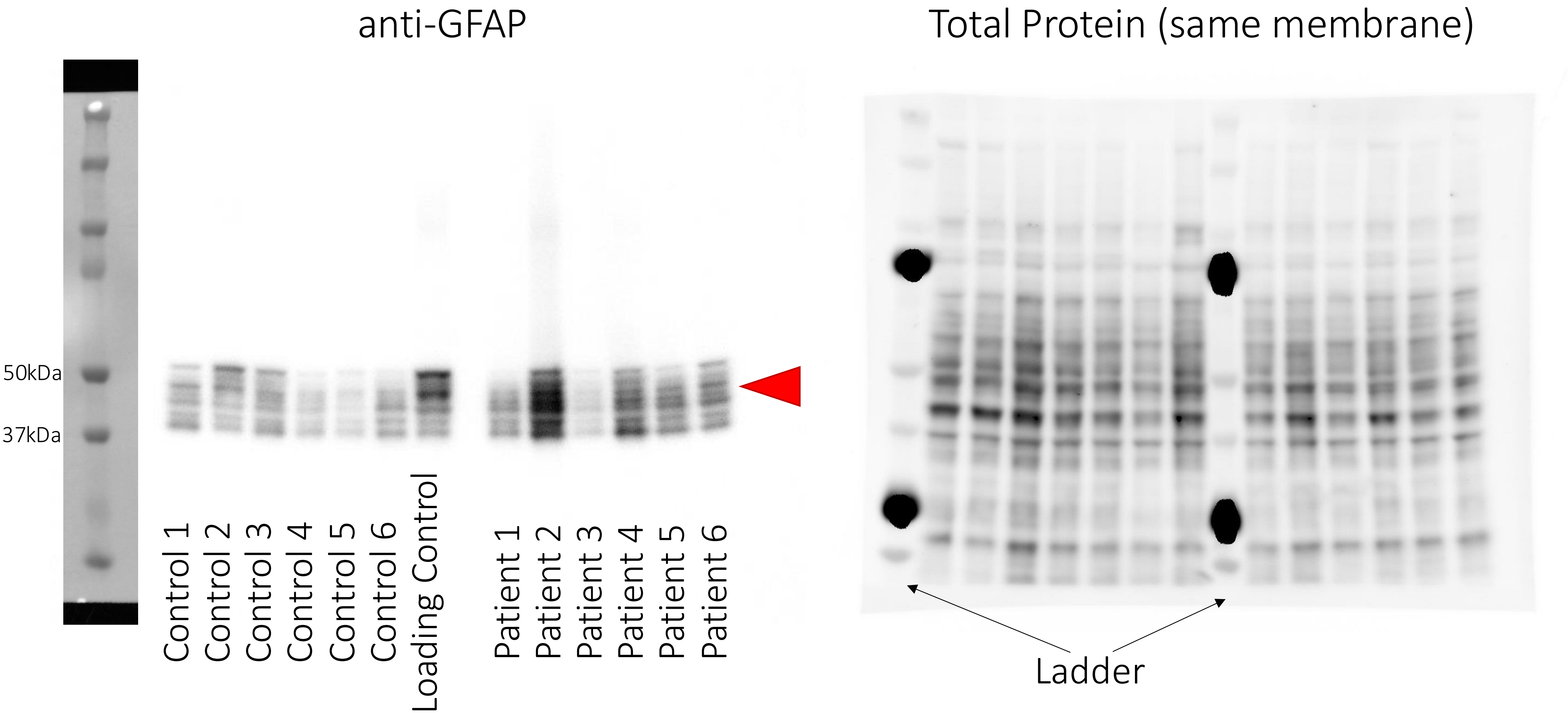

Parietal cortex batch 1: 1-6

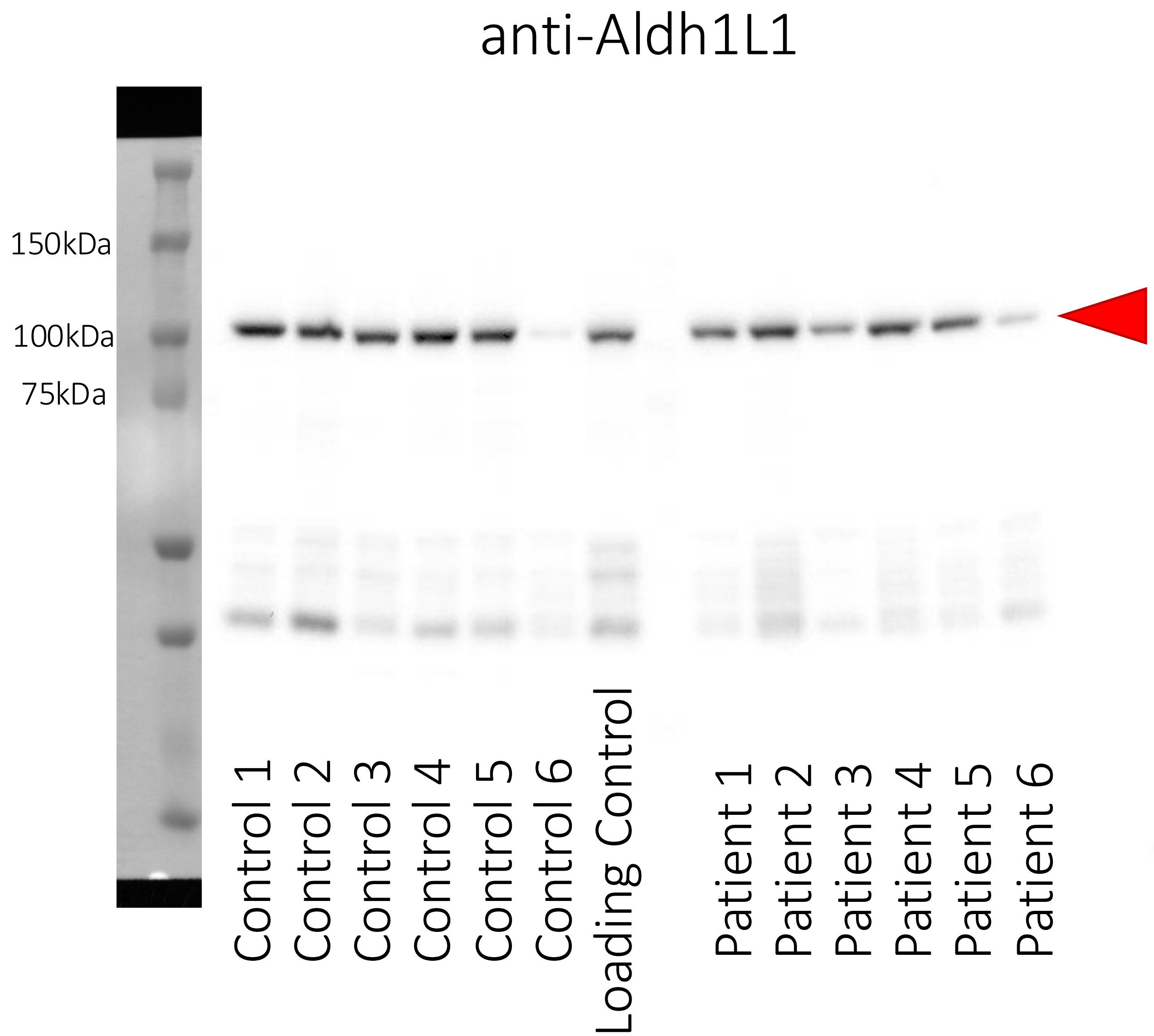

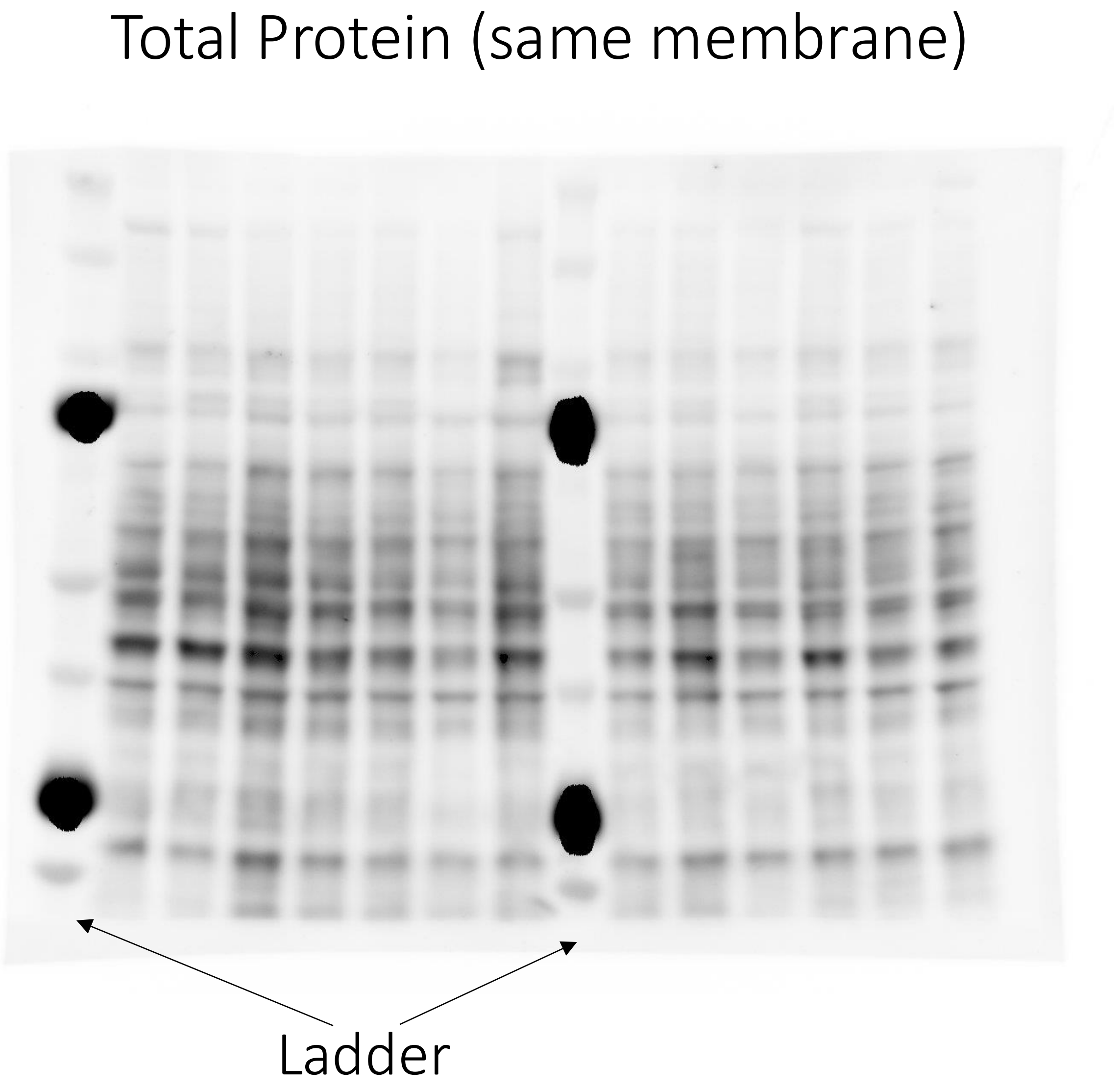

Striatum batch 1: 1-6

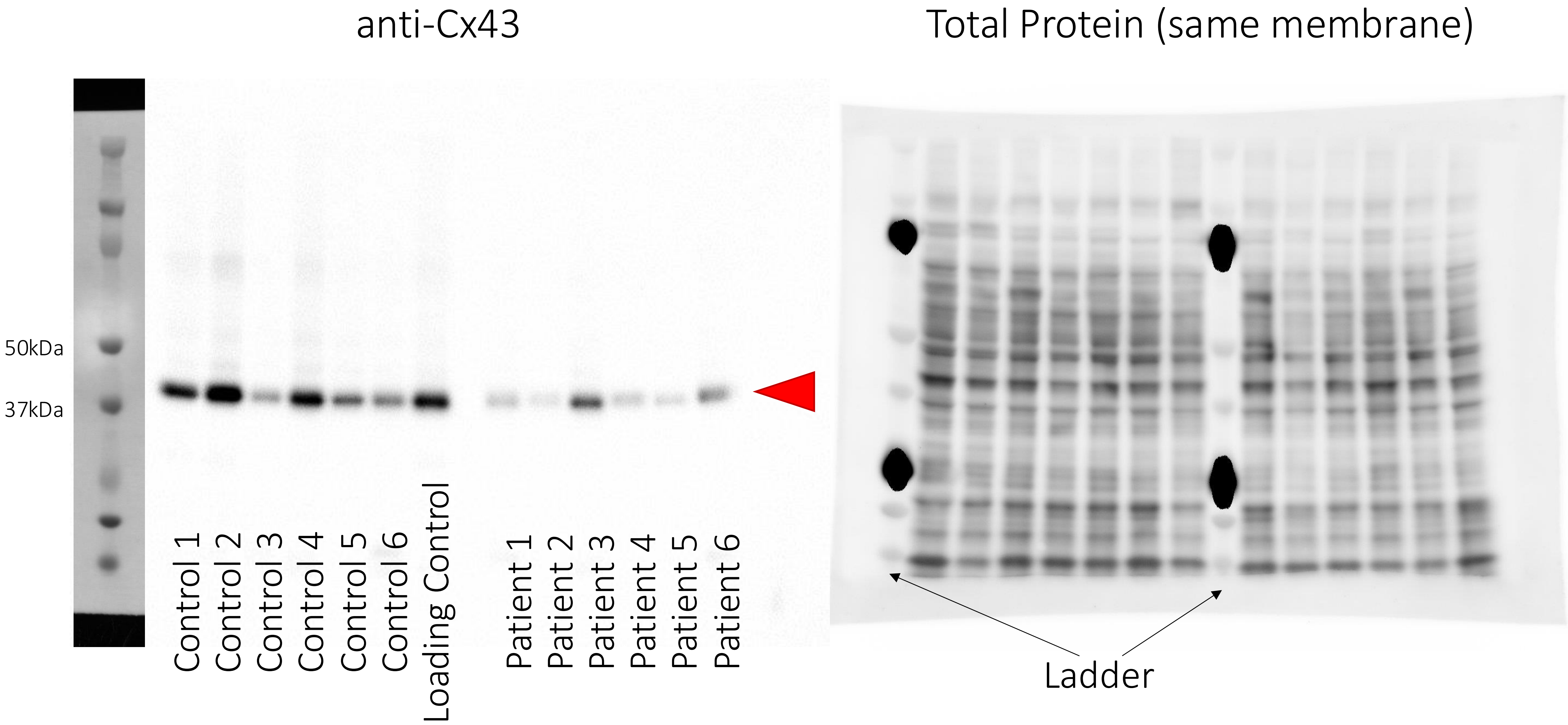

Striatum batch 1: 1-6

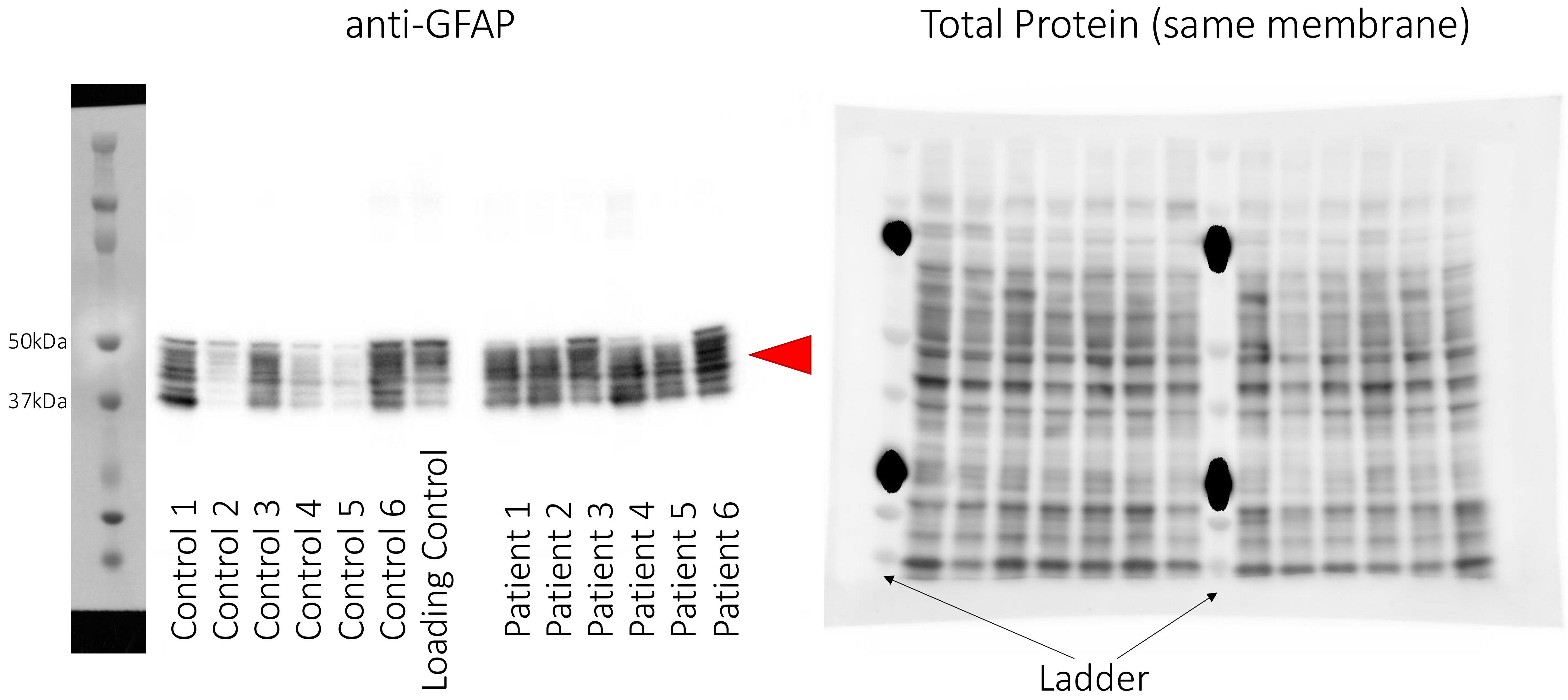

Striatum batch 1: 1-6

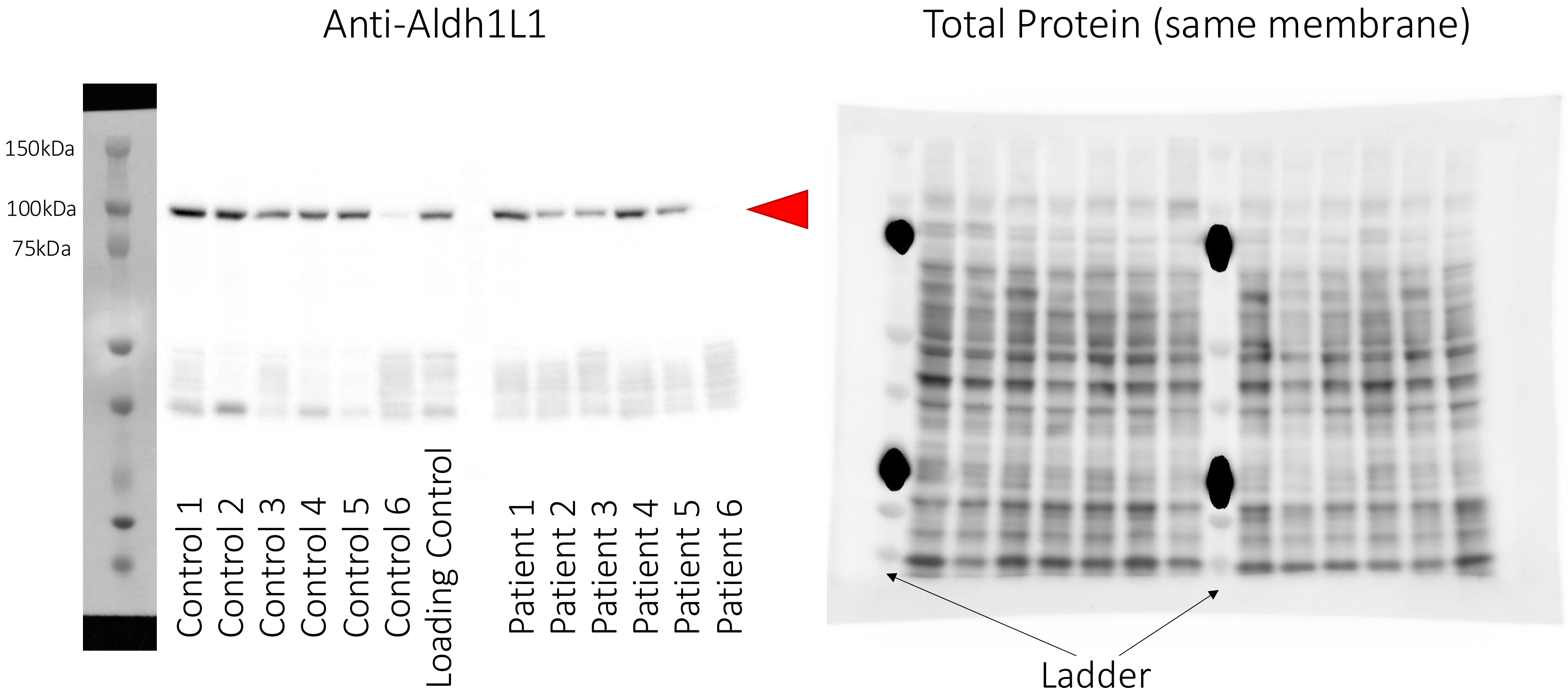

Midbrain SN batch 1: 1-6

anti-Cx43

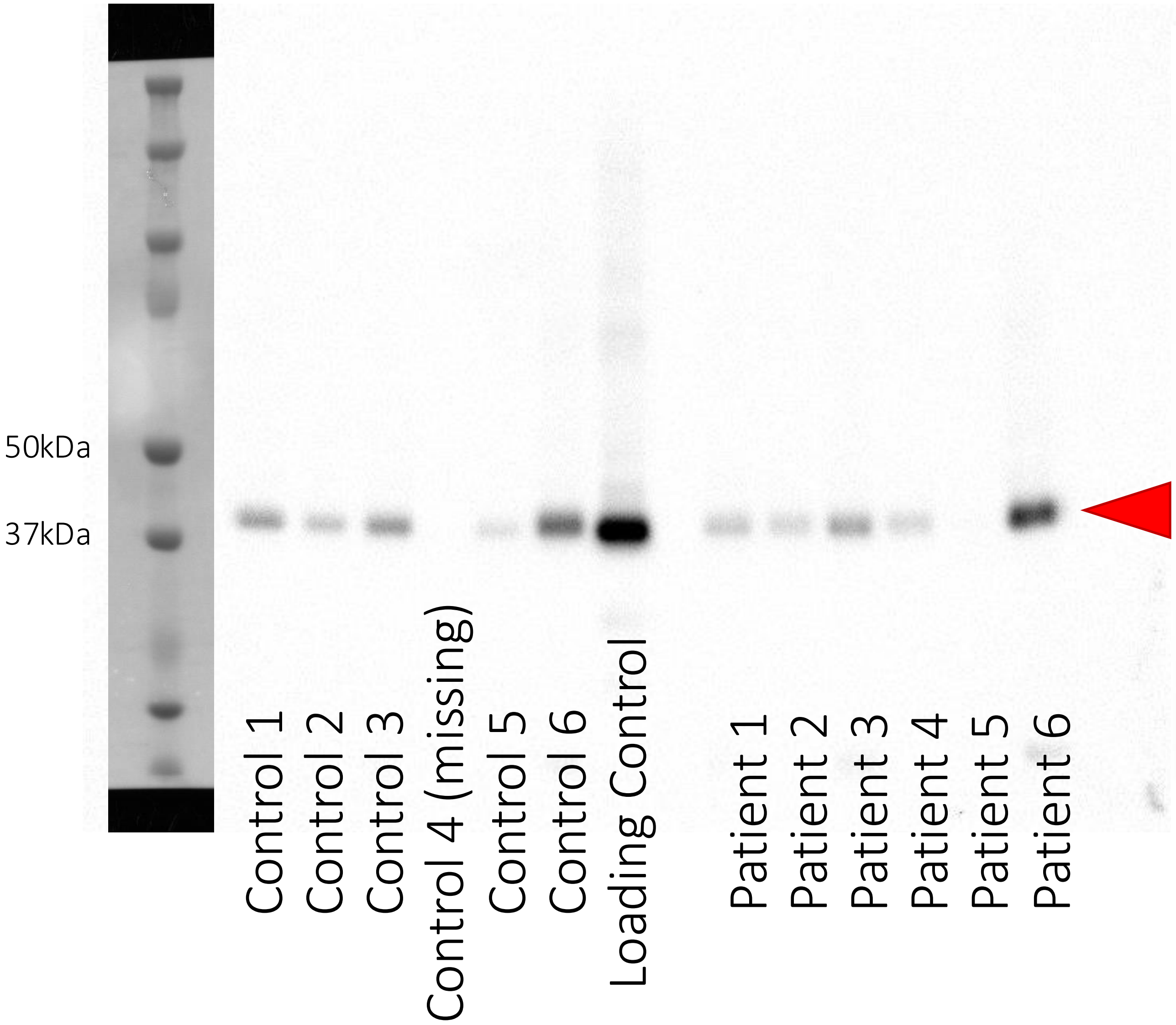

Total Protein (same membrane)

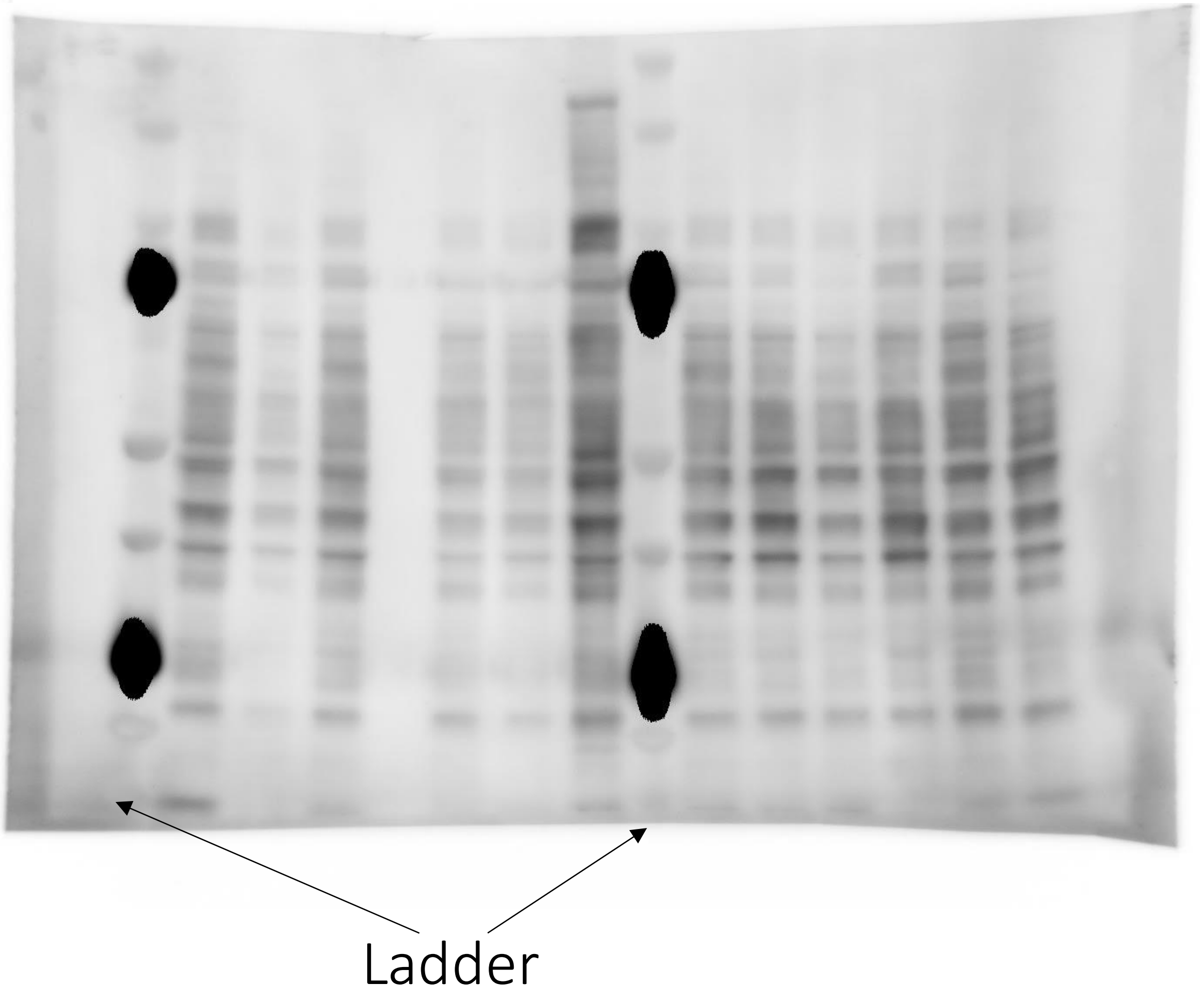

Midbrain SN batch 1: 1-6

anti-GFAP

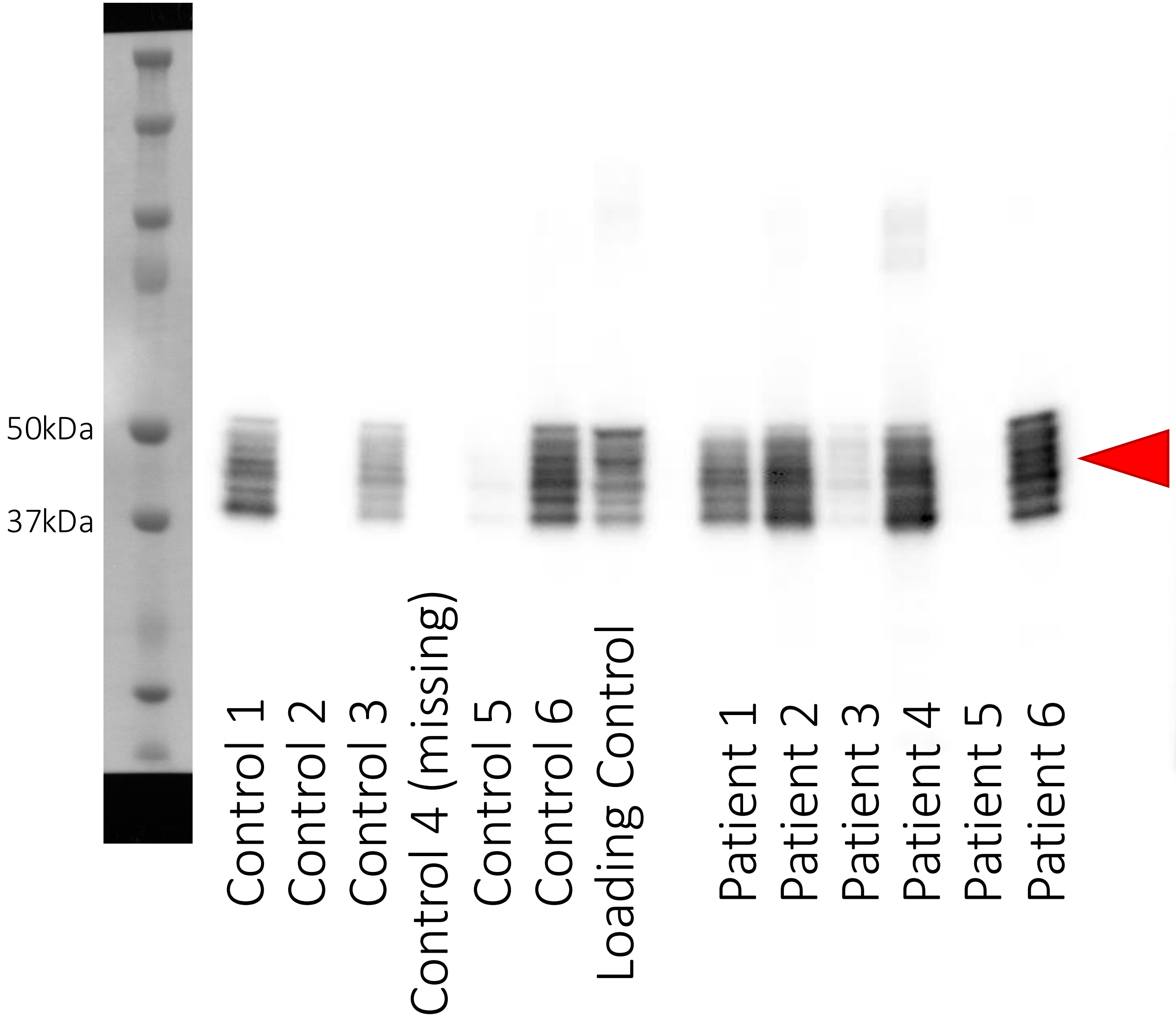

Total Protein (same membrane)

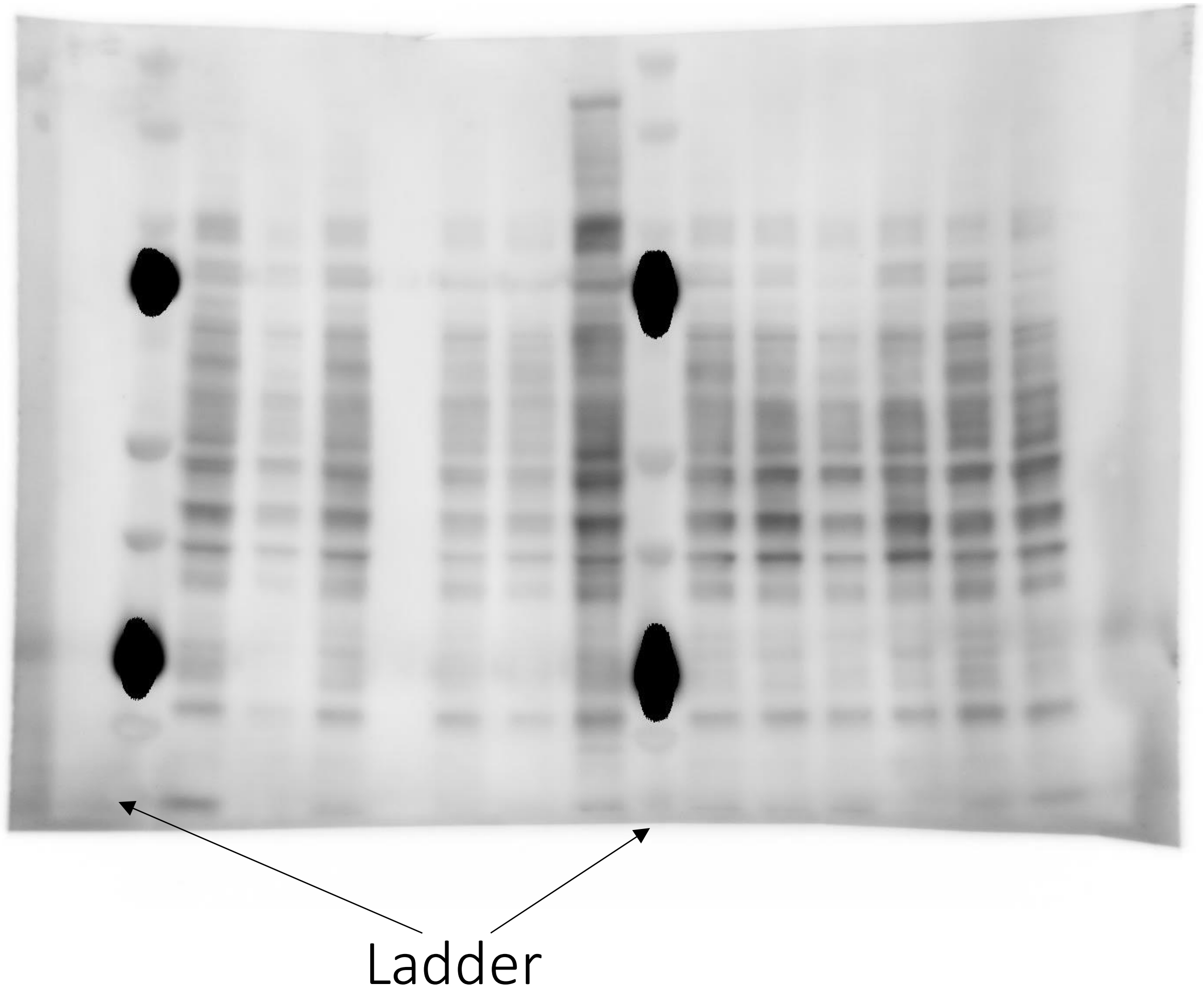

Midbrain SN batch 1: 1-6

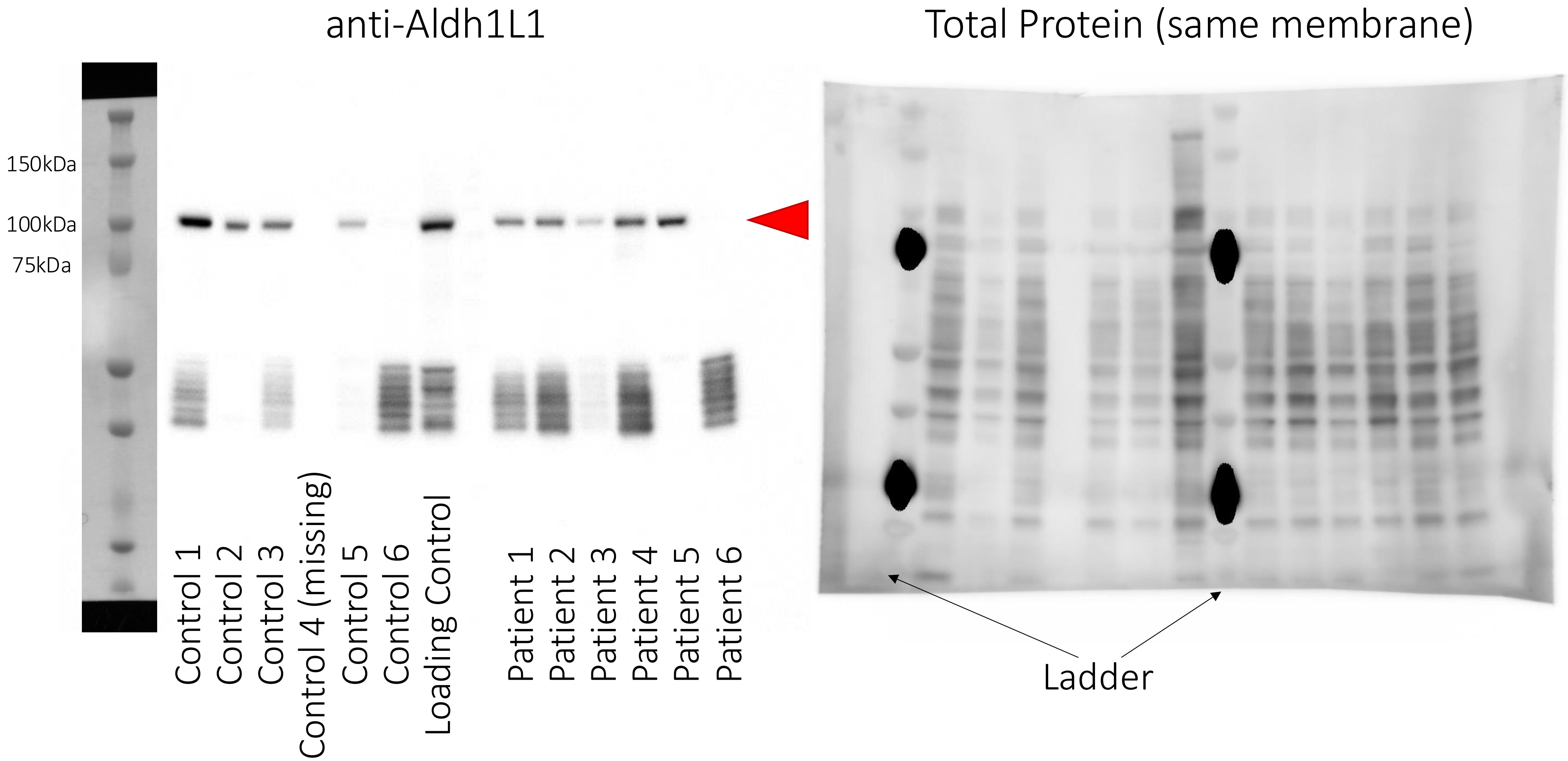

Parietal cortex batch 2: 7-12

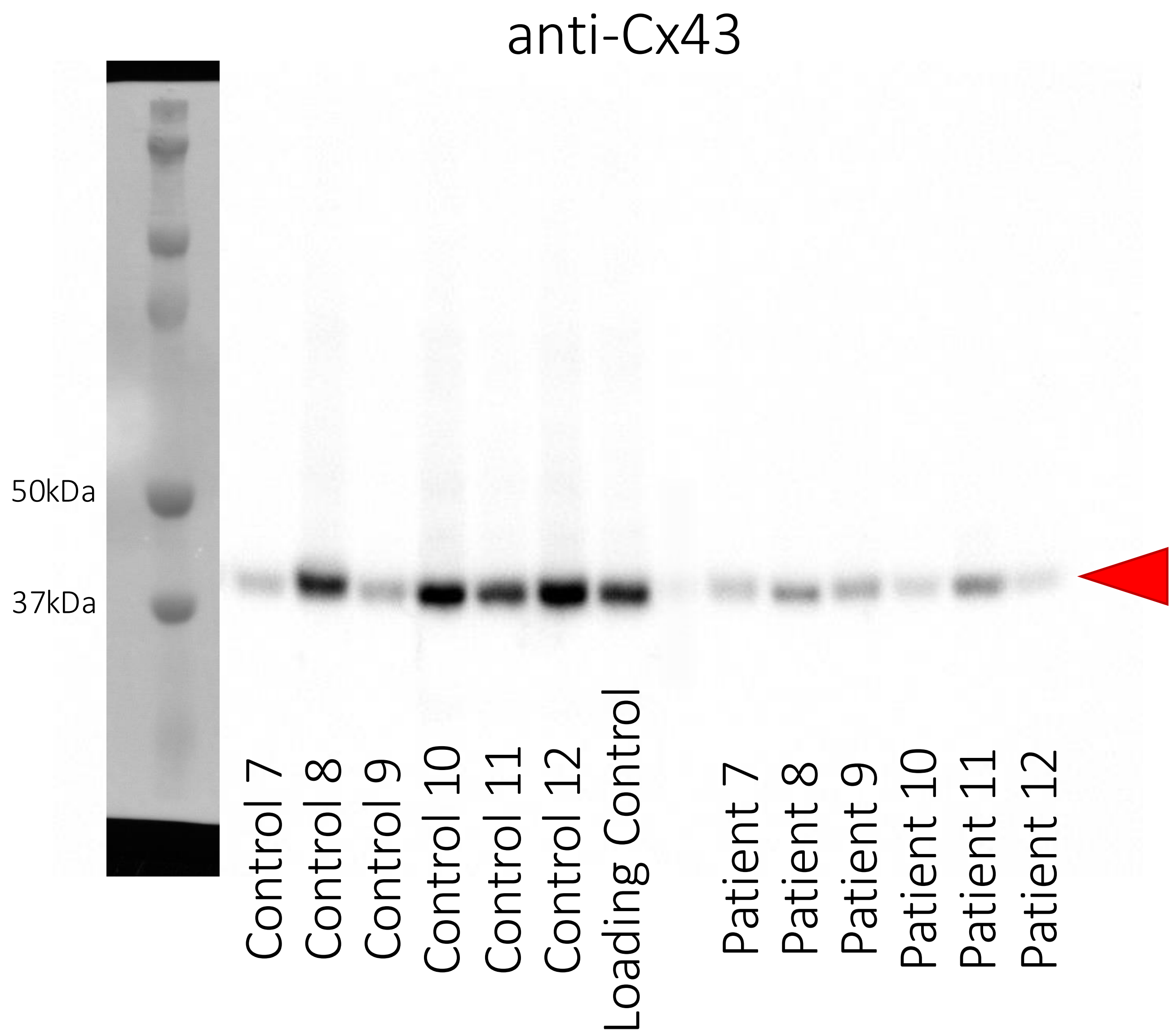

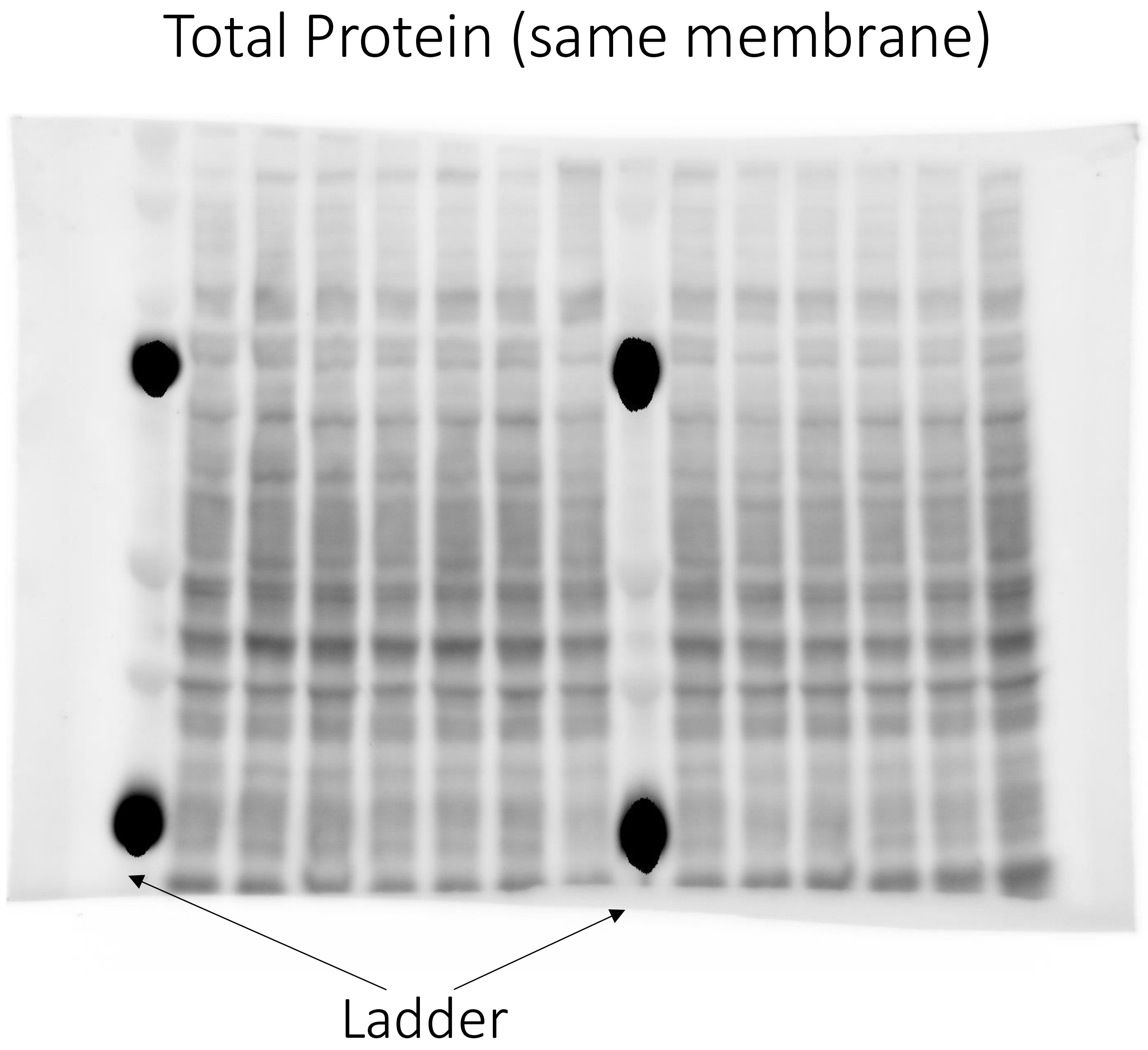

Parietal cortex batch 2: 7-12

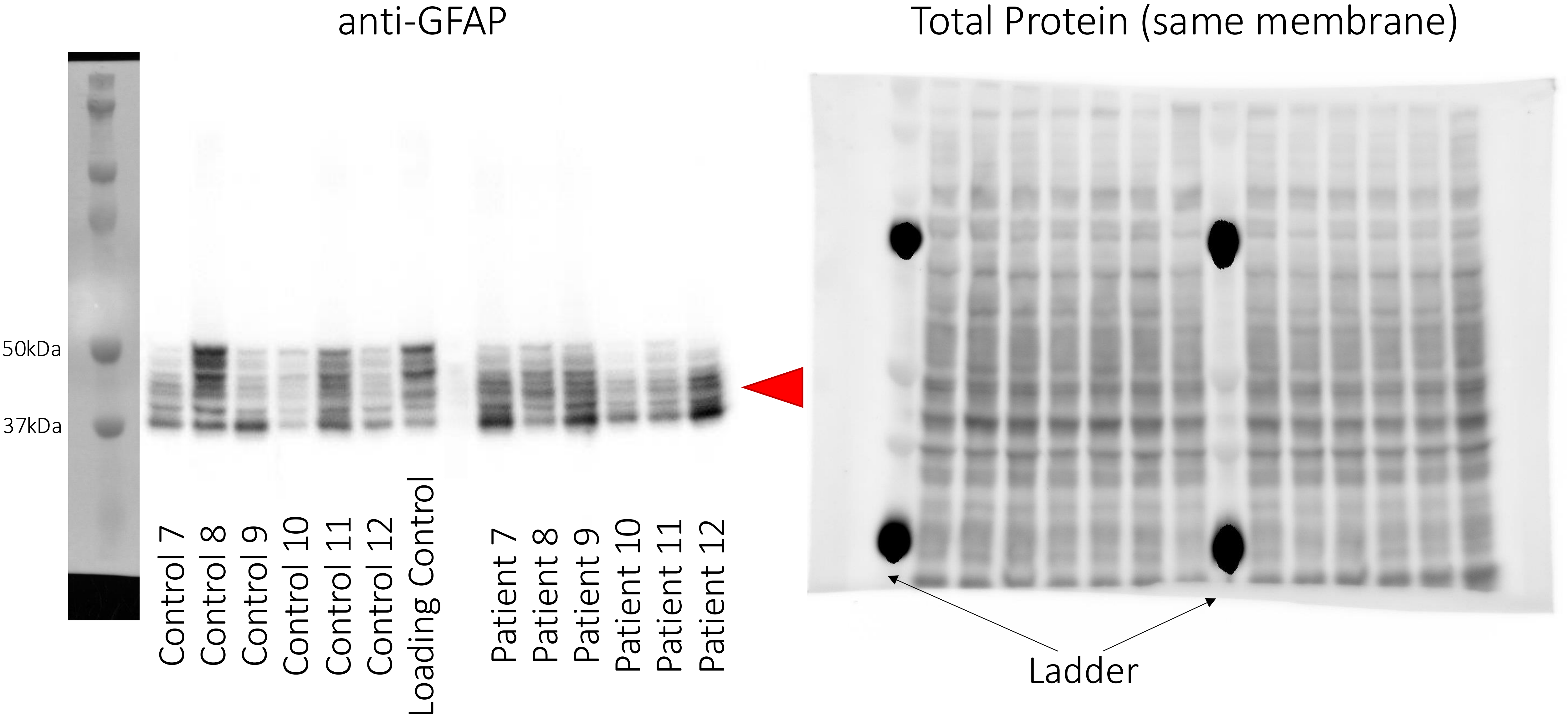

Parietal cortex batch 2: 7-12

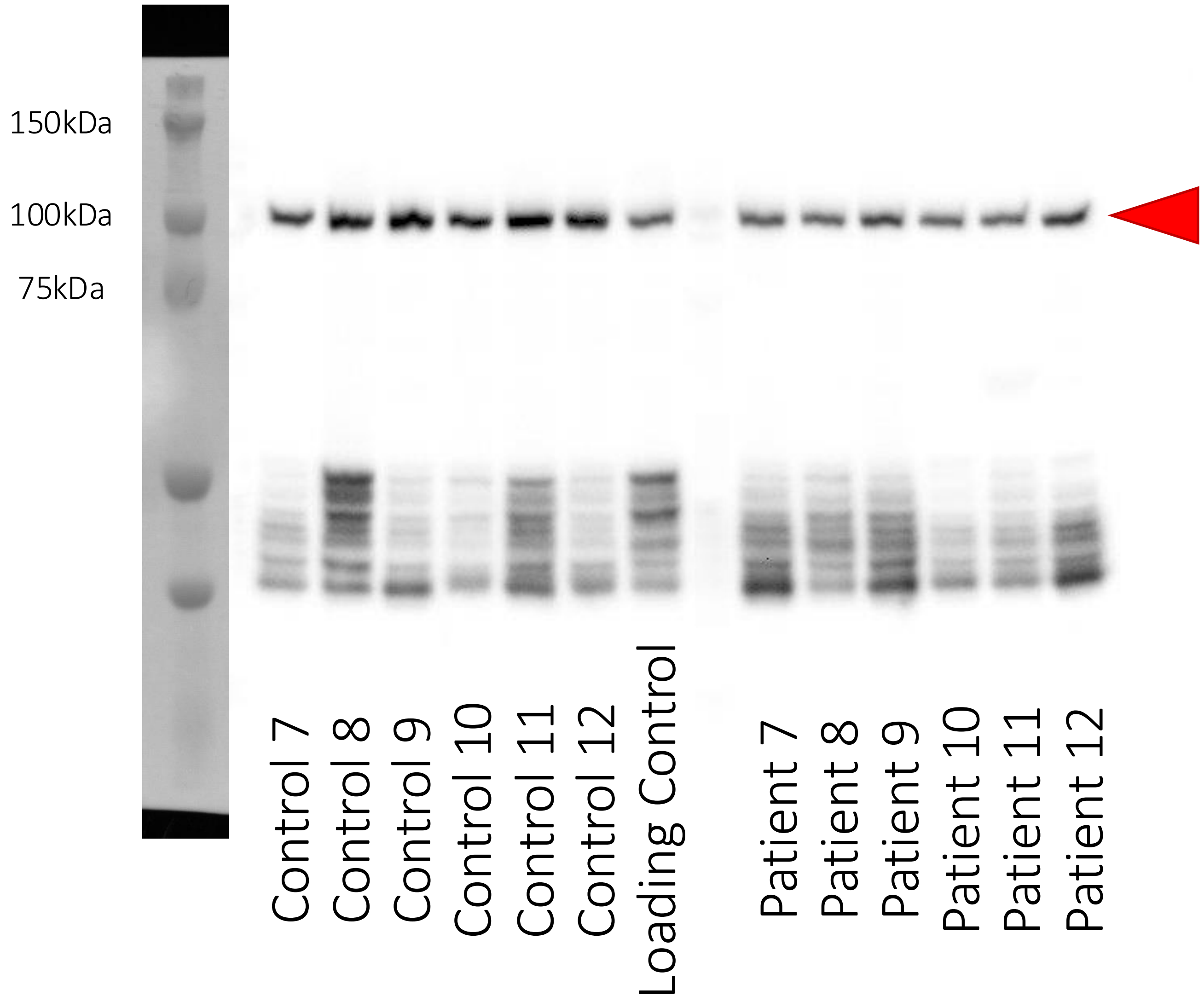

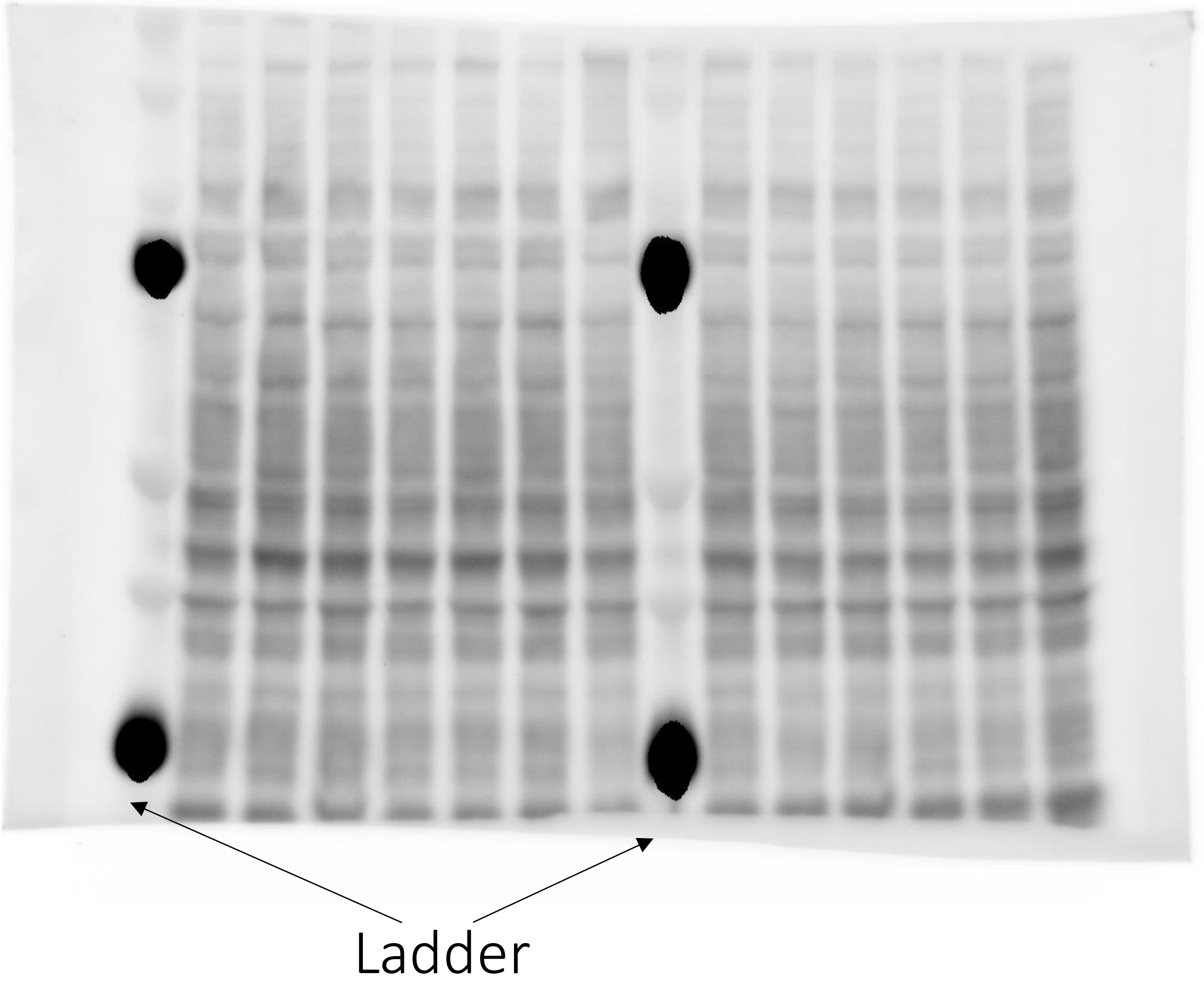

Striatum batch 2: 7-12

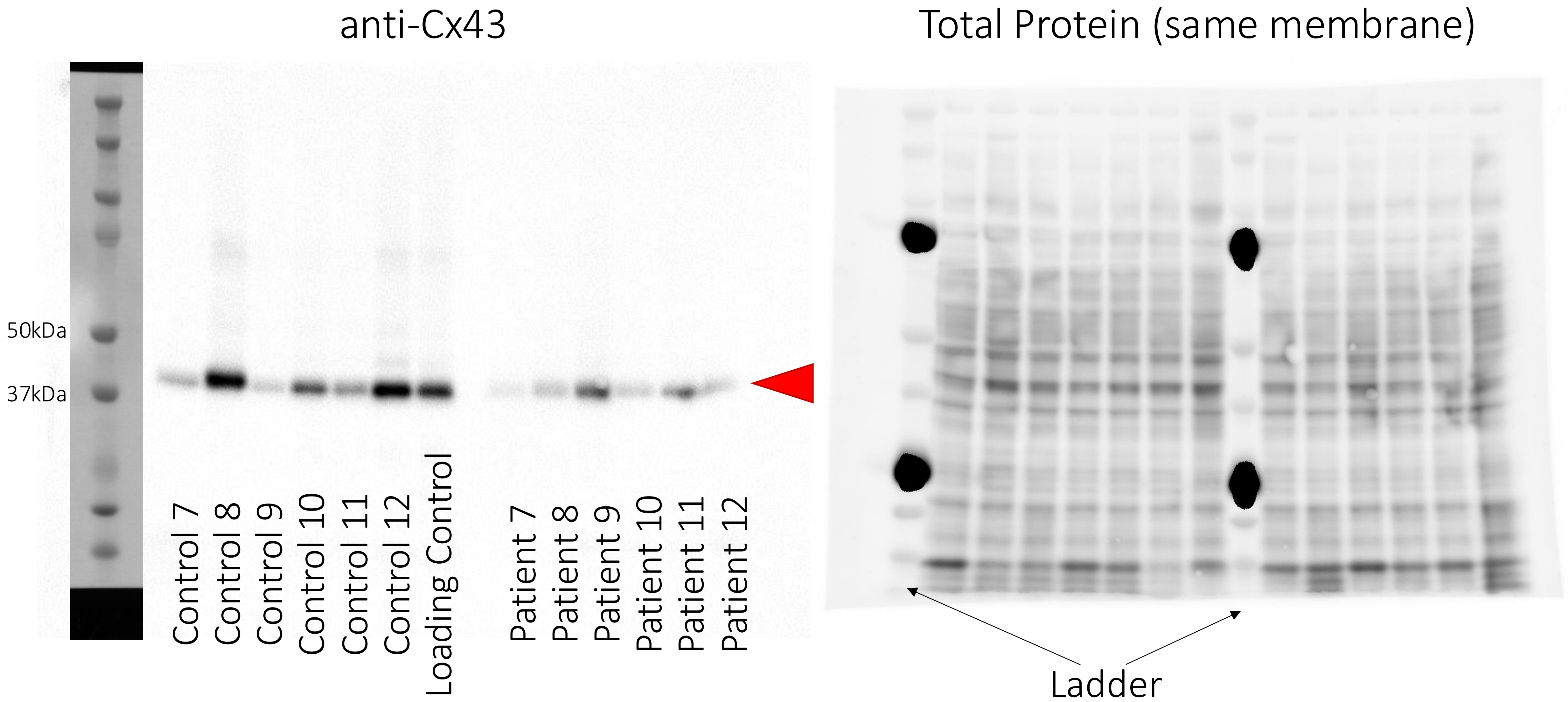

Striatum batch 2: 7-12

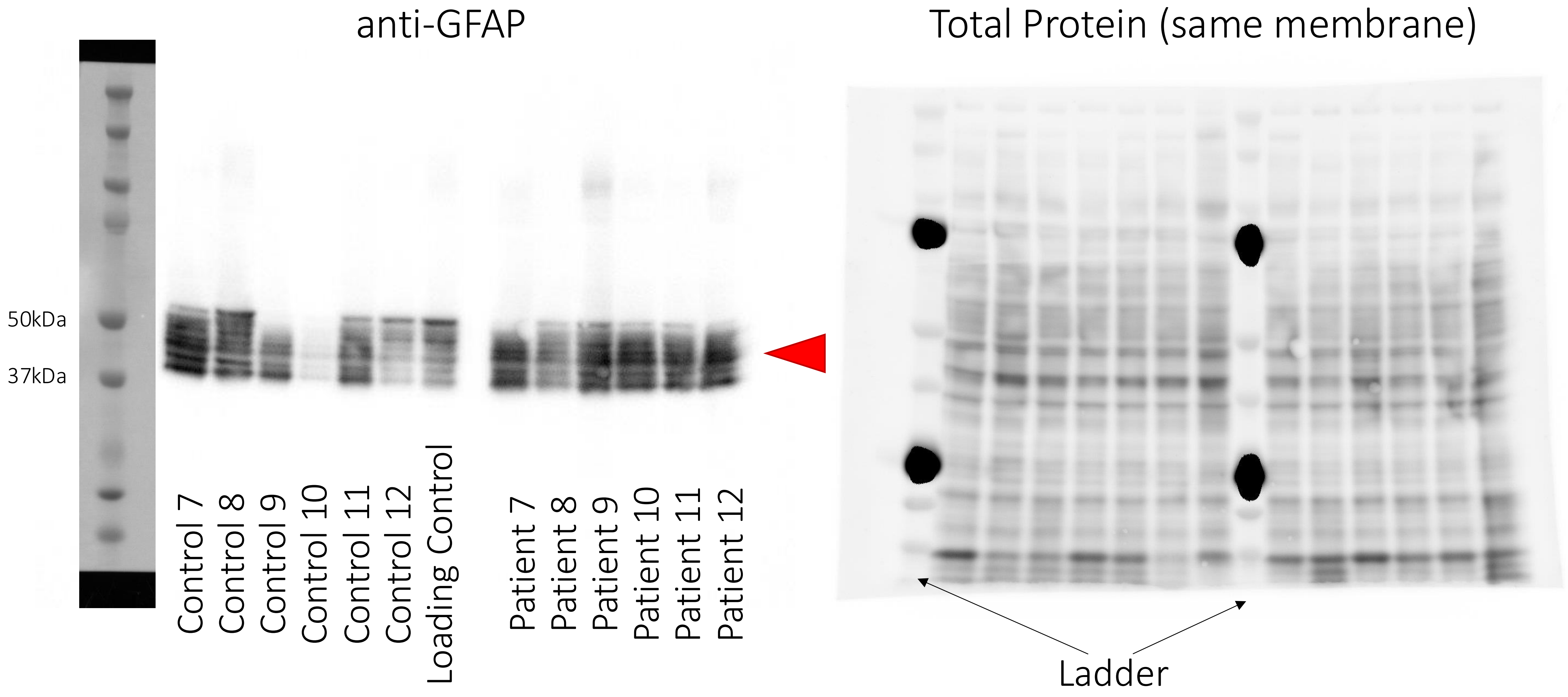

Striatum batch 2: 7-12

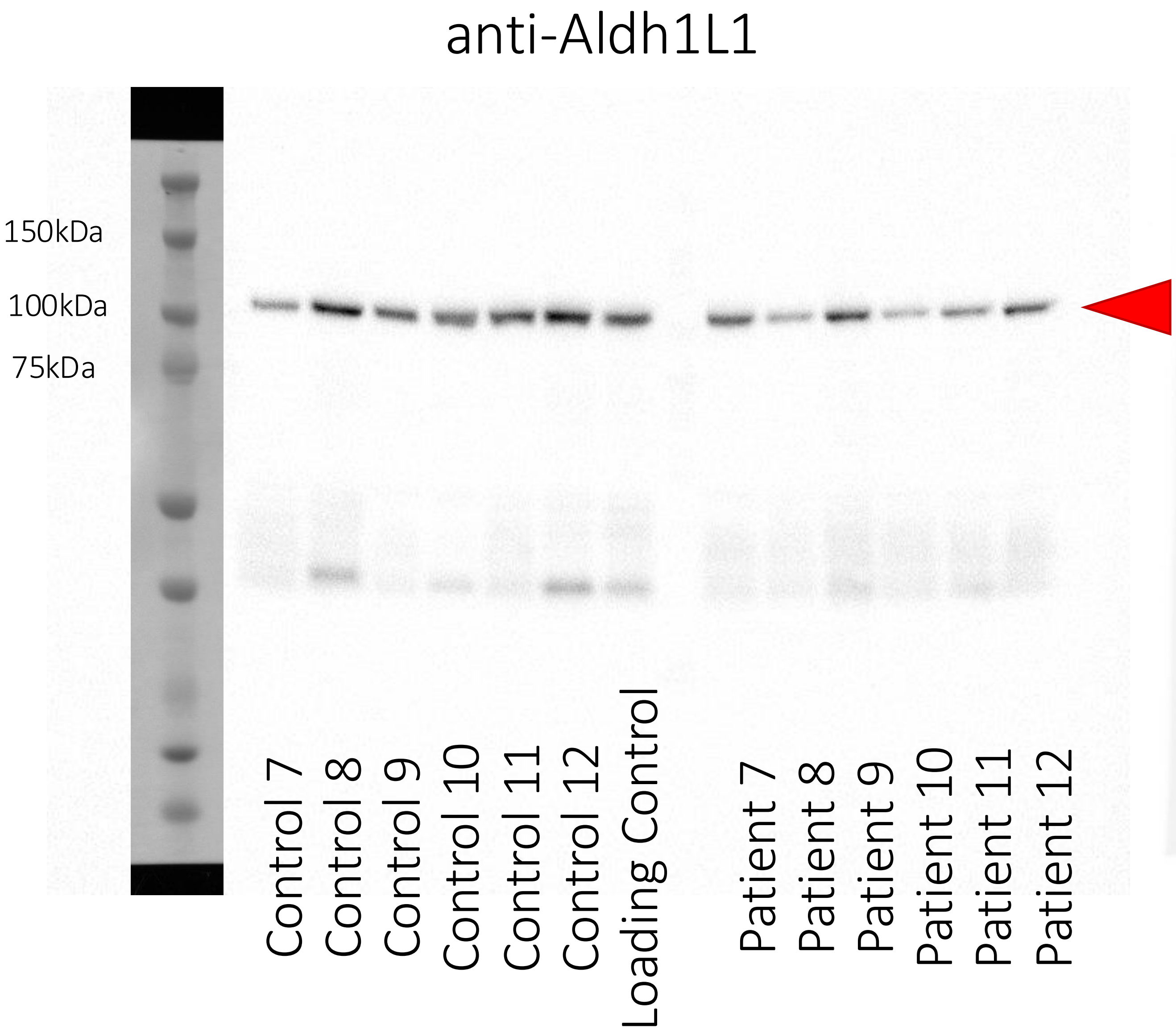

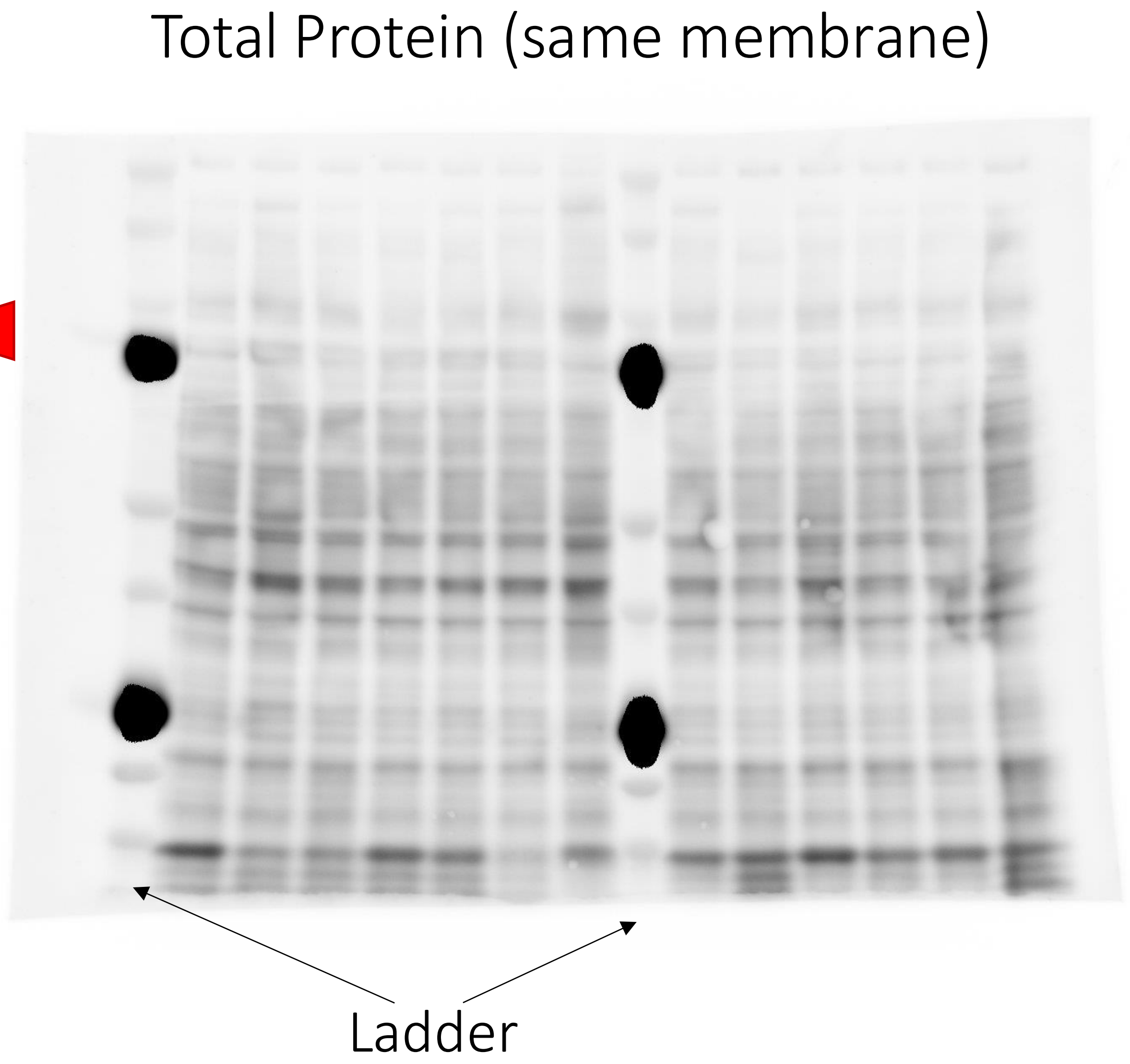

Midbrain SN batch 2: 7-12

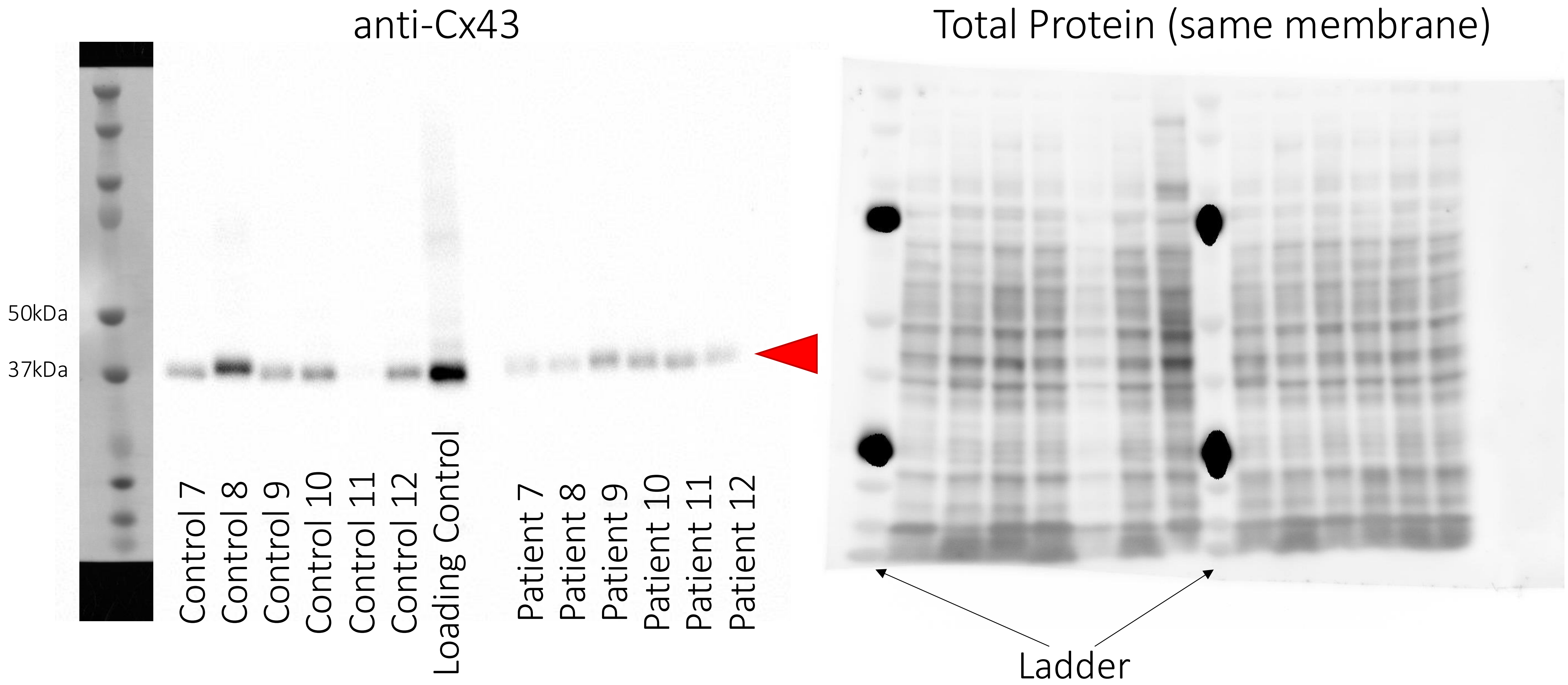

Midbrain SN batch 2: 7-12

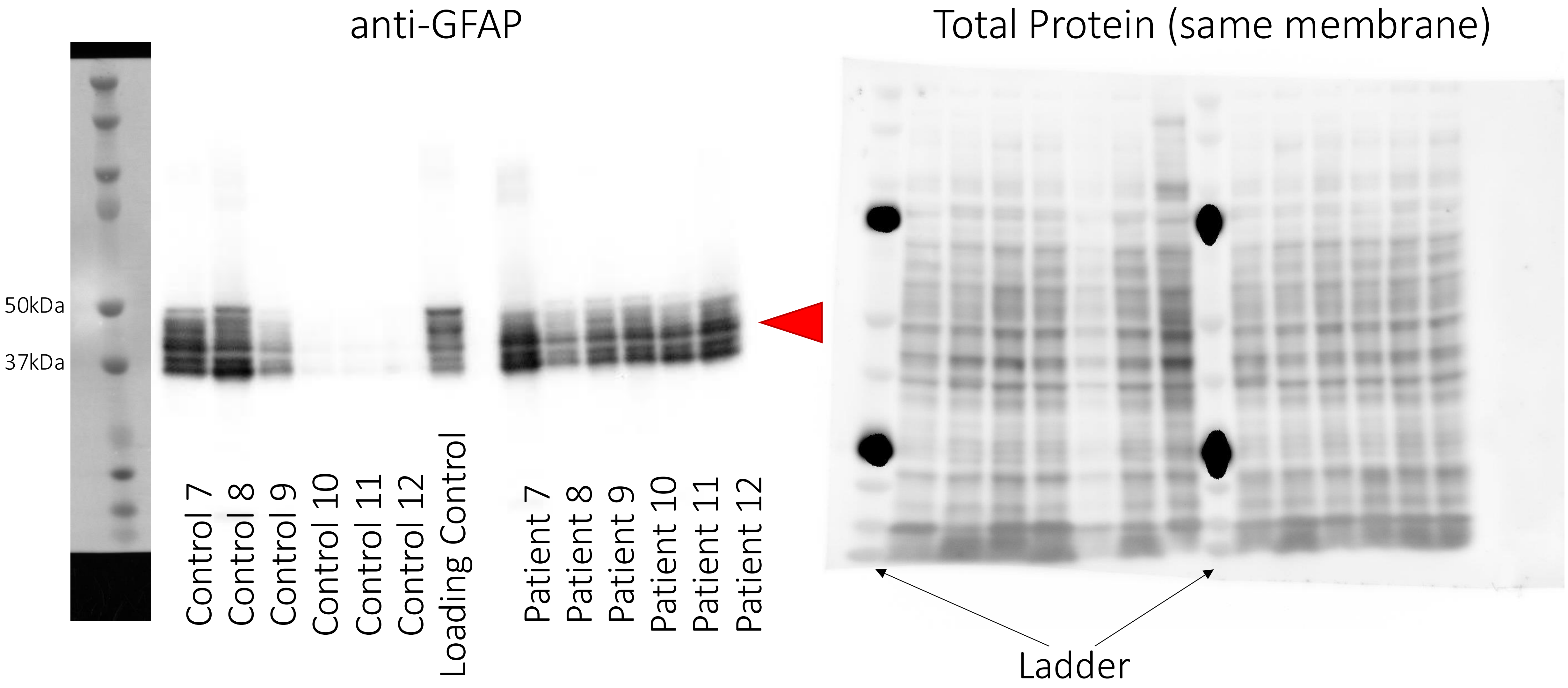

Midbrain SN batch 2: 7-12

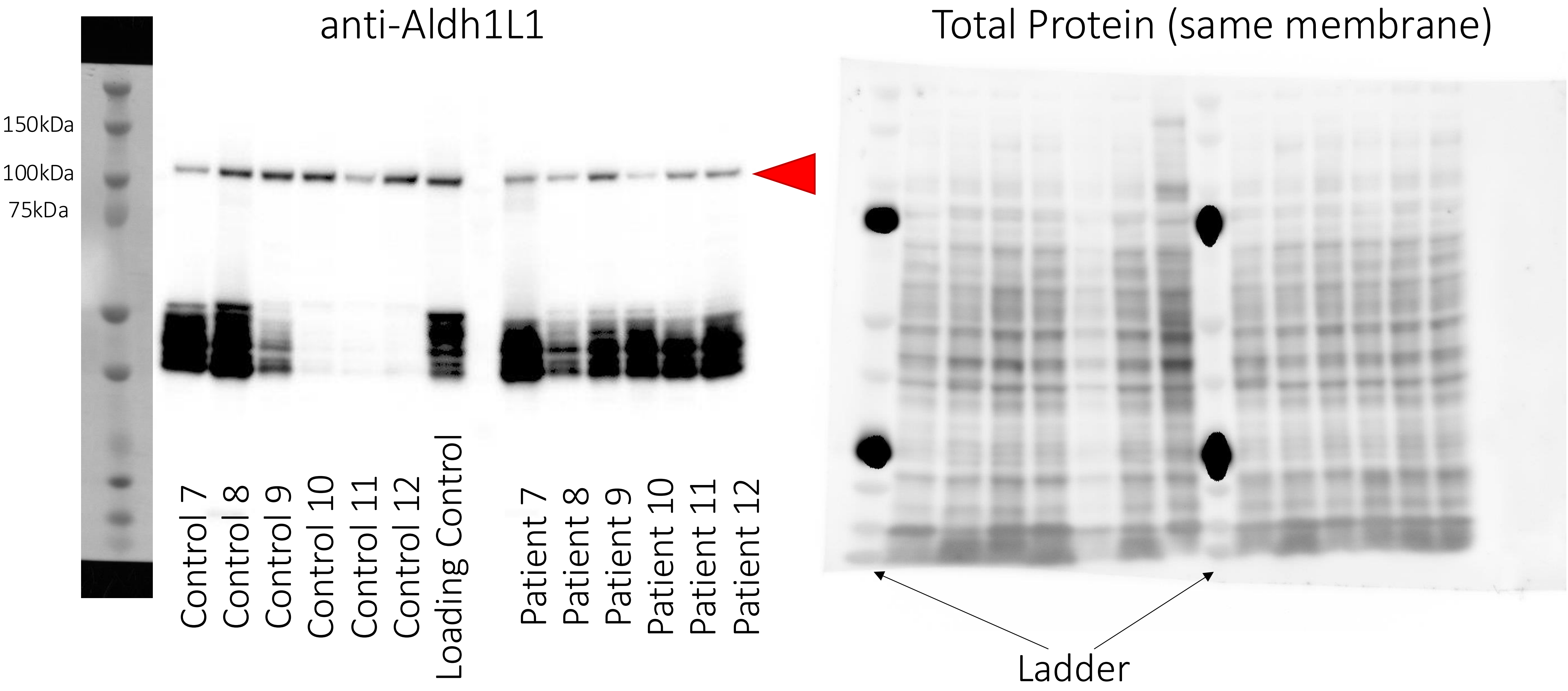

Parietal cortex batch 3: 13-18

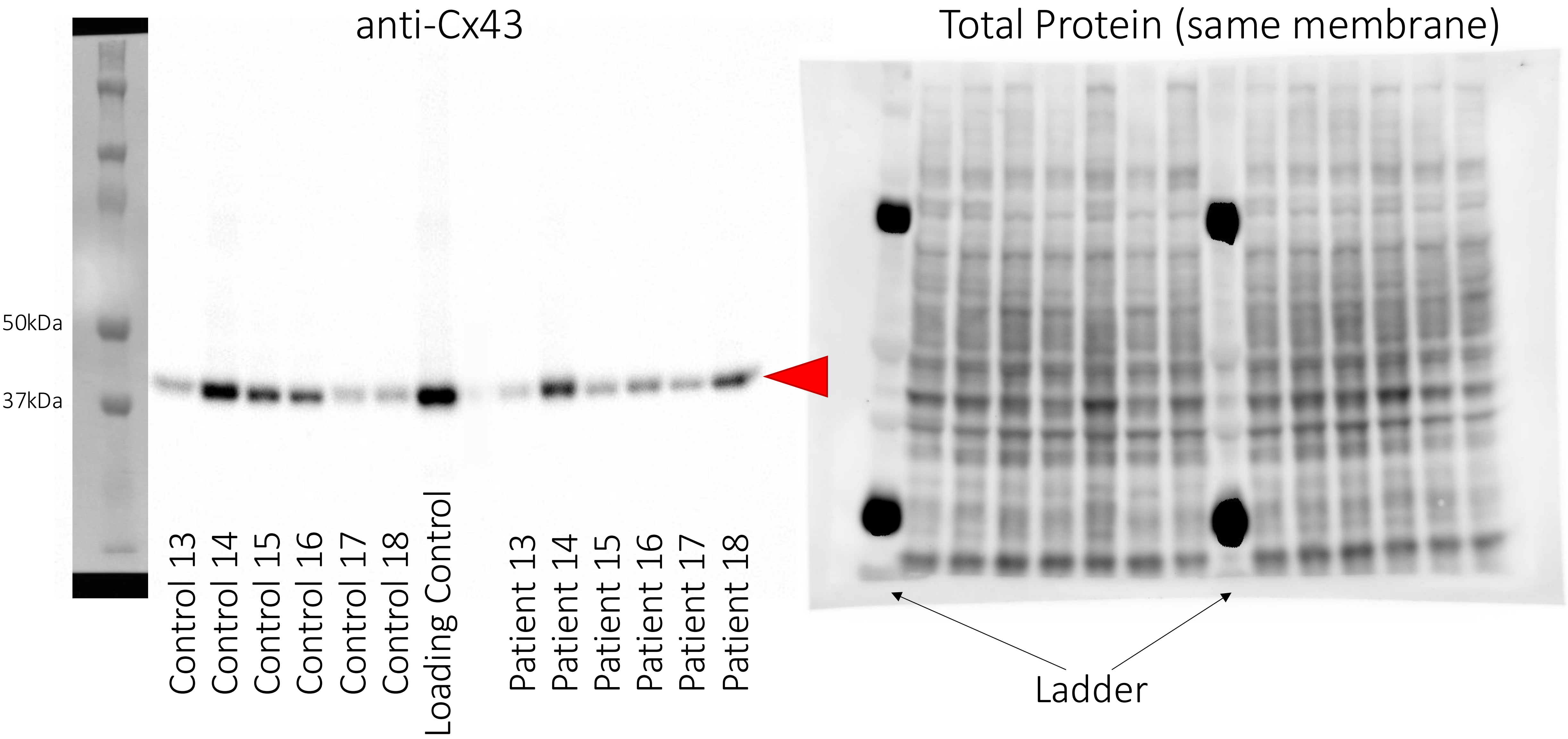

Parietal cortex batch 3: 13-18

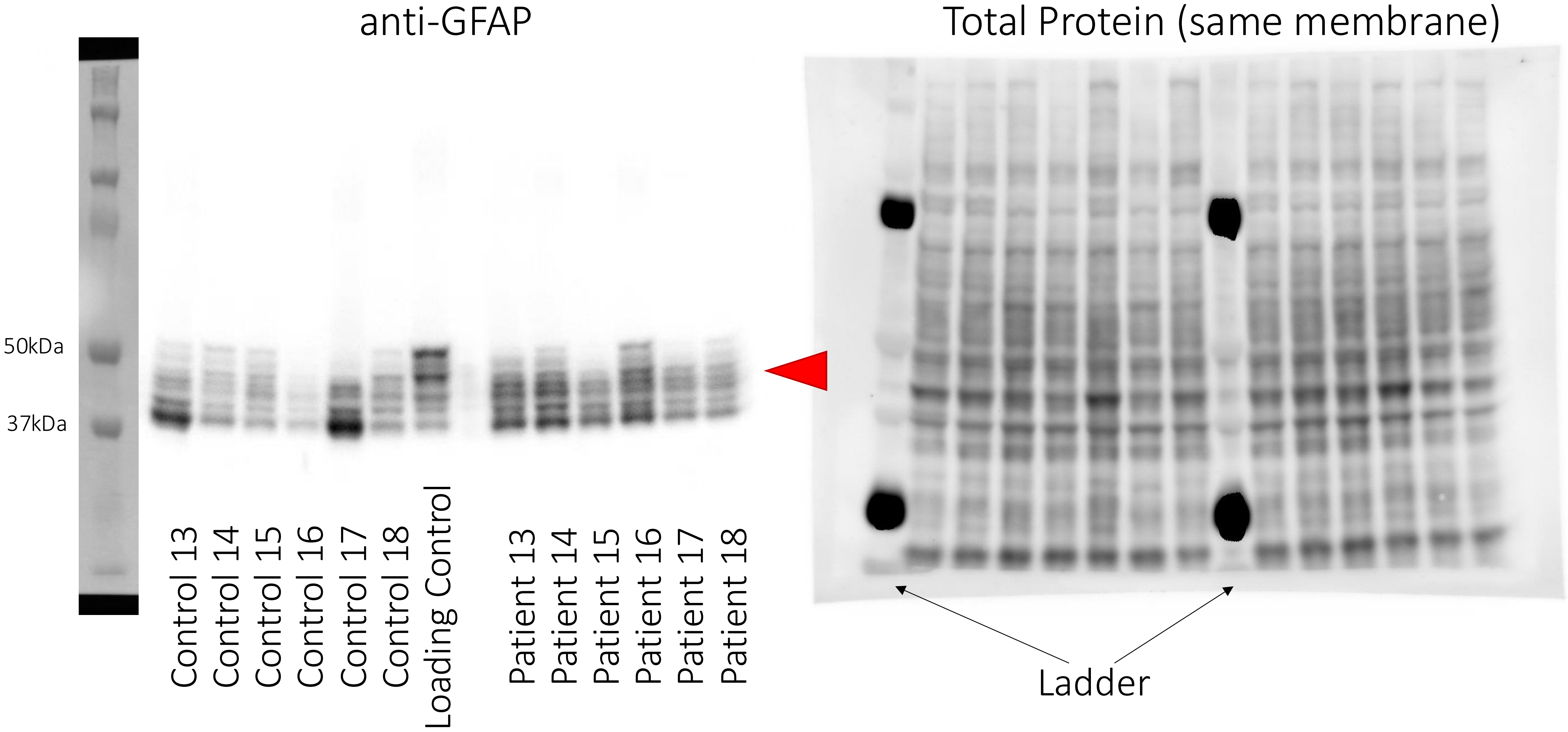

Parietal cortex batch 3: 13-18

Anti-Aldh1L1

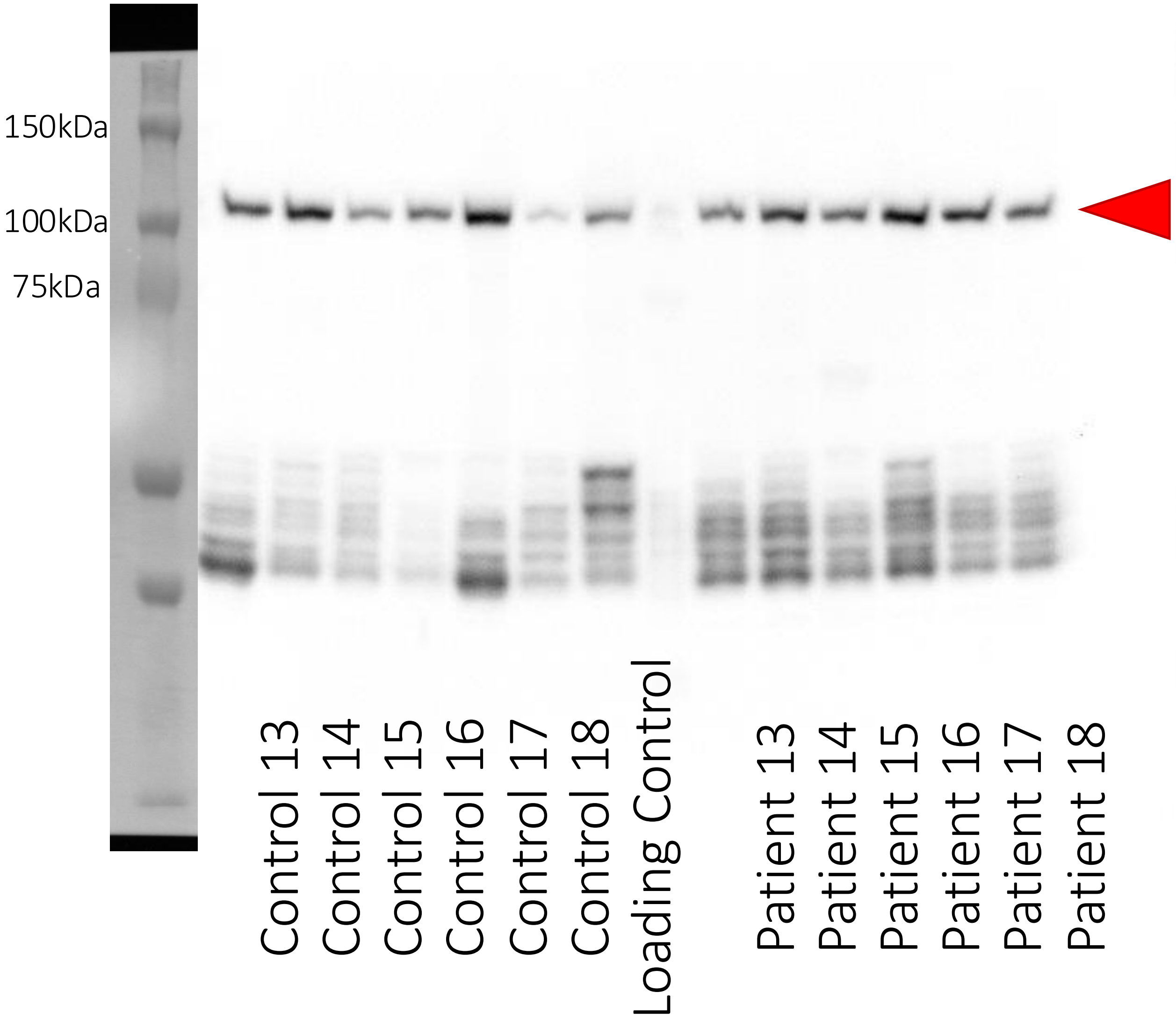

Total Protein (same membrane)

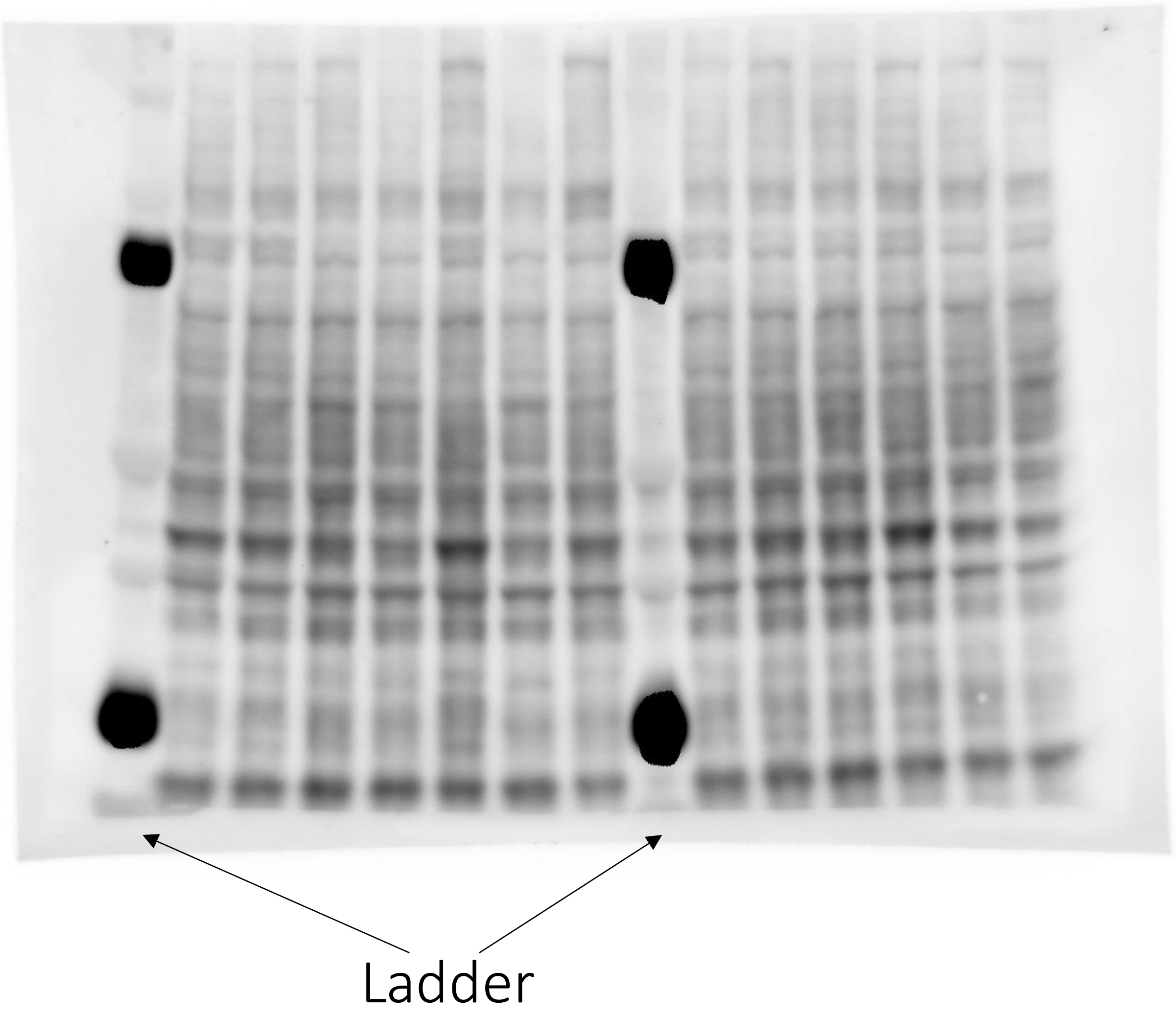

Striatum batch 3: 13-18

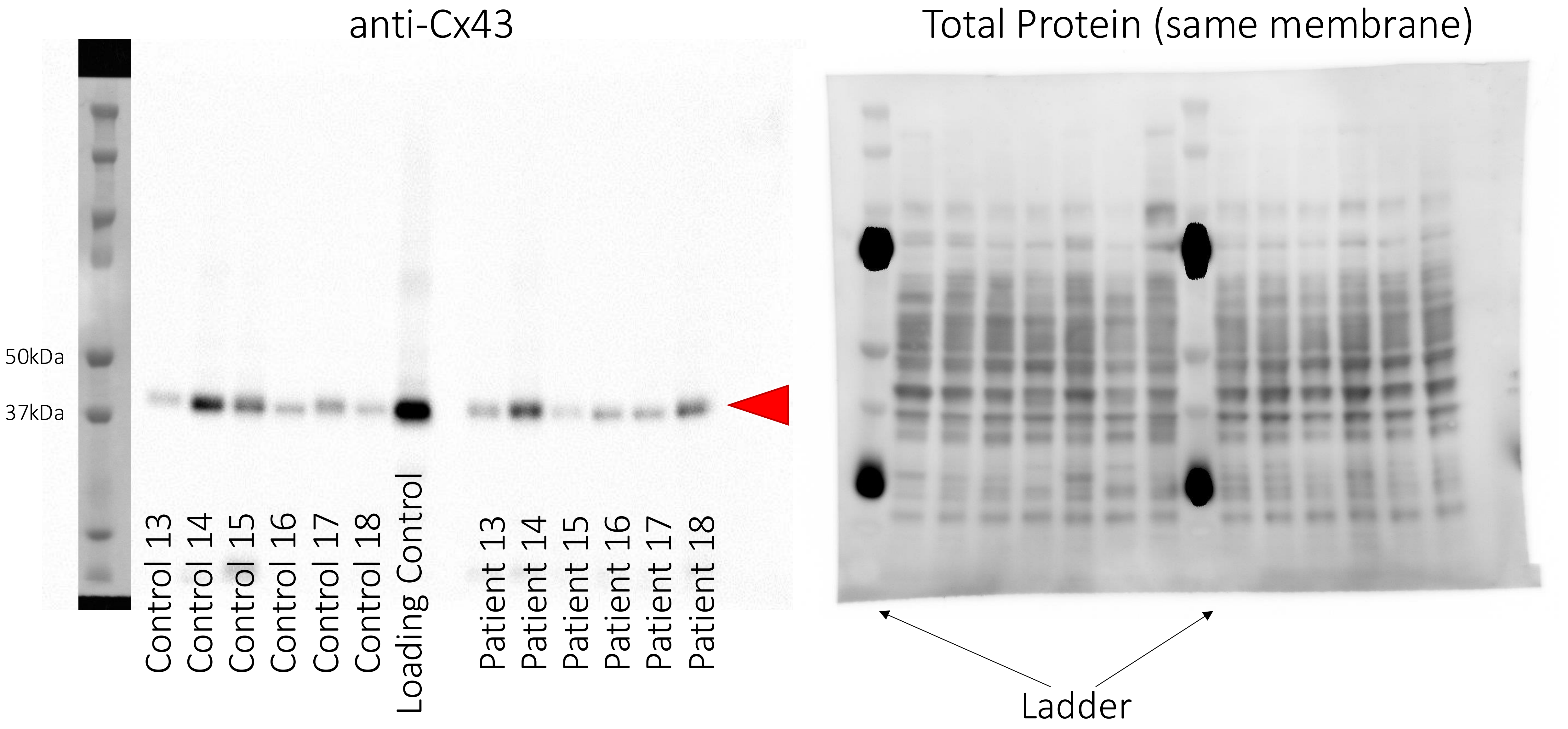

Striatum batch 3: 13-18

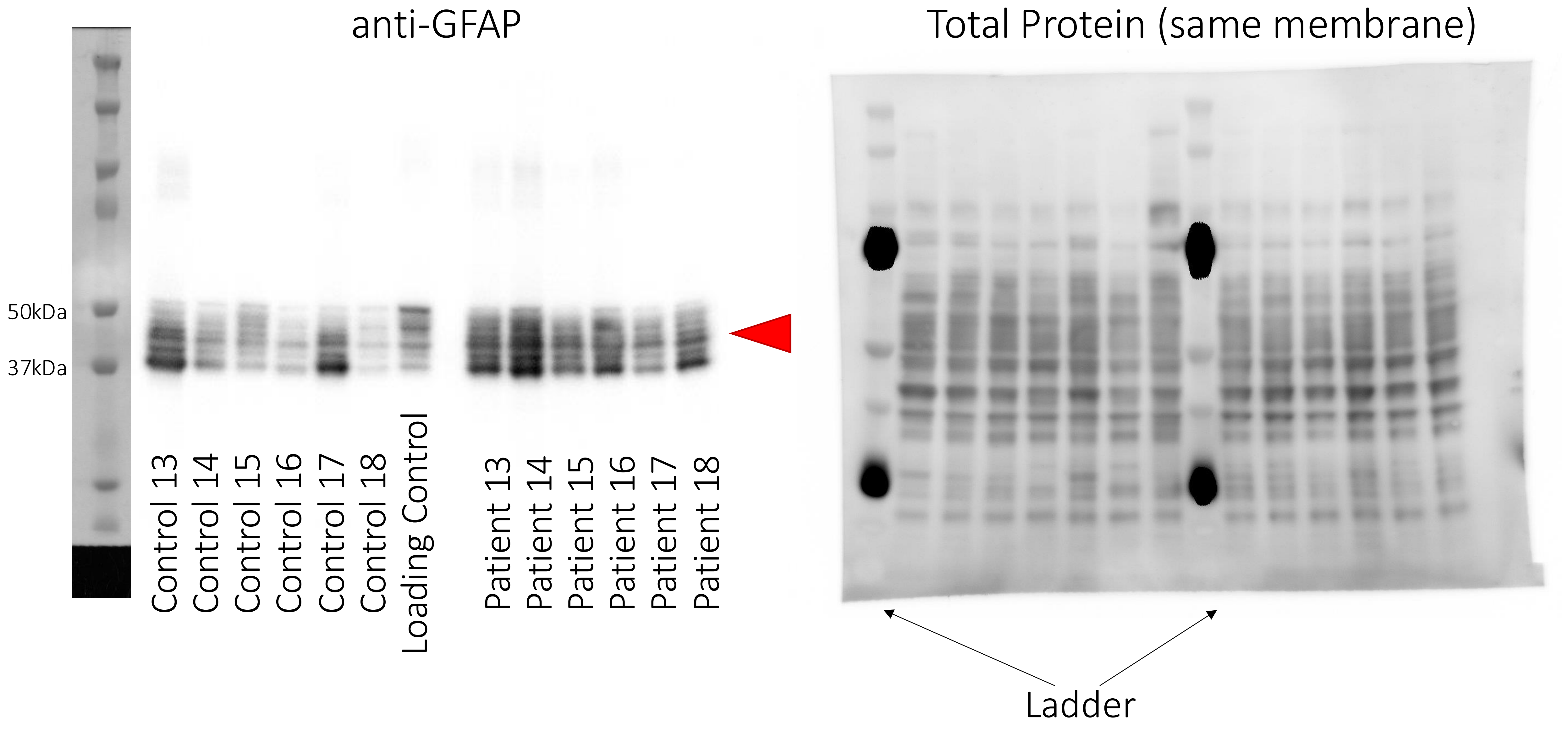

Striatum batch 3: 13-18

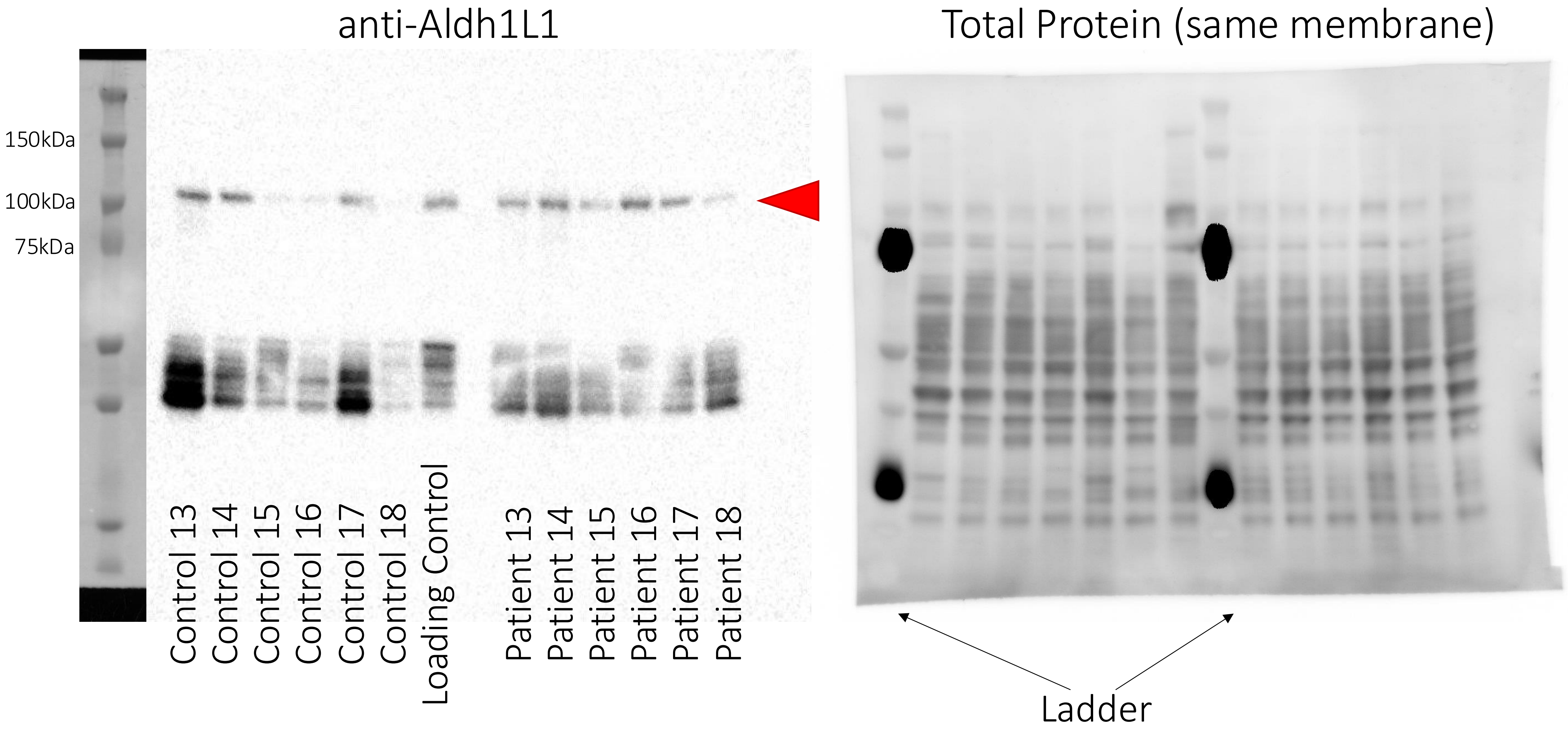

Midbrain SN batch 3: 13-18

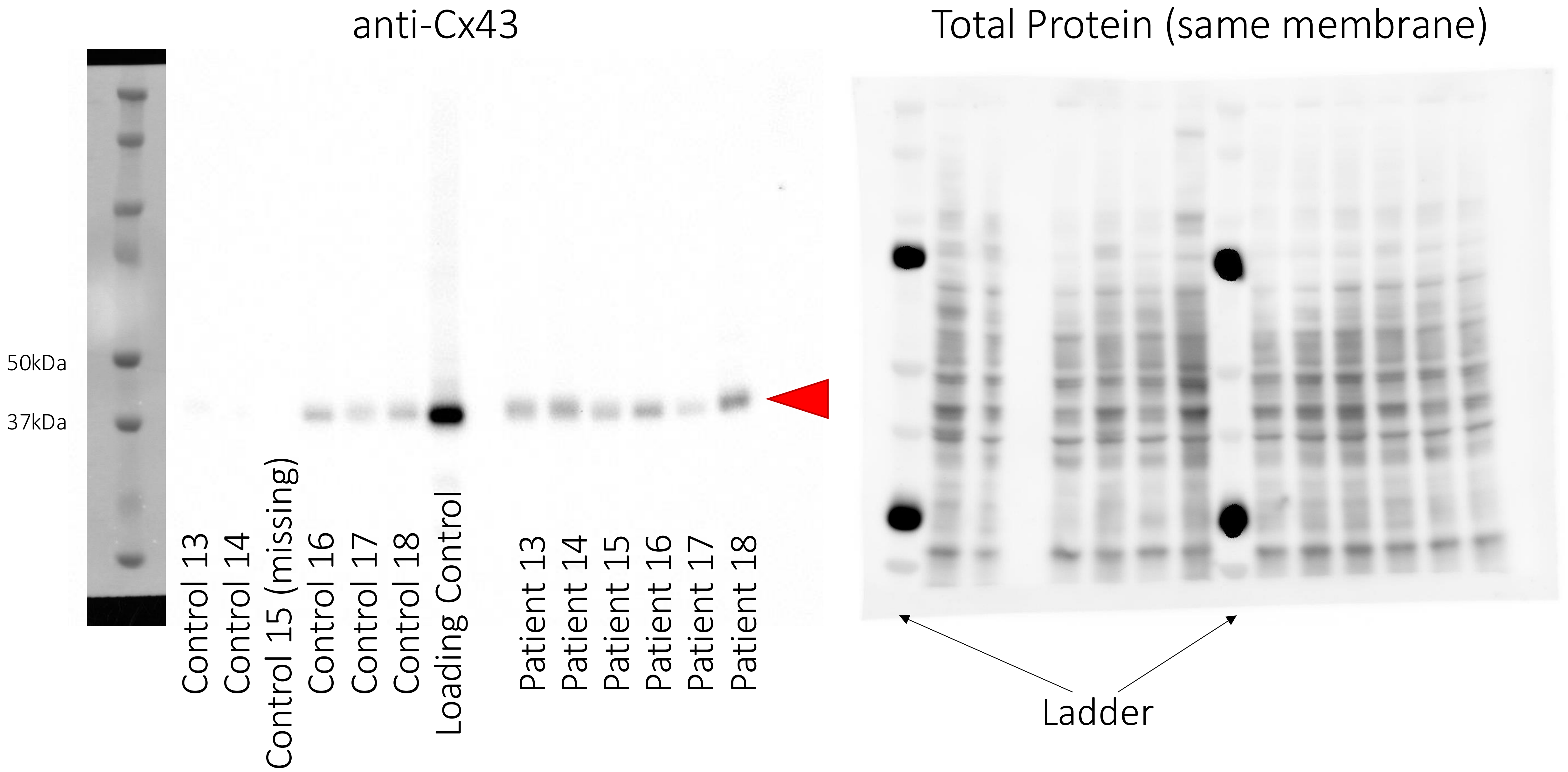

Midbrain SN batch 3: 13-18

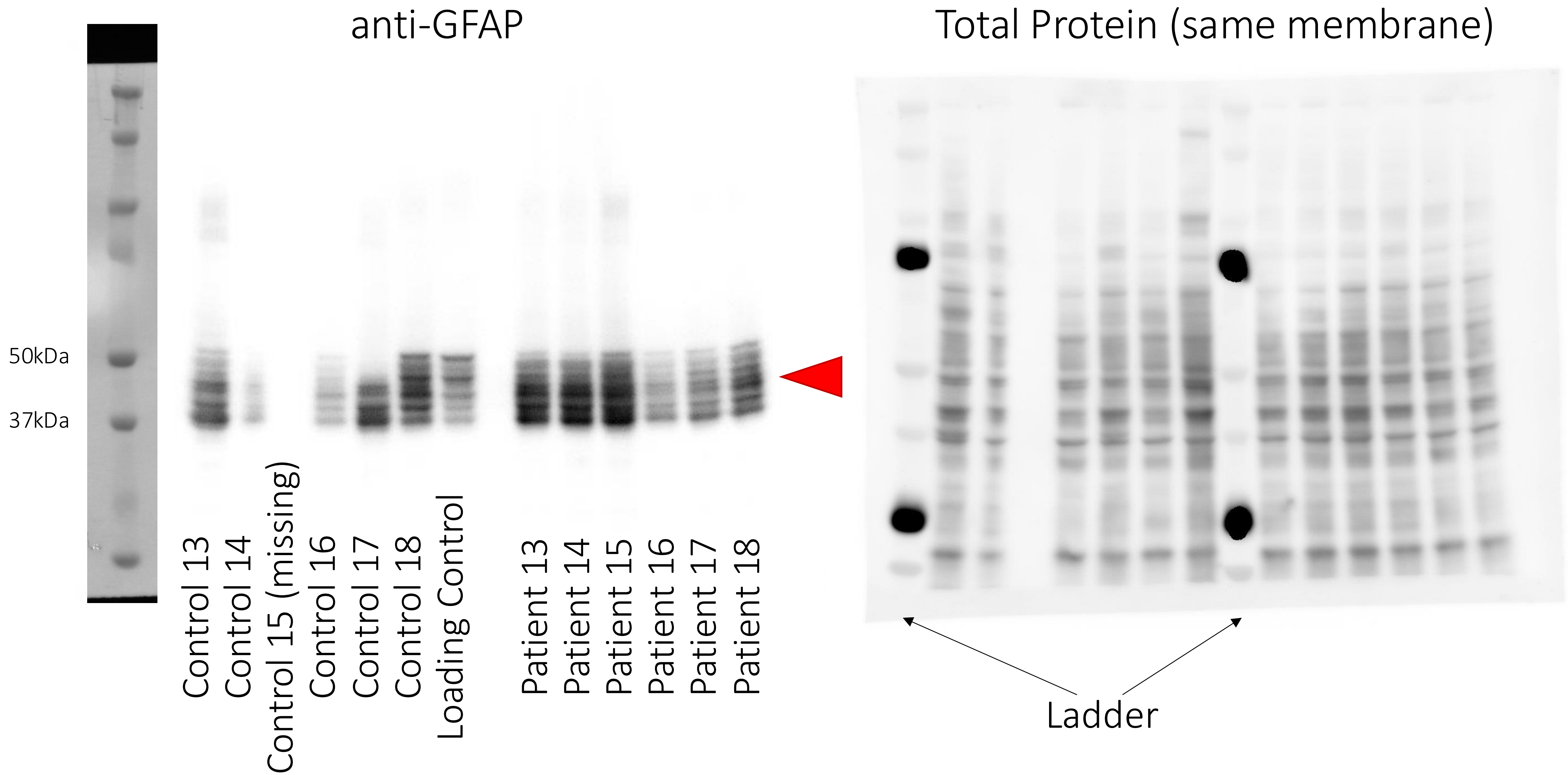

Midbrain SN batch 3: 13-18

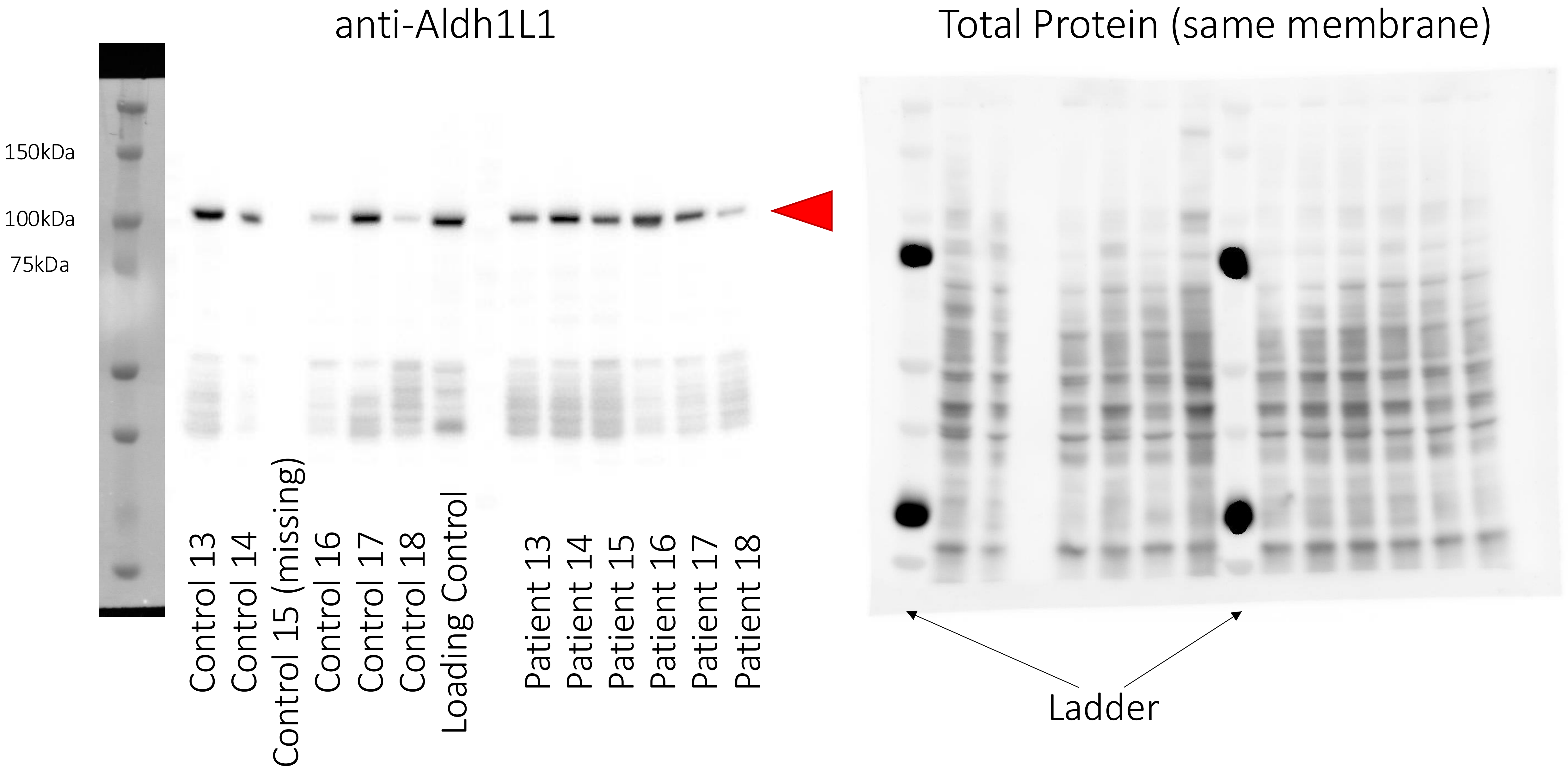

All regions batch 4: 19-20

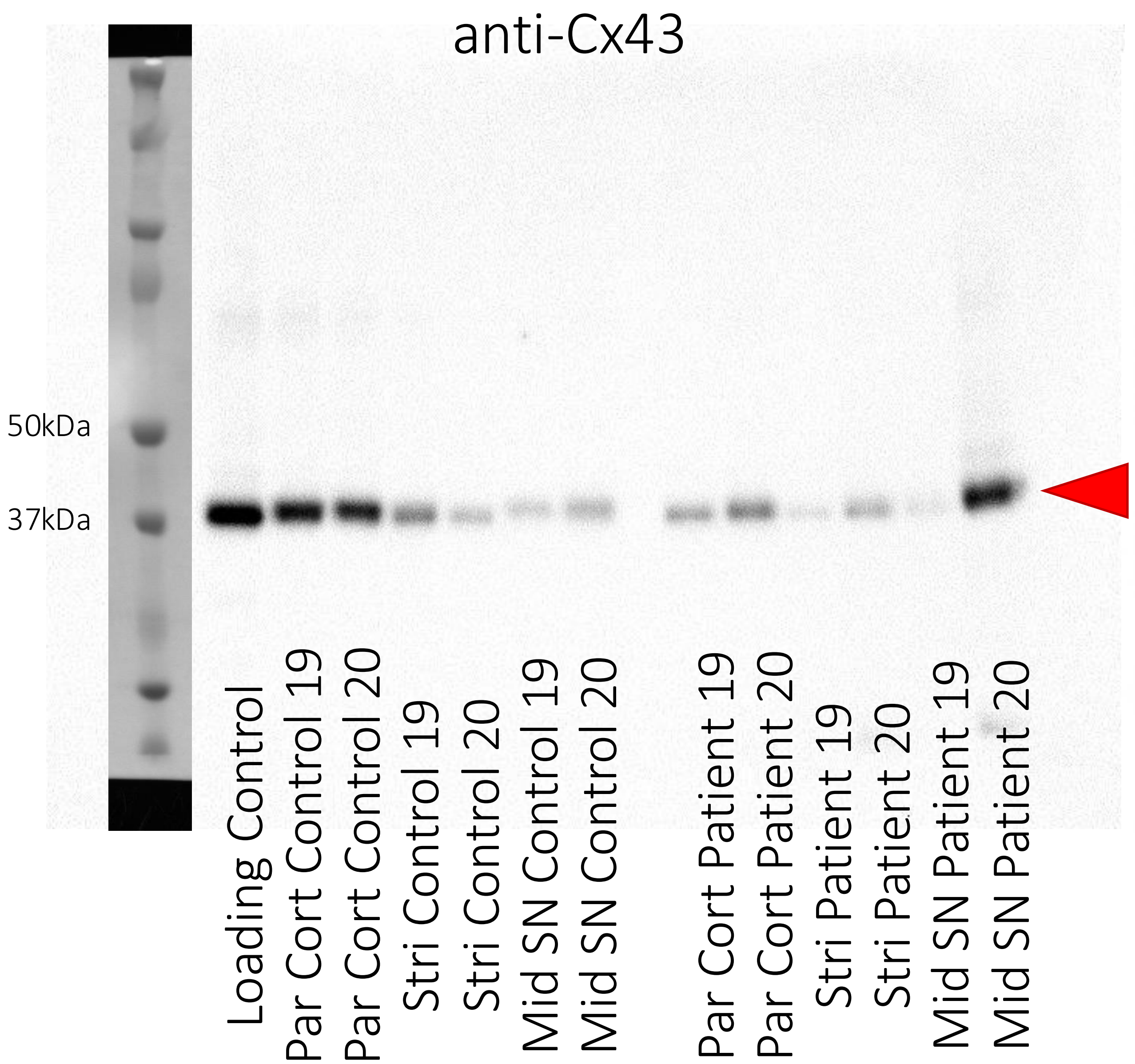

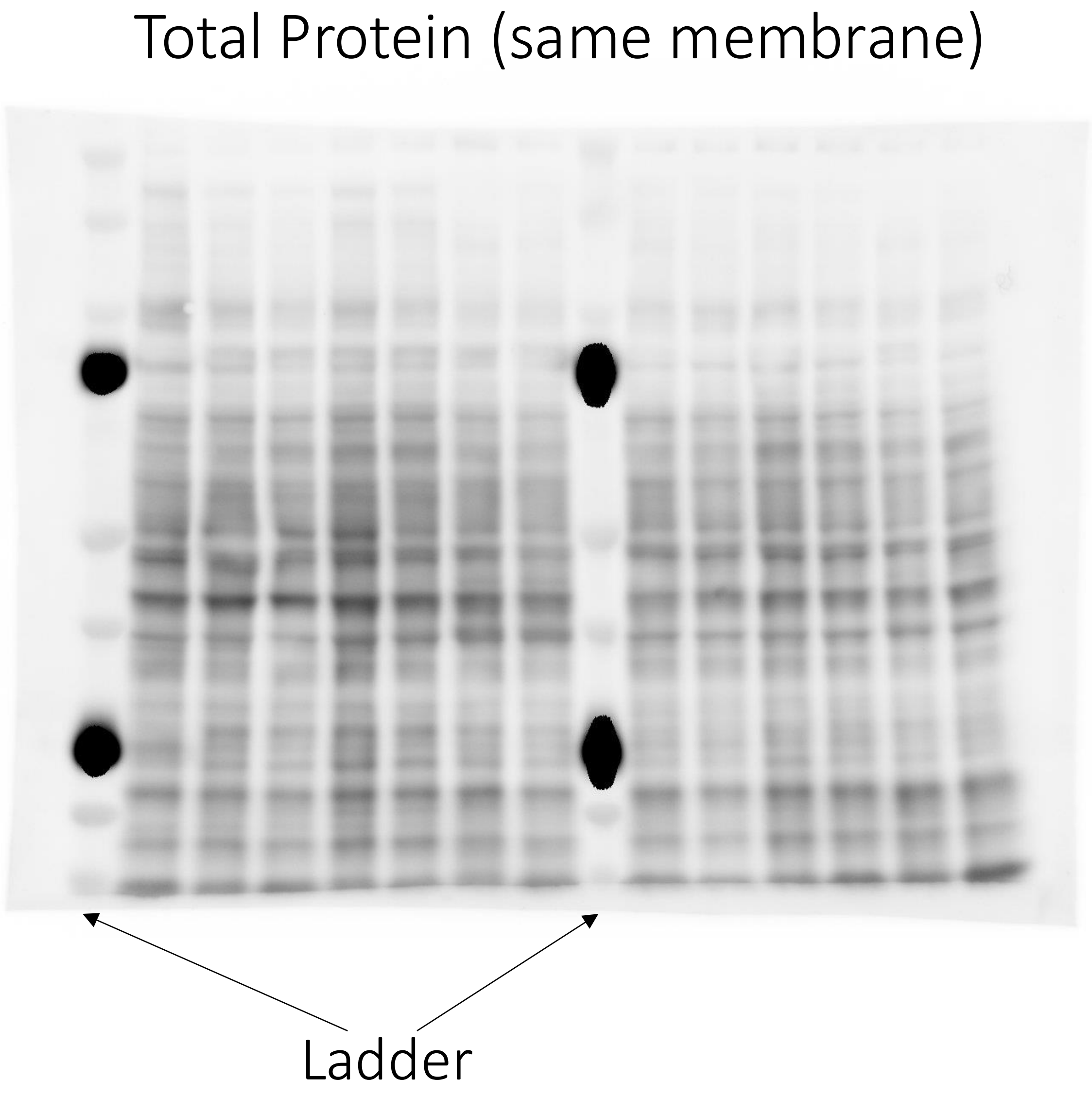

All regions batch 4: 19-20

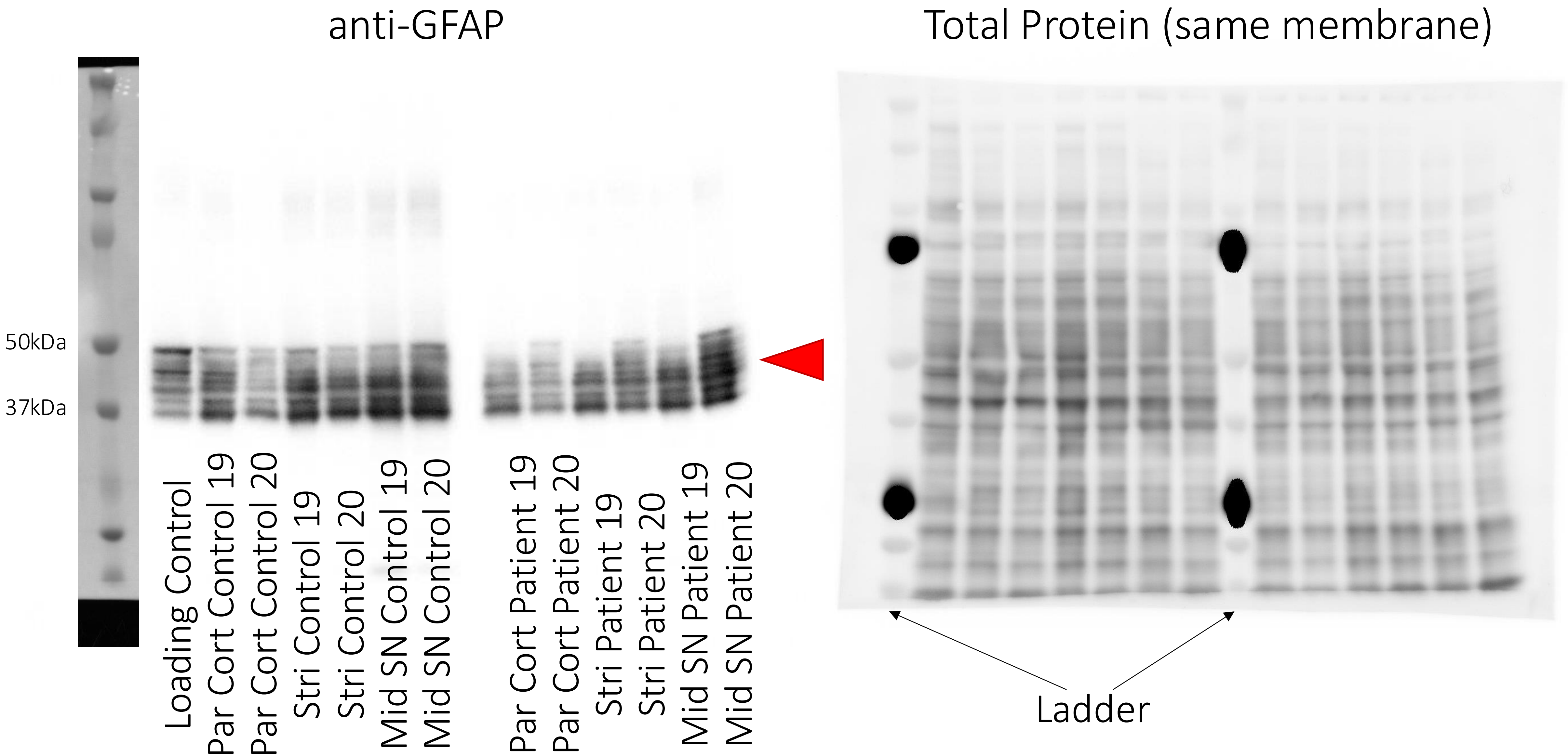

All regions batch 4: 19-20

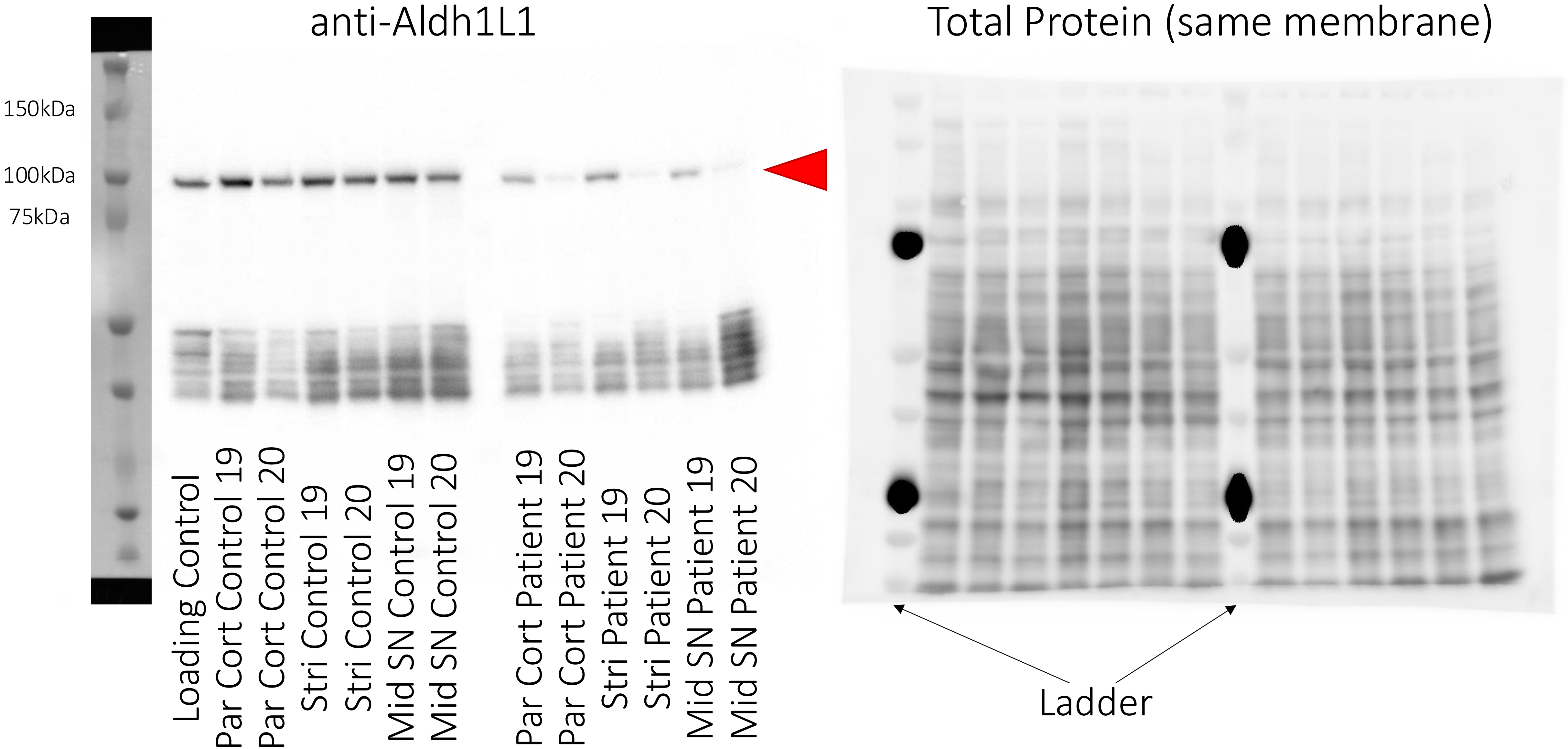

Supplement: Supplementary file 1 — Supplementary Information 1. [file 41598_2025_94188_MOESM1_ESM.pdf]
