# Supplementary material for: Connexin 43 is downregulated in advanced Parkinson’s disease in multiple brain regions which correlates with symptoms
Source: Sci Rep. 2025 Mar 25;15:10250. doi: 10.1038/s41598-025-94188-7 (PMC11937269; doi:10.1038/s41598-025-94188-7)
Supplement: Supplementary file 2 — Supplementary Information 2. [file 41598_2025_94188_MOESM2_ESM.pdf]

Supplementary materials for:

Connexin 43 is downregulated in advanced Parkinson's disease in multiple brain regions which correlates with symptoms

Nataly Hastings*[1,2,3], Saifur Rahman[1,2], Przemyslaw Aleksander Stempor[4], Matthew T. Wayland[5], Wei-Li Kuan[1,6], Mark R.N. Kotter[1,2]

Affiliations:

1 - Department of Clinical Neurosciences, University of Cambridge, Cambridge, CB2 0QQ, United Kingdom

2 - Wellcome-MRC Cambridge Stem Cell Institute, University of Cambridge, Cambridge, CB2 0AW, United Kingdom

3 - Electrical Engineering Division, Department of Engineering, University of Cambridge, Cambridge, CB3 0FA, United Kingdom

4 -Wellcome Trust / Cancer Research UK Gurdon Institute, University of Cambridge, Cambridge, CB2 1QN, United Kingdom

5 - Department of Zoology, University of Cambridge, Cambridge, CB2 3EJ, United Kingdom

6 - Alborada Drug Discovery Institute, University of Cambridge, Cambridge, CB2 0AH, United Kingdom

*Corresponding author: Dr Nataly Hastings, nh564@cam.ac.uk, ORCID: 0000-0002-5376-339X

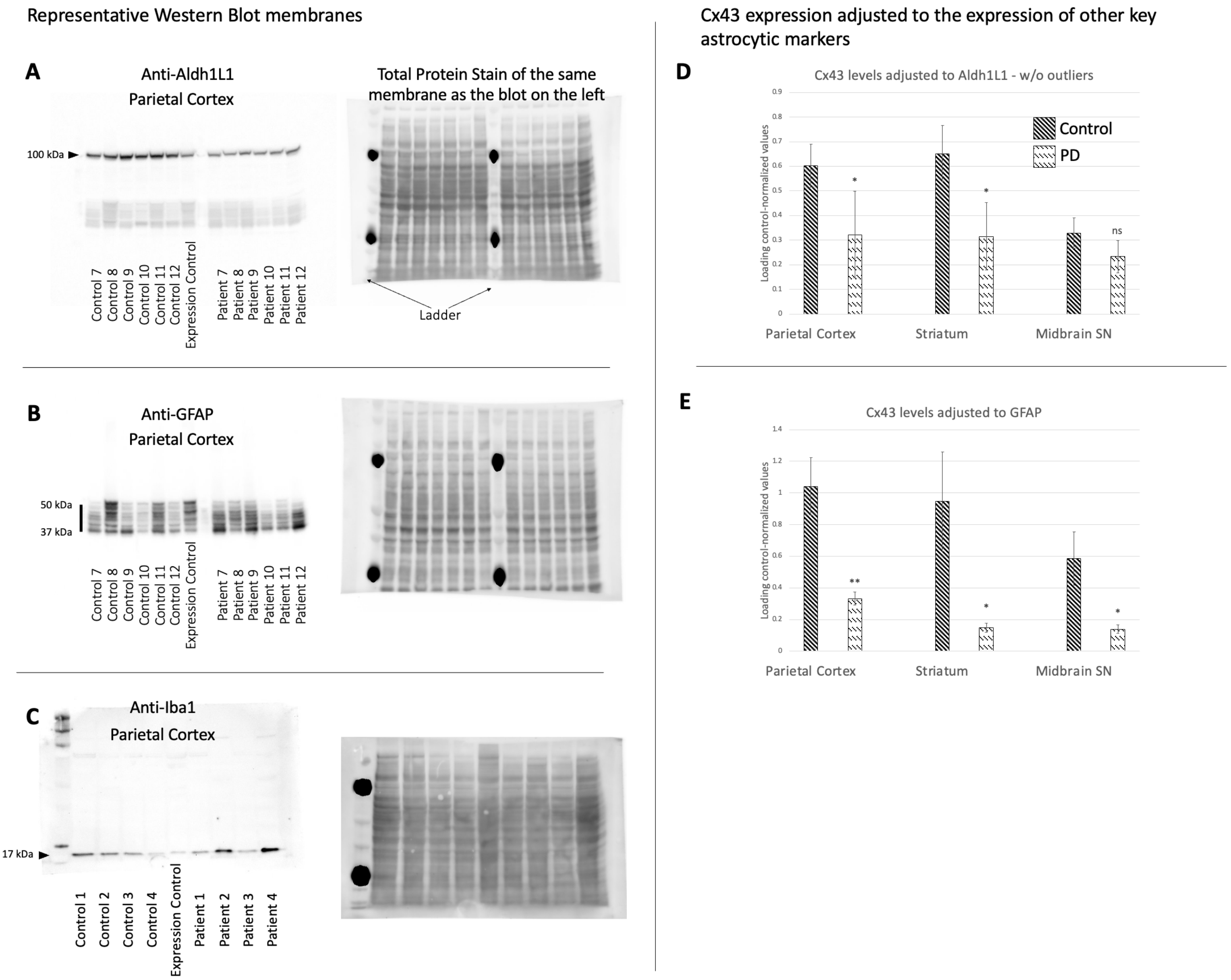

Supplementary Figure 1. *Representative Western blot membranes of the stainings against several astrocytic markers and a microglial marker in control and PD cases, and Cx43 protein levels adjusted to the expression of other key astrocytic markers.* **A:** Aldh1L1 astrocytic marker staining (shadow staining at a lower molecular weight is background left from the prior GFAP staining on the same membrane). **B:** GFAP astrocytic marker staining, a range of detected isoforms. **C:** Iba1 microglial marker staining. For all images, staining on the left side is enhanced chemiluminescence (ECL) signal on a PVDF membrane, and staining on the right side is far red (700nm) Total Protein fluorescent signal read from the same membrane as shown on the left. Expression Control is a sample from a control parietal cortex (representing the relative value of 1, same for all membranes) which was ran

on each membrane among experimental samples to allow for a semi-quantitative relative signal comparison between membranes. All Western blot membranes presented are full images as obtained; no editing, cropping, or other image manipulation was introduced (other than digital inverting for "dark bands on light background" conventional representation purposes). **D-E:** Cx43 protein levels were adjusted to the expression of Aldh1L1 and GFAP in the same sample (imaged on the same membrane), respectively. In case of the Cx43/Aldh1L1 analysis, 5 outliers were identified using the Z-score method (over 3 standard deviations from the average), or which 3 most severe outliers were found in the midbrain; outliers were removed from the representative graph for the ease of the visual trend assessment. n=20 per group except: Cx43/Aldh1L1 in Mid SN control (n=17, 2 missing samples and 1 outlier), Cx43/Aldh1L1 in Midbrain SN PD (n=18, 2 outliers), Cx43/Aldh1L1 in Striatum PD (n=19, 1 outlier), Cx43/Aldh1L1 in Parietal Cortex PD (n=19, 1 outlier), Cx43/GFAP in Midbrain SN PD (n=18, 2 missing samples). Statistical analysis: ANOVA; error bars: SEM. *p<0.05, **p<0.005. PD – Parkinson's disease; Cx43 – connexin 43; GFAP – glial fibrillary acidic protein; SN – substantia nigra.

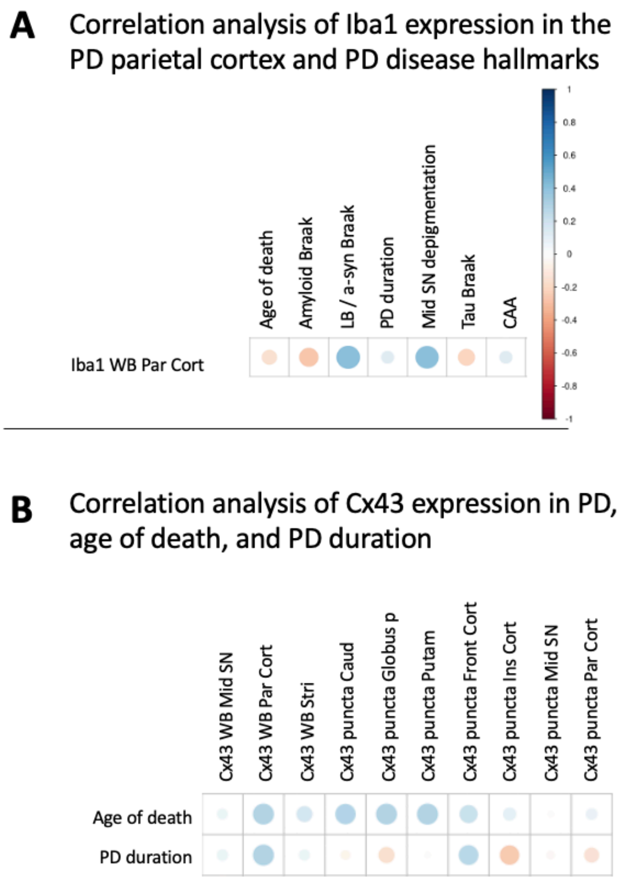

Supplementary Figure 2. *Additional correlation analyses.* **A:** Correlation analysis of the Iba1 (microglial marker) expression detected in the parietal cortex samples via Western blot and a number of PD hallmarks (n=20, PD cohort only); correlation coefficients and p-values are shown in Supplementary Table 6. **B:** Correlation analysis of Cx43 expression with age and PD duration (n=20, PD cohort only). For both images, heatmap scale represents the value of the Spearman's correlation coefficient with blue colour indicative of positive correlation and red – of negative correlation (or anti-correlation); correlation coefficients and p-values are shown in Supplementary Table 7. PD – Parkinson's disease; Cx43 – connexin 43; WB – Western blot; LB – Lewy body; a-syn – alpha-synuclein; CAA – cerebral amyloid angiopathy; Mid SN – midbrain substantia nigra; Par Cort – parietal cortex; Stri – striatum; Caud – caudate; Globus p – globus pallidus; Putam – putamen; Front Cort – frontal cortex; Ins Cort – insular cortex.

GJA1 (bulk) expression in midbrain SN in
different stages of PD

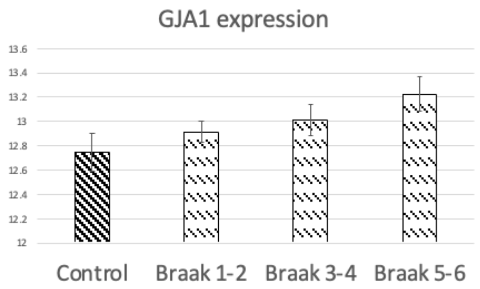

Supplementary Figure 3. *Bulk GJA1 (Cx43 encoding gene) analysis in association with PD progression.* GJA1 expression data was obtained from a published dataset [1] and re-analysed de novo. Expression in control cases (n=8) was compared with early-stage PD (Braak stages 1-2, n=5), mid-stage PD (Braak stages 3-4, n=7), and late-stage PD (Braak stages 5-6, n=8). Statistical analysis: ANOVA; error bars: SEM. PD – Parkinson’s disease.

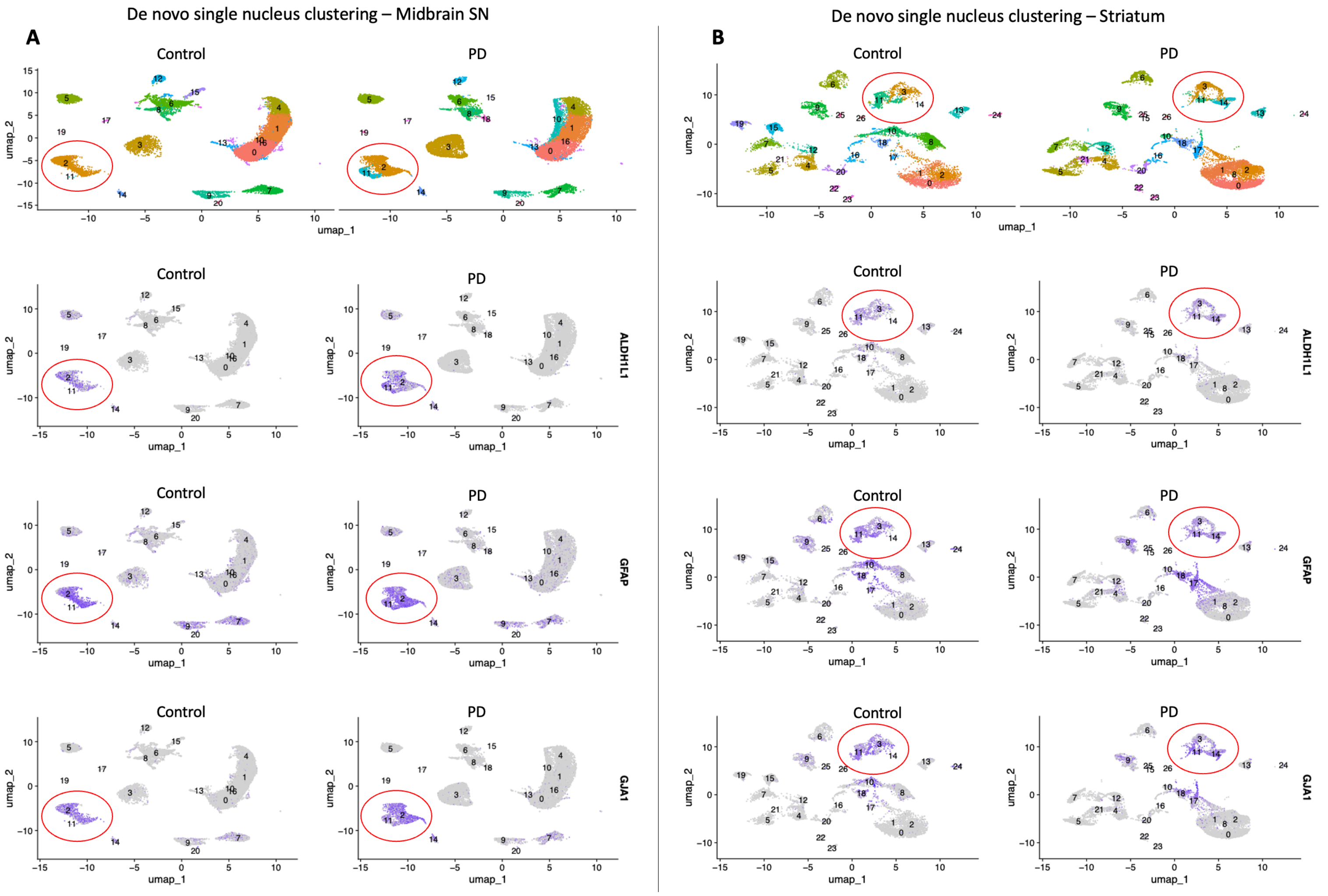

Supplementary Figure 4. *De novo identification of astrocyte clusters from single cell datasets.* **A:** An existing single cell dataset [2] from the midbrain SN (n=5 controls and n=6 PD) was mapped on PCA plots and cell lineage marker analysis was applied to identify cell clusters most likely representing mature astrocytes. Purple colour of individual dots (each dot representing a single cell) indicates the expression of a particular marker as labelled on the right side of the plots, with a deeper purple colour indicative of stronger expression patterns. Co-expression of Aldh1L1, GFAP, and GJA1 was indicative of the astrocytic identity of the cluster circled in red. **B:** An existing single cell dataset from the striatal tissue [3] (n=4 controls and n=4 PD) was mapped on PCA plots using the same method as for the midbrain dataset, and an astrocytic cell cluster was identified in the same manner. Astrocytic clusters from both regions were used for further GJA1 expression analysis. PD – Parkinson's disease; Midbrain SN – midbrain substantia nigra; GFAP – glial fibrillary acidic protein.

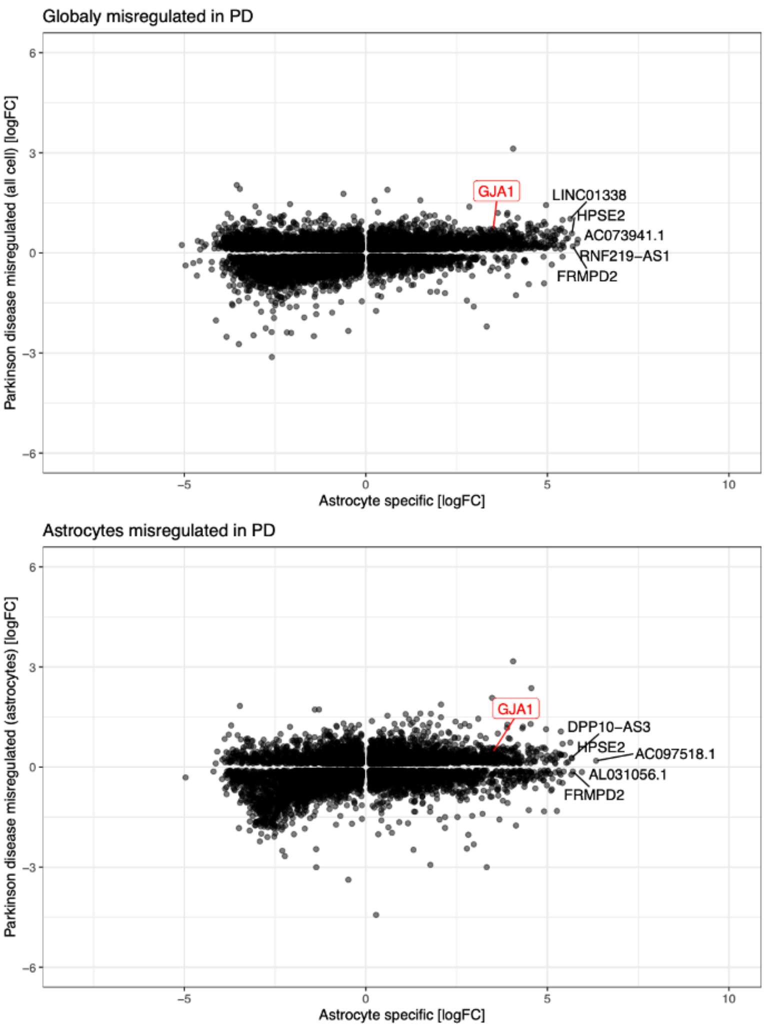

test	gene	p_val	p_val_adj	avg_log2FC
astro	GJA1	0.000000e+00	0.000000e+00	3.5107007
pd_all	GJA1	0.000000e+00	0.000000e+00	0.7261449
pd_ast	GJA1	2.520165e−117	7.529749e−113	0.4656711

Supplementary Figure 5. *GJA1 (Cx43 encoding gene) mRNA upregulation in human midbrain SN in PD.* An existing single cell dataset [4] from the midbrain SN (n=14 controls and n=15 PD) was re-analysed for the expression of GJA1 globally (taking all cell types into account) and specifically in astrocytes. PD – Parkinson's disease; Cx43 – connexin 43

Supplementary Table 1. *Patient demographics*. Notes on the cases used in the present study. “N/A” in the symptoms section indicates that clinical histories were not available for examination. Motor and gastrointestinal symptoms were not included, as they were present in all PD patients whose clinical histories were available. LB – Lewy body; SN – substantia nigra; CAA – cerebral amyloid angiopathy; PD – Parkinson’s disease; LBD – Lewy body dementia; PSP – progressive supranuclear palsy; UTI – urinary tract infection; COPD – chronic obstructive pulmonary disease.

	Brain Bank code	Dementia	Depression	Memory problems	Sleep disturbance	Aggression	Hallucinations	Psychotic symptoms	LB disease type	Braak stage LB pathology	Braak stage Amyloid pathology	Braak stage Tau pathology	SN depigmentation	CAA	Age	PD duration	Gender	Cause of death	Notes
**Patient1**	**PD0818**	+	+	+	+	+	+		limbic	5	1	2	++		75	10	M	PD	
**Patient2**	**PD0844**	+	+	+	+	+	+	+	limbic	6	0	1	+++	++	72	32	M	PD; pneumonia	Young-onset PD
**Patient3**	**PD0851**	N/A	N/A	N/A	N/A	N/A	N/A	N/A	neocortical	6	3	3	+++		83	31	F	PD; pneumonia	
**Patient4**	**PD0863**		+		+				brainstem	5	0	1	+++		88	10	M	PD; pneumonia; sepsis	
**Patient5**	**PD0865**	+			+	+	+	+	neocortical	6	3	3	++		78	15	M	PD; LBD; UTI	
**Patient6**	**PD0887**	+					+		neocortical	5	3	2	++		76	9	M	PD	
**Patient7**	**PD0940**				+				limbic	5	3	3	+		81	8	F	PD; pneumonia	
**Patient8**	**PD0955**	+			+	+	+		neocortical	5	3	3	+++		85	20	M	PD; pneumonia; dementia	
**Patient9**	**PD0957**	+		+			+		neocortical	6	2	1	+++	+	76	26	F	PD	
**Patient10**	**PD0985**		+		+				limbic	6	2	1	+++	+	70	6	M	LBD; cancer	
**Patient11**	**PD0989**				+				neocortical	6	2	2	+++		91	11	M	PD; cardiac failure; sigmoid volvulus	
**Patient12**	**PD0991**	+	+		+		+	+	neocortical	6	3	2	+++	+++	68	10	M	PD; cancer	
**Patient13**	**PD1003**	+	+	+	+		+	+	neocortical	6	0	3	+++		71	22	M	PD	
**Patient14**	**PD1004**	+				+	+		neocortical	6	0	1	++	++	87	10	M	PD	
**Patient15**	**PD1023**	+	+	+	+		+		neocortical	6	1	3	+++		79	14	M	PD	Ageing Related Tau Astrogliopathy in basal ganglia, mid SN
**Patient16**	**PD1049**	+	+	+	+	+	+	+	neocortical	5	3	3	++	+	87	20	M	PD; pneumonia	Ageing Related Tau Astrogliopathy in basal ganglia, hippocampus
**Patient17**	**PD1059**	N/A	N/A	N/A	N/A	N/A	N/A	N/A	neocortical	6	0	3	++	+	87	11	F	PD	
**Patient18**	**PD1086**	+		+	+		+		neocortical	6	3	2	+++	+++	78	20	M	PD; LBD	Potentially atypical PD / PSP
**Patient19**	**PD1097**	+	+	+	+		+		neocortical	6	5	3	+++	+++	65	20	F	PD; dementia	
**Patient20**	**PD1113**	+	+	+	+	+	+		neocortical	6	4	3	+++	+	80	15	M	PD	
**Control1**	**C020**			+					N/A						84	N/A	F	Cardiac failure	
**Control2**	**C037**								N/A						84	N/A	M	Pneumonia; cancer	
**Control3**	**C074**								N/A						84	N/A	F	Cardiac failure; old age	
**Control4**	**C084**	N/A	N/A	N/A	N/A	N/A	N/A	N/A	N/A	0	2	0		+	84	N/A	F	Old age	
**Control5**	**C085**	N/A	N/A	N/A	N/A	N/A	N/A	N/A	N/A	0	3	2		++	81	N/A	F	Pneumonia; cardiac failure	
**Control6**	**C087**								N/A	0	1	2		+	94	N/A	F	Cardiac failure	
**Control7**	**C090**	N/A	N/A	N/A	N/A	N/A	N/A	N/A	N/A	0	1	2			83	N/A	M	Pulmonary fibrosis	
**Control8**	**C092**	N/A	N/A	N/A	N/A	N/A	N/A	N/A	N/A	0	4	2			93	N/A	F	N/A	
**Control9**	**C094**	N/A	N/A	N/A	N/A	N/A	N/A	N/A	N/A	0	0	0			47	N/A	F	Cancer; pulmonary embolism	
**Control10**	**PDC027**								N/A						89	N/A	M	Pneumonia; cardiac failure	Agyrophilic grain disease (tau) possible
**Control11**	**PDC034**								N/A						90	N/A	M	Respiratory failure; cancer	
**Control12**	**PDC040**	N/A	N/A	N/A	N/A	N/A	N/A	N/A	N/A						61	N/A	F	Cancer	
**Control13**	**PDC087**								N/A	0	3	3		++	92	N/A	F	N/A	
**Control14**	**PDC091**								N/A	0	3	2		+	85	N/A	M	Pneumonia	
**Control15**	**PDC107**								N/A	0	3	2		+	87	N/A	F	Cardiac failure; COPD; iscaemic leg; stroke	Ageing Related Tau Astrogliopathy in hippocampus
**Control16**	**PDC114**								N/A	0	3	1			70	N/A	M	Iscaemia; sepsis; obesity; type 2 diabetes	
**Control17**	**PDC123**								N/A	0	5	2		+++	92	N/A	M	Pneumonia	
**Control18**	**PDC126**	+	+	+					N/A	0	0	2			82	N/A	M	Pneumonia; cardiac failure; kidney injury; osteomyelitis	Ageing Related Tau Astrogliopathy in hippocampus and basal ganglia
**Control19**	**PDC128**		+		+				N/A	0	2	1			91	N/A	F	Cardiac failure	Ageing Related Tau Astrogliopathy in hippocampus
**Control20**	**PDC131**								N/A	0	3	1		+	92	N/A	F	Pneumonia; COPD	

Supplementary Table 2. *Extended notes on patients' histories.* Notes on the cases used in the present study that include medications and treatments plus various pathological findings in the brain. PD – Parkinson's disease; MS – multiple sclerosis; SN – substantia nigra; R – receptor; NSAID – non-steroid anti-inflammatory drugs; DHT – dihydrotestosterone; REM – rapid eye movement; AD – Alzheimer's disease; a-syn – alpha-synuclein; SSRI – selective serotonin reuptake inhibitors; LHRH – luteinising hormone-releasing hormone; UTI – urinary tract infection; MAO-B – monoamine oxidase B; DBS – deep brain stimulation.

	Age of death	Cause of death	Gender	Time to preservation	Medication and treatments	Clinical history	Pathological findings in the brain
C1 - C020	84	Heart disease	F	24h	Antiplatelet agents	Myelodisplasia	Small infarcts incl par cort; some memory loss; few beta-amyloid plaques and tau tangles
C2 - C037	84	Cancer, pneumonia	M	5h	Amiodarone, warfarin	Relation with MS	Some beta-amyloid plaques and tau tangles in front cort
C3 - C074	84	Old age (Heart disease + kidney failure)	F	22h	Steroids	Hypothyroidism; son with MS; polymyalgia rheumatica	Moderate atrophy of frontal lobe; SN slightly pale; moderate tau pathology
C4 - C084	84	Old age	F	23h			Mild tau and amyloid changes
C5 - C085	81	Respiratory failure, pneumonia, atrial fibrillation, hypertension	F	22h			Tau and amyloid pathology present in cortices, amyloid in basal ganglia
C6 - C087	94	Heart failure	F	24h		Pneumonia, valvular heart disease, atrial fibrillation, left ventricular failure	Some loss of pigmented neurones in SN; very mild tau and amyloid changes
C7 - C090	83	Pulmonary fibrosis	M	21h			Mild tau and amyloid pathology
C8 - C092							Moderate amyloid pathology, mild tau pathology
C9 - C094	47	Pulmonary embolism, metastatic ovarian cancer	F	30h		Potential brain metastasis	No neurodegenerative changes
C10 - PDC027	89	Cardiac arrest; pneumonia	M		Cholecystectomy; beta1 adrenergic R blockers; thiazide diuretics; loop diuretic; NSAID; amlodipine (calcium channel blocker in the heart); antibiotic	Hypotension; bradycardia; impaired glucose tolerance; hyperkeratosis	No signs of raised intracranial pressure; some atherosclerosis; diffuse iscaemic damage; few senile plaques (amyloid) in cort; corpora amylacea in ins cort; neuronal atrophy in several brain areas; severe loss of Purkinje cells and inflammation in the cerebellum; hipothal and amygdala are "gliotic"; severe tau tangles in amygdala and temp cort; astrocytic tau; tau pathology consistent with agyrophilic grain disease; mild acute leptomeningitis
C11 - PDC034	90	Respiratory failure; bronchial cancerr	M		Ureterolithotomy; appendectomy; repair of incisional hernia; cataract extraction; steroids; cyclopyrrholone (for insomnia); proton pump inhibitor (for gastric ulcer); antibiotic; bronchodilator (antimuscarinic); beta2 adrenergic R agonist (salbutamol); angiotensin R blocker (for hypertension); alpha1a adrenergic R antagonist (for prostate); beta adrenergic blocker (for glaucoma, hypertension); carbonic anhydrase inhibitors (for glaucoma, hypertension); antibiotics; anti-androgen (DHT blocker); opioid; NSAIDs; thiazide diuretics; antimuscarinics (for breathing difficultires, nausea?); antihistamine; antiviral (Famvir for herpes); ferrous sulphate (for anaemia); calcium channel blocker (for hypertension); angiotensin converting enzyme inh (for hypertension); antifungal	Gout; hydronephrosis; hernia; gack problem; glaucoma; tendonitis; bronchitis; otitis media; hyperglycaemia; haemorrhoids; hearing loss; chronic rhinitis; skin lesion; melanea; diverticular disease; hypertension; keratosis; atrial fibrillation; malignant neoplasm of bronchus	Mild leptomeningeal fibrosis; mild perivascular oedema; mild loosening of perivascular glial texture in basal ganglia; many corpora amylacea; mild neuronal loss in hippocampus; ,ild gliosis of Bergmann's glia in cerebellum; mild reduction in Purkinje cells; occasional tau pretangles and tangles; mild age-related and microvascular changes
C12 - PDC040	61	Ovarian cancer	F				Mild perivascular oedema; many corpora amylacea; mild Bergmann's gliosis and mild gliosis of the dentate nucleus; few tau tangles in amygdala and hippocampus; mild vasular changes in the globus pallidus
C13 - PDC087	92		F		Replacement of left knee; hysterectomy; angiotensin converting enzyme inhibitor (for hypertension); calcium channel blocker (for hypertension); antihistamine	Son and daughter with PD; vertigo; peritonsillar abscess; syncope; oesophageal ulcer; Bowen's disease (neoplastic skin disease); osteoarthritis; diverticulitis	Severe atherosclerosis; low AD change; amyloid and tau pathology present
C14 - PDC091	85	Lobar pneumonia	M		Pacemaker; lumbar disc laminectomy (for arthritis of the spine); aortic valve replacement; cataract surgery; anticoagulants; antiandrogen (DHT blocker)	Bradycardia; glaucoma; benign prostatic hypertrophy; aortic stenosis; keratosis; hearing loss; cataract; pneumonia, coughing up blood, confusion	Very mild cerebral amyloid angiopathy; amyloid and tau pathology present; low AD change
C15 - PDC107	87	Acute leg ischaemia; heart failure; COPD	F		Blood transfusion due to diverticulitis; hip replacement; statin; beta1 adrenergic receptor blocker (for heart disease); vitD suppl; calcium suppl; loop diuretic; sulfonylurea type anti-diabetic (stimulates insulin secretion); proton pump inhibitor (for gastric ulcer / reflux); xanthine oxidase inhibitor (dicreases uric acid)	Incontinence; Charles Bonnett Syndrome (visual hallucinations in blind); diverticulitis, constipation, nausea; leg ulceration; hypercholesterolemia; hypertension; atrial fibrillation; type II diabetes; obstructive sleep apnoea; gout; chronic kidney disease; spinal stenosis; osteoarthritis; polymyalgia rheumatica; abdominal aortic aneurism; ischaemic attack	Mild atheroma; slight SN depigmentation; cerebellar infarct; few basal ganglia lacunes; very mild meningeal cerebral amyloid angiotpahy; amyloid and tau pathology present; ageing related tau astrogliopathy; low AD change
C16 - PDC114	70	Ischaemic bowel; biliary sepsis; ischaemic heart disease; type II diabetes; obesity	M		Amputation of toes; cataract extraction; coronary artery bypass graft; antibiotics; opioid; H2 histamine R antagonist (for gastric ulcer); statin; NSAID; beta1 adrergic R blocker (for heart disease); loop diuretic; calcium channel blocker (for hypertension); alpha1a adrenoreceptor antagonist (for prostate); angiotensin R blocker (for hypertension); proton pump inhibitor; insulin	Obesity; constipation / diarrhoea; small bowel bacterial overgrowth; type II diabetes (under poor control), high blood glucose; leg pain; stenosis of popliteal arteries; foot ulceration; iron deficiency anaemia; abdominal pains; gastritis; slowed GI transit; prostatism; cataract; osteoarthritic; hypertension; hypercholesteremia; angina pectoris; inflammatory bowel syndrome; diabetic retinopathy; hernia; stroke	Atheroma; artery stenosis; mild age-associated dopaminergic neurone loss; some amyloid and tau pathology present; low AD change
C17 - PDC123	92	Bronchopneumonia	M		Histamine H3 R antagonist (betahistine, for vertigo); beta1 adrenergic R antagonist (for hypertension); loop diuretic; prostaglandin F agonist eye drop (for glaucoma); proton pump inhibitor; anticoagulant; statin; DHT blocker (antiandrogen, for prostate); quinine sulfate (for leg cramps?); ferrous fumarate (for anaemia)	Haematuria; visual disturbance; fall; angina; limited mobility; shortness of breath; vertigo; lymphoedema; leg ulcers; lower respiratory tract infection; anaemia; chest infection and sepsisspondylosis; cataract; migraine; diverticulosis; Menier's disease; arteritis; osteoarthritis; tinnitus; glaucoma; atrial fibrillation; ischaemic heart disease; gastro-oesophageal reflux; congestive heart failure; cavernous haemangioma	Severe cerebral amyloid angiopathy; amyloid and tau pathology present; low AD change
C18 - PDC126	82	Pneumonia; congestive heart failure; kidney injury; osteomyelitis	M		Steroid (inhaled); beta2 adrenergic R agonist (bronchodilator); antimuscarinic (inhaled); insulin; NSAID; laative; potassium-sparing diuretic (also antiandrogen); loop diuretic; cyclopyrrolone (for sleeping); antagonist of voltage-gated ca channels (Pregabalin, for pain); vit D suppl; Ca suppl; beta1 adrenergic R antagonist (for hypertension); statin	Memory problems; likely sleep problems (indicated by the insomnia medication); mild cognitive impairment; lack of motivation; reduced mobility; cellulitis; lower respiratory tract infection; sepsis; anaemia; type II diabetes (poorly controlled); leg ulcers; COPD; peripheral neuropathy; obesity; hypertension; osteoarthritis; congestive heart failure; chronic kidney disease; small cerebellar infarct	Aging related tau astrogliopathy; mild age-associated dopaminergic neurone loss in SN; amyloid and tau pathology present; low AD change
C19 - PDC128	91	Cardiac failure; hypotension	F		Appendectomy; shoulder replacement; cardiac pacemaker; hearing aid; mirtazapine (atypical antidepressant, sleep aid); histamine H2 antagonist (for gastric acid regulation); hydroxychloroquine (anti-rheumatic); ferrous fumarate (for anaemia); vit C suppl; vit D suppl; anticoagulant; alendronic acid (blocks bone resorption); opioid (peripheral, for diarrhoea); NSAID	Shortness of breath; falls; hyponatraemia; urosepsis; very mildly forgetful; right facial drop; severe aortic stenosis; low mood; poor sleep; systemic vasculitis; Sjögren's syndrome (autoimmune); hypertension; congestive heart failure; angina; hernia; hyperthyroidism; ischaemic heart disease; atrial fibrillation; chronic kidney disease	Aging related tau astrogliopathy; mild age-associated dopaminergic neurone loss in SN; amyloid and tau pathology present; low AD change
C20 - PDC131	92	Pneumonia; COPD; cerebrovascular disease	F		Myomectomy; oxygen therapy; histamine H2 antagonist (for gastric acid regulation); sodium potassium adenosine triphosphatase inhibitor (for cardiac disease); beta1 adrenergic R antagonist (for hypertension); cyclopyrrolone (for insomnia); antagonist of voltage-gated ca channels (Pregabalin, for pain); vit D suppl; Ca suppl; statin; anticoagulant	Back pain; spasms; UTIs; left side weakness; chest sepsis; cerebrovascular accident (stroke); osteoarthritis; polymyalgia rheumatica; kyphoscoliosis; hypertension; uterine fibroids; diverticular disease; respiratory failure; COPD	Mild neuroangiopathy; ectasia of the proximal basilar artery; severe atheroma; fusiform aneurismatic enlargement of the left vertebral artery; moderate atrophy of cortex; very mild age-associated dopaminergic neurone loss in SN; mild meningeal cerebral amyloid angiopathy; amyloid and tau pathology present; low AD change

	Age of death	Cause of death	Gender	Time to preservation	Medication and treatments	Clinical history	Pathological findings	Recorded disease duration	Age of onset
P1 - PD818	75	End-stage PD	M		Levodopa; dopamine agonists; cholinesterase inhibitor; Modafinil; antipsychotic; NSAIDs; steroids; diamorphine; omeprazole (proton pump inh - gastric reflux); cholesterol-lowering; gabapentin (neuropathic pain, anticonvulsant); laxative; Amlodipine (calcium channel blocker for high BP)	Idiopathic PD, vascular PD, PD dementia; memory problem; poor sense of smell; tremor; rigidity; freezes; loss of voice; REM disorder; mild hallucinations; aggression; double vision; mild depression; fatigue; weight loss; constipation; loss of taste; incontinence	Mild atrophy of frontal lobe; moderate depigmentation of SN; very mild amyloid and tau pathology; Lewy body Braak stage 5; limbic type of Lewy body disease; no inclusions in par and front cort	10y	66
P2 - PD844	72	Pneumonia; PD	M		Levodopa; dopamine agonists; GABA agonist; pallidectomy; SSRI for obsessive behaviour; vit D3 suppl; NSAIDs; Buscopan (anticholinergic for treatment of stomach cramps); laxatives	Young-onset PD; psychosis; cognitive impairment; severe motor impairment; IBS/constipation; impulse control disorder; dyskinesias and dystonias (drug-induced); hallucinations; abdominal pain; forgetful; anger and aggression; sleep disturbance; paranoid delusions; thermal dysregulation; depression; hypersexuality	Severe SN depigmentation; a-syn aggreg in astrocytes in cingulate gyrus, amygdala, hippocampus; Lewy body Braak stage 6; limbic type of Lewy body disease, mildly affected cortices; moderate-severe small vessel disease in basal ganglia; no amyloid pathology; mild tau pathology	32y	40

P3 - PD851	83	PD	F				Severe SN depigmentation; neocortical type of Lewy body disease; Lewy body Braak stage 6; mild amyloid pathology in hippocamous, entorhinal cortex and striatum; severe tau pathology in hippocampus, entorhinal cortex and occipito-temporal gyrus (intermediate AD)	31y	52
P4 - PD863	88	Pneumonia; PD; Biliary sepsis secondary to gallstones	M		Levodopa; dopamine agonist; Mirtazapine (antidepressant+insomnia treatment); Clonazepam (benzodiazepine); steroid; laxative; antacid	Idiopathic PD; gait disturbance; rapid mobility deterioration; postural hypotension; depression; dyskinesias; incontinence; low mood; panic disorder; shingles; REM sleep disorder; Basal cell carcinoma – right hand; acute cholecystitis and biliary sepsis	Marked SN depigmentation; Lewy body Braak stage 5; neocortical Lewy body and Lewy neurites (cingulate and entorhinal); brainstem type of Lewy body disease; no amyloid or tau pathology	10y	79
P5 - PD865	78	Urinary tract infection; PD; Lewy body dementia	M		Levodopa; MAO-B inhibitor; dopamine agonist; cholinesterase inhibitor; Quetiapine (atypical antipsychotic, antidepressant); Solifenacin (overactive bladder treatment); antibiotic	Tremor; insomnia; fatigue; rigidity; bradykinesia; dyskinesia; obsessive behaviours, punding; hypersexuality; hypophonia; incontinence; sialorrhoea; hallucinations; impulsivity; constipation; confusion; aggression; dysphagia; Lewy body dementia	Moderate SN depigmentation; neocortical a-syn pathology, Lewy body Braak stage 6; neocortical Lewy body disease; moderate amyloid and tau pathology (intermediate AD)	15y	64
P6 - PD887	76	End-stage PD	M		Levodopa; dopamine agonist; Pizotifen (migraine treatment, serotonin antagonist); Citalopram (SSRI); Zopiclone (insomnia treatment); Clonazepam (benzodiazepine); Finasteride (anti-DHT for enlarged prostate?); vit D3 suppl	Idiopathic PD; AD; motor difficulties: disturbing tremor, rigidity, restless legs; constipation; hallucinations; lightheadedness, postural hypotension; dysphagia, dysarthria; disorientation, cognitive decline; urinary frequency; UTI; migraines	Moderate SN depigmentation; a-syn pathology in brainstem, limbic system and neocortex; neocortical Lewy body disease; Lewy body Braak stage 5; amyloid and tau pathology present	9y	65
P7 - PD940	81	Aspiration pneumonia; PD	F		Levodopa; Clonazepam (benzodiazepine); Amitriptyline (atypical antidepressant, paintreatment); NSAIDs; laxatives; antibiotic	Idiopathic PD; tremor; bradykinesia; fatigue; dysphonia; hypomimia; constipation; incontinence; REM sleep disorder, poor sleep, drowsiness; minor stroke; fear	Mild SN depigmentation; a-syn pathology mainly in the brainstem and limbic system; limbic Lewy body disease, Lewy body Braak stage 5; amyloid and tau pathology present (intermediate AD)	8y	72
P8 - PD955	85	Aspiration pneumonia; PD with dementia	M		Levodopa; dopamine agonist; cholinesterase inhibitor; benzodiazepine; antibiotics	Idiopathic PD; dementia; falls; weight loss; dyskinesias; rigidity; bradycardia - has a pacemaker; poor balance; confusion; hallucinations; aggression; dystonia; constipation; blood in urine, UTI; dysphagia/swallowing problem; some sleep issue; polymyositis; Coeliac disease	Marked SN depigmentation; severe neuronal loss in mid SN; a-syn pathology in the brainstem, limbic system and neocortex; neocortical Lewy body disease; Lewy body Braak stage 5; amyloid and tau pathology present (intermediate AD)	20y	65
P9 - PD957	76	PD	F		Levodopa; NSAIDs; benzodiazepines; opioid; steroid; anti-convulsant; Glycopyrronium bromide (antimuscarining, peripheral); proton pump inhibitor (gastric acid block); laxatives	Some difficulty walking; dystonia in toes; memory and concentration problems; clumsiness of fingers; bradykinesia; dyskinesia (drug-induced); DBS - led to hemiparesis; freezing; confusion; hallucinations; speech difficulties, aphasic; epilepsy; dementia; incontinence; constipation; subarachnoid haemorrhage; arthritis; glaucoma; histerectomy	Severe SN depigmentation; atheroma; old infartct in the R thalamus; cerebral vascular disease; a-syn pathology in the brainstem, limbic system and neocortex; neocortical Lewy body disease; Lewy body Braak stage 6; amyloid pathology; mild tau pathology (low AD changes)	26y	50
P10 - PD985	70	Lewy body dementia; prostate cancer	M		Levodopa; benzodiazepine; cholinesterase inhibitor; statin; SSRI	Stiffness; REM sleep disturbance; micrographia; quiet voice; dyskinesia; some tremor; depression	Severe SN depigmentation; a-syn aggreg in astrocytes in cingulate gyrus, amygdala, hippocampus; Lewy body Braak stage 6; limbic type of Lewy body disease, mildly affected cortices; moderate-severe small vessel disease in basal ganglia; mild amyloid and tau pathology	6y	64
P11 - PD989	91	PD; heart disease; sigmoid volvulus	M		Levodopa; dopamine agonists; antibiotics; DOPA decarboxylase inh (peripheral); opiate; benzodiazepine; MAO-B inhibitor;	Idiopathic PD; micrographia; loss of smell; hypomimia; dystonia; bradykinesia; rigidity; tremor; blepharospasm, ectropion; dysarthrophonia; leg cellulitis; weight loss, poor appetite; back pain, crush fracture in L3, L5 vertebae, osteopenia, kyphoplasty; constipation; REM sleep disorder?; double vision; chest infection; prostatectomy; hypertension; congestive heart failure	Severe SN depigmentation; a-syn pathology in the brainstem, limbic system and neocortex; neocortical Lewy body disease; Lewy body Braak stage 6; amyloid and tau pathology present (low AD change); fresh cerebellar infarct	11y	80
P12 - PD991	68	PD; prostate cancer	M		Levodopa; dopamine agonists; cholinesterase inhibitor; anticoagulant; quetiapine (atypical antipsychotic); antiarrhythmic; laxative; opiate; NSAIDs; sildenafil/viagra; proton pump inhibitor (for gastric acid); steroid; statin; LHRH agonists for prostate cancer; antiandrogen; mucolytic; antibiotic; benzodiazepines; vit B12 suppl; diuretic; SSRI; antibiotics; Metoclopramide (peripheral dopamine antagonist)	Idiopathic PD; tremor; depression (preceeding); overweight; rigidity; dysphagia; hypophonia; pain; urinary urgency; constipation; bradyphrenia (slowness of thought); sleep problem; weight loss; paranoia, hallucinations, psychosis; hypertension; basal cell carcinoma; prostate cancer with metastasis to bone; cellulitis; UTI	Depigmentation of SN; a-syn pathology in the brainstem, limbic system and neocortex; neocortical Lewy body disease; Lewy body Braak stage 6; low AD change; small ischaemic changes; haemorrhage occipital lobe; severe cerebral amyloid angiopathy with capillary involvement	10y	58
P13 - PD1003	71	PD	M		Antimuscarinics (antispasmodics); beta1 R blocker (for hypertension); glutamate antagonist; dopamine agonist; levodopa; dopamine antagonist (peripheral); anticoagulant; ferrous sulphate (for anaemia); proton pump inhibitor (for gastric ulcer); antibiotic; choliesterase inhibitor; SSRI; NSAIDs; cyclopyrrolone (for insomnia); glyceryl trinitrate (for angina)	Idiopathic PD (family history of PD); demetia; sleep problem / REM sleep behaviour disorder; tremor; hypomimia; motor fluctuations, freezing, dyskinesias; peripheral oedema; hypotension; depression; hallucinations; fatigue; anaemia; oesophagitis; hiatus hernia; forgets medications; speech problem; choreo-athetoid movements; memory problems; psychotic phenomena; confusion; poor appetite	Marked depigmentation of SN; neocortical a-syn pathology; Lewy body Braak stage 6; neocortical Lewy body disease; no amyloid pathology; moderate tau pathology	22y	49
P14 - PD1004	87	PD	M		Levodopa; angiotensin R blocker (for hypertension); cholinergic antagonist (for overactive bladder); antimuscarinic; diuretics; calcium channel blocker (for hypertension); NSADs; laxative; SSRI; antiandrogen (DHT inhibitor); antiplatelet; vitD suppl	Idiopathic PD; motor fluctuations, tremor, stiffness, bradykinesia, falls; paraesthesia; dystonia; hypomimia; nocturnal seizure; dysphagia; hypertension; bladder issues; hallucinations; aggression; cognitive impairment, confusion; inflamed sebaceous cyst; poor appetite; syncope; cough, fever	Small vessel disease; moderate atheroma; moderate SN depigmentation; neocortical a-syn pathology; Lewy body Braak stage 6; neocortical Lewy body disease; mild amyloid and tau presence; moderate cerebral amyloid angiopathy	10y	77
P15 - PD1023	79	PD	M		Levodopa; MAO-B inhibitor; dopamine agonist; SSRIs; alpha1 R antagonist (for prostate hyperplasia); antimuscarinic (for bladder); diuretic; steroid; cholinesterase inhibitor; periph opioid antagonist (for diarrhoea); opiate; hyoscine hydrobromide (for vomiting); levomeproxamine (phenothiazine neuroleptic); benzodiazepine	Idiopathic PD; motor fluctuations, falls, tremor, hypomimia, festination; depression; hallucinations; hypotension; syncope; incontinence, nocturia; dysphonia, dysarthria; micrographia; diplopia; dementia, confusion; sleeping problem; memory problem; weight loss; UTIs; aspiration pneumonia	Severe SN depigmentation; a-syn pathology in the brainstem, limbic system and neocortex; neocortical Lewy body disease; Lewy body Braak stage 6; aging related tau astrogliopathy in basal ganglia; possible primary aging associated tauopathy; very mild amyloid pathology; low AD change	14y	65
P16 - PD1049	87	PD; bronchopneumonia	M		Levodopa; angiotensin R blocker (for hypertension); cholinesterase inhibitor; glutamate antagonist; cyclopyrrolone (for insomnia); laxative; dopamine agonists; antimuscarinic (for sickness); benzodiazepine; amitriptyline (trycyclic antidepressant NB acts on Cx43); steroid; dopamine antagonsit peripheral; MAO-B inhibitor/ glutamate release inhibitor/ dopamine+serotonin reuptake inhibitor (safinamide); NMDA R antagonist (memantine)	Idiopathic PD; motor fluctuations, falls, dyskinesia; punding; constipation; postural hypotension; hypertension; sialorrhoea; sleep problem; weight loss; depression; memory problem; fatigue; confusion; hallucinations; delusions; paranoia; aggression; cardiac pacemaker; basal cell carcinoma; osteoarthritis; congestive heart failure; chronic kidney disease	Mild cortical atrophy; moderate SN depigmentation; a-syn pathology in the brainstem, limbic system and neocortex; neocortical Lewy body disease; Lewy body Braak stage 5; perivascular and supependymal aging related tau astrogliopathy in basal ganglia; mild meningeal cerebral amyloid angiopathy; intermediate AD change	20y	67
P17 - PD1059	87	PD	F				Atheroma; moderate SN depigmentation; a-syn pathology in the brainstem, limbic system and neocortex; neocortical Lewy body disease; Lewy body Braak stage 6; no amyloid pathology; primary aging related tauopathy	11y	76
P18 - PD1086	78	PD; Lewy body dementia	M		Levodopa; dopamine agonist; antimuscarinic (antispasmodic); cholinesterase inhibitor; antipsychotic (quetiapine); NMDA+nicotinic antagonist (amantadine); benzodiazepines; phenothiazine; opiates; NSAIDs; laxative; amitriptyline (trycyclic antidepressant NB acts on Cx43)	Idiopathic, potentially atypical PD / progressive supranuclear palsy; early cognitive decline; memory problem; confusion; dementia; constipation; diplopia; anosmia; cerebrovascular disease; dysphonia, dysarthria; dystonia; dysphagia; headaches; dyskinesia; tremor, bradykinesia, rigidity, festination, falls; frontal lobe function insufficiency; hallucinations; sleep problem; UTIs	Widespread microvascular disease; atheroma; mild atrophy of the temporal lobe; enlarged ventricles; SN depigmentation; a-syn pathology in the brainstem, limbic system and neocortex; neocortical Lewy body disease; Lewy body Braak stage 6; amyloid and tau pathology present; low AD change; old cystic infarct	20y	58
P19 - PD1097	65	PD; dementia	F		Levodopa; MAO-B inhibitor; cholinesterase inhibitor; dopamine reuptake inhibitor (modafinil NB upregulates Cx30 GJs?); opioid; vit B suppl's; antibiotic; proton pump inhibitor (for gastric ulcer); cyclopyrrolone (for insomnia); SSRI; NSAID; benzodiazepine; antihistamine, antimuscarinic	Idiopathic PD; cognitive impairment; dementia; depression; dyskinesia; dystonia; dysarthria; dysphagia; anxiety; motor fluctuations, falls; camptocormia; sleep problem; hallucinaitions; memory problem; postural hypotension; meniscal tear, knee surgery; mood swings; confusion; weight loss; UTIs (NB: noted to contribute to confusion); agitation; obesity; small bowel obstruction; hay fever	Marked SN depigmentation; severe dopaminergic neurone loss; frequent astrocytic a-syn pathology; a-syn pathology in the brainstem, limbic system and neocortex; neocortical Lewy body disease; Lewy body Braak stage 6; amyloid and tau pathology present; intermediate AD change; severe cerebral amyloid angiopathy in frontal lobe	20y	45
P20 - PD1113	80	PD	M		Levodopa; dopamine agonists; MAO-B inhibitor; SSRIs; benzodiazepine; opiate	Tremor-dominant PD; motor fluctuations, freezing; positive family history; dementia; hallucinations; depression, suicidal; pain; hypophonia; dyskinesia; fatigue; sleep problem; postural hypotension; memory problem; aggression; confusion; dystonia; hypomimia; constipation; incontinence; sialorrhoea; hypophonia; angina; stroke; osteoarthritis; hypertension	Mild small vessel disease; mild cortical atrophy; severe SN depigmentation; severe dopaminergic neurone loss; a-syn pathology in the brainstem, limbic system and neocortex; neocortical Lewy body disease; Lewy body Braak stage 6; amyloid and tau pathology present; intermediate AD change; mild meningeal cerebral amyloid angiopathy	15y	65

Supplementary Table 3. *Cx43 expression and puncta correlation*. Relationship between Cx43 protein expression and GJ puncta; all samples (control and PD) were included in the analysis. **Text in bold** indicates statistically significant p values ($p<0.05$) where the Spearman's correlation coefficient indicates a positive (direct) correlation. WB – Western blot; SN – substantia nigra; Cx43 – connexin 43; PD – Parkinson's disease.

Cx43 expression and puncta - all samples

Spearman's correlation p-values	Cx43 expression (WB) in midbrain SN	Cx43 expression (WB) in parietal cortex	Cx43 expression (WB) in striatum	Puncta per cell in caudate	Puncta per cell in globus pallidus	Puncta per cell in putamen	Puncta per cell in frontal cortex	Puncta per cell in insular cortex	Puncta per cell in midbrain SN	Puncta per cell in parietal cortex
Cx43 expression (WB) in midbrain SN	N/A	0.055	**0.026**	0.087	0.118	0.243	**0.010**	0.247	**0.002**	**0.007**
Cx43 expression (WB) in parietal cortex	0.055	N/A	**0.000**	**0.036**	**0.002**	**0.008**	**0.000**	**0.021**	**0.003**	**0.005**
Cx43 expression (WB) in striatum	**0.026**	**0.000**	N/A	**0.027**	**0.000**	0.086	**0.000**	0.139	**0.003**	**0.009**
Puncta per cell in caudate	0.087	**0.036**	**0.027**	N/A	**0.000**	**0.000**	0.067	**0.000**	0.052	**0.001**
Puncta per cell in globus pallidus	0.118	**0.002**	**0.000**	**0.000**	N/A	**0.000**	**0.011**	**0.001**	0.065	**0.007**
Puncta per cell in putamen	0.243	**0.008**	0.086	**0.000**	**0.000**	N/A	0.081	**0.001**	**0.003**	0.061
Puncta per cell in frontal cortex	**0.010**	**0.000**	**0.000**	0.067	**0.011**	0.081	N/A	**0.004**	**0.049**	**0.001**
Puncta per cell in insular cortex	0.247	**0.021**	0.139	**0.000**	**0.001**	**0.001**	**0.004**	N/A	0.294	**0.000**
Puncta per cell in midbrain SN	**0.002**	**0.003**	**0.003**	0.052	0.065	**0.003**	**0.049**	0.294	N/A	**0.006**
Puncta per cell in parietal cortex	**0.007**	**0.005**	**0.009**	**0.001**	**0.007**	0.061	**0.001**	**0.000**	**0.006**	N/A

Spearman's correlation coefficient	Cx43 expression (WB) in midbrain SN	Cx43 expression (WB) in parietal cortex	Cx43 expression (WB) in striatum	Puncta per cell in caudate	Puncta per cell in globus pallidus	Puncta per cell in putamen	Puncta per cell in frontal cortex	Puncta per cell in insular cortex	Puncta per cell in midbrain SN	Puncta per cell in parietal cortex
Cx43 expression (WB) in midbrain SN	1.000	0.314	0.362	0.329	0.291	0.194	0.410	0.195	0.495	0.439
Cx43 expression (WB) in parietal cortex	0.314	1.000	0.698	0.391	0.545	0.417	0.623	0.372	0.454	0.441
Cx43 expression (WB) in striatum	0.362	0.698	1.000	0.409	0.593	0.278	0.586	0.245	0.452	0.412
Puncta per cell in caudate	0.329	0.391	0.409	1.000	0.679	0.731	0.345	0.688	0.364	0.572
Puncta per cell in globus pallidus	0.291	0.545	0.593	0.679	1.000	0.646	0.448	0.553	0.335	0.472
Puncta per cell in putamen	0.194	0.417	0.278	0.731	0.646	1.000	0.283	0.526	0.459	0.306
Puncta per cell in frontal cortex	0.410	0.623	0.586	0.345	0.448	0.283	1.000	0.458	0.313	0.520
Puncta per cell in insular cortex	0.195	0.372	0.245	0.688	0.553	0.526	0.458	1.000	0.175	0.661
Puncta per cell in midbrain SN	0.495	0.454	0.452	0.364	0.335	0.459	0.313	0.175	1.000	0.430
Puncta per cell in parietal cortex	0.439	0.441	0.412	0.572	0.472	0.306	0.520	0.661	0.430	1.000

Supplementary Table 4. *Cx43 correlation with PD pathology.* Relationships between Cx43 protein expression and GJ puncta in various brain regions; samples from the PD cohort were included in the analysis. **Text in bold** indicates statistically significant p values ($p<0.05$) where the Spearman's correlation coefficient indicates a positive (direct) correlation, and ***italicised text in bold*** indicates statistically significant p values where the Spearman's correlation coefficient indicates a negative (inverse) correlation. WB – Western blot; Mid SN – midbrain substantia nigra; PD – Parkinson's disease; CAA – cerebral amyloid angiopathy; LB – Lewy body; Cx43 – connexin 43.

Cx43 and PD pathology - PD cohort

Spearman's correlation p-values

	Amyloid Braak	LB / a-syn Braak	PD duration (years)	Mid SN depigmentation	Tau Braak	CAA
Amyloid Braak	N/A	0.709	0.758	0.909	**0.041**	0.538
LB / a-syn Braak	0.709	N/A	0.138	0.039	0.829	**0.041**
PD duration (years)	0.758	0.138	N/A	**0.047**	0.302	0.501
Mid SN depigmentation	0.909	**0.039**	**0.047**	N/A	0.465	0.398
Tau Braak	**0.041**	0.829	0.302	0.465	N/A	0.213
CAA	0.538	**0.041**	0.501	0.398	0.213	N/A
Cx43 expression (WB) in midbrain SN	0.524	0.937	0.739	0.863	0.643	0.876
Cx43 expression (WB) in parietal cortex	0.710	0.874	0.209	0.916	0.258	0.935
Cx43 expression (WB) in striatum	0.914	0.524	0.760	0.267	***0.024***	0.905
Puncta per cell in caudate	0.713	0.259	0.850	0.337	0.833	0.472
Puncta per cell in globus pallidus	0.305	***0.040***	0.547	0.470	0.165	0.919
Puncta per cell in putamen	0.343	0.524	0.937	0.854	0.177	0.655
Puncta per cell in frontal cortex	0.703	**0.021**	0.262	0.247	0.681	0.723
Puncta per cell in insular cortex	0.428	0.379	0.283	0.594	0.102	0.884
Puncta per cell in midbrain SN	0.275	0.634	0.869	0.341	0.281	0.943
Puncta per cell in parietal cortex	0.974	0.812	0.536	***0.041***	0.502	0.731

Spearman's correlation coefficient

	Amyloid Braak	LB / a-syn Braak	PD duration (years)	Mid SN depigmentation	Tau Braak	CAA
Amyloid Braak	1.000	-0.089	0.074	0.027	0.461	0.146
LB / a-syn Braak	-0.089	1.000	0.343	0.464	-0.051	0.460
PD duration (years)	0.074	0.343	1.000	0.450	0.243	0.160
Mid SN depigmentation	0.027	0.464	0.450	1.000	-0.173	0.200
Tau Braak	0.461	-0.051	0.243	-0.173	1.000	-0.291
CAA	0.146	0.460	0.160	0.200	-0.291	1.000
Cx43 expression (WB) in midbrain SN	0.151	0.019	0.080	0.041	-0.110	0.037
Cx43 expression (WB) in parietal cortex	0.089	-0.038	0.293	-0.025	-0.266	0.019
Cx43 expression (WB) in striatum	0.026	0.151	0.073	0.261	-0.503	0.028
Puncta per cell in caudate	0.108	-0.324	-0.056	-0.277	-0.062	-0.209
Puncta per cell in globus pallidus	-0.284	-0.535	-0.169	-0.202	-0.378	-0.029
Puncta per cell in putamen	-0.224	-0.151	-0.019	0.044	-0.315	0.106
Puncta per cell in frontal cortex	-0.091	0.511	0.263	0.271	-0.098	0.084
Puncta per cell in insular cortex	-0.188	-0.208	-0.252	-0.127	-0.376	0.035
Puncta per cell in midbrain SN	-0.257	-0.114	-0.039	-0.225	-0.253	-0.017
Puncta per cell in parietal cortex	0.008	0.057	-0.147	-0.461	-0.159	0.082

Spearman's correlation p-values - Parietal cortex local analysis		
	A-syn inclusions in parietal cortex	Tau inclusions in parietal cortex
Puncta per cell in parietal cortex	0.137	0.885

Spearman's correlation coefficient - Parietal cortex local analysis		
	A-syn inclusions in parietal cortex	Tau inclusions in parietal cortex
Puncta per cell in parietal cortex	-0.273	-0.027

Spearman's correlation p-values - Frontal cortex local analysis			
	A-syn inclusions in frontal cortex	Tau inclusions in frontal cortex	Amyloid inclusions in frontal cortex
Puncta per cell in frontal cortex	0.348	0.634	0.789

Spearman's correlation coefficient - Frontal cortex local analysis			
	A-syn inclusions in frontal cortex	Tau inclusions in frontal cortex	Amyloid inclusions in frontal cortex
Puncta per cell in frontal cortex	-0.174	-0.089	0.050

Supplementary Table 5. *Cx43 expression with other markers*. Relationships between Cx43 expression and several other markers of astrocytes (Aldh1L1, GFAP) plus a microglial marker Iba1; samples from the control and PD cohorts were analysed independently. **Text in bold** indicates statistically significant p values where the Spearman's correlation coefficient indicates a positive (direct) correlation, and ***italicised text in bold*** indicates statistically significant p values where the Spearman's correlation coefficient indicates a negative (inverse) correlation. WB – Western blot; SN – substantia nigra; PD – Parkinson's disease; Cx43 – connexin 43; GFAP – glial fibrillary acidic protein.

Cx43 expression and other markers

Spearman's correlation p-values - Aldh1L1 control			
	ALdh1L1 expression (WB) in midbrain SN	Aldh1L1 expression (WB) in parietal cortex	Aldh1l1 expression (WB) in striatum
ALdh1L1 expression (WB) in midbrain SN	N/A	**0.003**	**0.008**
Aldh1L1 expression (WB) in parietal cortex	**0.003**	N/A	**0.002**
Aldh1l1 expression (WB) in striatum	**0.008**	**0.002**	N/A
Cx43 expression (WB) in midbrain SN	0.699	0.376	0.531
Cx43 expression (WB) in parietal cortex	0.099	**0.024**	**0.001**
Cx43 expression (WB) in striatum	0.101	**0.006**	**0.005**

Spearman's correlation coefficient - Aldh1L1 control			
	ALdh1L1 expression (WB) in midbrain SN	Aldh1L1 expression (WB) in parietal cortex	Aldh1l1 expression (WB) in striatum
ALdh1L1 expression (WB) in midbrain SN	1.000	0.653	0.604
Aldh1L1 expression (WB) in parietal cortex	0.653	1.000	0.660
Aldh1l1 expression (WB) in striatum	0.604	0.660	1.000
Cx43 expression (WB) in midbrain SN	0.098	-0.222	-0.158
Cx43 expression (WB) in parietal cortex	0.401	0.504	0.669
Cx43 expression (WB) in striatum	0.399	0.591	0.605

Spearman's correlation p-values - Aldh1L1 PD			
	ALdh1L1 expression (WB) in midbrain SN	Aldh1L1 expression (WB) in parietal cortex	Aldh1l1 expression (WB) in striatum
ALdh1L1 expression (WB) in midbrain SN	N/A	**0.000**	**0.002**
Aldh1L1 expression (WB) in parietal cortex	**0.000**	N/A	**0.000**
Aldh1l1 expression (WB) in striatum	**0.002**	**0.000**	N/A
Cx43 expression (WB) in midbrain SN	0.130	0.186	0.137
Cx43 expression (WB) in parietal cortex	0.198	0.895	0.885
Cx43 expression (WB) in striatum	0.227	0.613	0.398

Spearman's correlation coefficient - Aldh1L1 PD			
	ALdh1L1 expression (WB) in midbrain SN	Aldh1L1 expression (WB) in parietal cortex	Aldh1l1 expression (WB) in striatum
ALdh1L1 expression (WB) in midbrain SN	1.000	0.723	0.660
Aldh1L1 expression (WB) in parietal cortex	0.723	1.000	0.750
Aldh1l1 expression (WB) in striatum	0.660	0.750	1.000
Cx43 expression (WB) in midbrain SN	-0.350	-0.308	-0.344
Cx43 expression (WB) in parietal cortex	-0.301	0.032	-0.035
Cx43 expression (WB) in striatum	-0.283	-0.120	-0.200

Spearman's correlation p-values - GFAP control			
	GFAP expression (WB) in midbrain SN	GFAP expression (WB) in parietal cortex	GFAP expression (WB) in striatum
Cx43 expression (WB) in midbrain SN	0.188	0.349	0.390
Cx43 expression (WB) in parietal cortex	***0.016***	0.316	0.112
Cx43 expression (WB) in striatum	***0.035***	0.286	0.319
GFAP expression (WB) in midbrain SN	N/A	**0.030**	**0.014**
GFAP expression (WB) in parietal cortex	**0.030**	N/A	**0.001**
GFAP expression (WB) in striatum	**0.014**	**0.001**	N/A

Spearman's correlation coefficient - GFAP control			
	GFAP expression (WB) in midbrain SN	GFAP expression (WB) in parietal cortex	GFAP expression (WB) in striatum
Cx43 expression (WB) in midbrain SN	0.325	0.234	0.216
Cx43 expression (WB) in parietal cortex	-0.560	-0.236	-0.367
Cx43 expression (WB) in striatum	-0.498	-0.251	-0.235
GFAP expression (WB) in midbrain SN	1.000	0.513	0.567
GFAP expression (WB) in parietal cortex	0.513	1.000	0.662
GFAP expression (WB) in striatum	0.567	0.662	1.000

Spearman's correlation p-values - GFAP PD			
	GFAP expression (WB) in midbrain SN	GFAP expression (WB) in parietal cortex	GFAP expression (WB) in striatum
Cx43 expression (WB) in midbrain SN	0.116	0.645	**0.048**
Cx43 expression (WB) in parietal cortex	0.870	0.319	0.945
Cx43 expression (WB) in striatum	0.490	0.772	0.454
GFAP expression (WB) in midbrain SN	N/A	0.490	0.230
GFAP expression (WB) in parietal cortex	0.490	N/A	0.600
GFAP expression (WB) in striatum	0.230	0.600	N/A

Spearman's correlation coefficient - GFAP PD			
	GFAP expression (WB) in midbrain SN	GFAP expression (WB) in parietal cortex	GFAP expression (WB) in striatum
Cx43 expression (WB) in midbrain SN	0.362	-0.110	0.448
Cx43 expression (WB) in parietal cortex	-0.039	0.235	-0.017
Cx43 expression (WB) in striatum	-0.164	-0.069	0.177
GFAP expression (WB) in midbrain SN	1.000	0.164	0.281
GFAP expression (WB) in parietal cortex	0.164	1.000	0.125
GFAP expression (WB) in striatum	0.281	0.125	1.000

Spearman's correlation p-values - Iba1 control	
	Iba1 expression in parietal cortex
Cx43 expression (WB) in midbrain SN	0.376
Cx43 expression (WB) in parietal cortex	0.498
Cx43 expression (WB) in striatum	0.955
Puncta per cell in caudate	0.191
Puncta per cell in globus pallidus	0.087
Puncta per cell in putamen	0.185
Puncta per cell in frontal cortex	0.796
Puncta per cell in insular cortex	0.075
Puncta per cell in midbrain SN	0.569
Puncta per cell in parietal cortex	0.351

Spearman's correlation coefficient - Iba1 control	
	Iba1 expression in parietal cortex
Cx43 expression (WB) in midbrain SN	0.222
Cx43 expression (WB) in parietal cortex	0.161
Cx43 expression (WB) in striatum	0.014
Puncta per cell in caudate	0.357
Puncta per cell in globus pallidus	0.441
Puncta per cell in putamen	0.318
Puncta per cell in frontal cortex	-0.062
Puncta per cell in insular cortex	0.430
Puncta per cell in midbrain SN	0.135
Puncta per cell in parietal cortex	0.226

Spearman's correlation p-values - Iba1 PD	
	Iba1 expression in parietal cortex
Cx43 expression (WB) in midbrain SN	0.141
Cx43 expression (WB) in parietal cortex	0.424
Cx43 expression (WB) in striatum	0.446
Puncta per cell in caudate	***0.009***
Puncta per cell in globus pallidus	0.383
Puncta per cell in putamen	0.821
Puncta per cell in frontal cortex	0.627
Puncta per cell in insular cortex	0.450
Puncta per cell in midbrain SN	0.502
Puncta per cell in parietal cortex	0.271

Spearman's correlation coefficient - Iba1 PD	
	Iba1 expression in parietal cortex
Cx43 expression (WB) in midbrain SN	-0.341
Cx43 expression (WB) in parietal cortex	-0.189
Cx43 expression (WB) in striatum	-0.180
Puncta per cell in caudate	-0.670
Puncta per cell in globus pallidus	-0.243
Puncta per cell in putamen	-0.054
Puncta per cell in frontal cortex	-0.116
Puncta per cell in insular cortex	-0.179
Puncta per cell in midbrain SN	-0.159
Puncta per cell in parietal cortex	-0.259

Supplementary Table 6. *Iba1 expression and PD hallmarks correlation.* Relationship between Iba1 microglial marker protein expression and a number of PD hallmarks; samples from the PD cohort were included in the analysis. PD – Parkinson's disease; WB – Western blot; LB – Lewy body; a-syn – alpha-synuclein; Mid SN – midbrain substantia nigra; CAA – cerebral amyloid angiopathy.

Iba1 expression and PD hallmarks - PD cohort

Spearman's correlation p-values

	Age of death	Amyloid Braak	LB / a-syn Braak	PD duration (years)	Mid SN depigmentation	Tau Braak	CAA
Iba1 expression (WB) in parietal cortex	0.483	0.245	0.068	0.613	0.079	0.368	0.611

Spearman's correlation coefficient

	Age of death	Amyloid Braak	LB / a-syn Braak	PD duration (years)	Mid SN depigmentation	Tau Braak	CAA
Iba1 expression (WB) in parietal cortex	-0.167	-0.272	0.416	0.121	0.402	-0.213	0.121

Supplementary Table 7. *Cx43 expression correlation with age and PD duration.* Relationship between Cx43 protein expression as well as GJ puncta and the age of death plus the recorded duration of disease; samples from the PD cohort were included. PD – Parkinson's disease; Cx43 – connexin 43; WB – Western blot; SN – substantia nigra.

Cx43 expression, age, and PD duration - PD cohort

Spearman's correlation p-values										
	Cx43 expression (WB) in midbrain SN	Cx43 expression (WB) in parietal cortex	Cx43 expression (WB) in striatum	Puncta per cell in caudate	Puncta per cell in globus pallidus	Puncta per cell in putamen	Puncta per cell in frontal cortex	Puncta per cell in insular cortex	Puncta per cell in midbrain SN	Puncta per cell in parietal cortex
Age of death	0.769	0.208	0.481	0.332	0.289	0.202	0.361	0.653	0.937	0.731
PD duration	0.739	0.209	0.760	0.850	0.547	0.937	0.262	0.283	0.869	0.536

Spearman's correlation coefficient										
	Cx43 expression (WB) in midbrain SN	Cx43 expression (WB) in parietal cortex	Cx43 expression (WB) in striatum	Puncta per cell in caudate	Puncta per cell in globus pallidus	Puncta per cell in putamen	Puncta per cell in frontal cortex	Puncta per cell in insular cortex	Puncta per cell in midbrain SN	Puncta per cell in parietal cortex
Age of death	0.070	0.294	0.167	0.280	0.293	0.298	0.216	0.107	-0.019	0.082
PD duration	0.080	0.293	0.073	-0.056	-0.169	-0.019	0.263	-0.252	-0.039	-0.147

Supplementary Bibliography:

1. Dijkstra AA, Ingrassia A, de Menezes RX, et al (2015) Evidence for Immune Response, Axonal Dysfunction and Reduced Endocytosis in the Substantia Nigra in Early Stage Parkinson's Disease. PLoS One 10:e0128651
2. Smajic S, Prada-Medina CA, Landoulsi Z, et al (2022) Single-cell sequencing of human midbrain reveals glial activation and a Parkinson-specific neuronal state. Brain 145:964–978. https://doi.org/10.1093/brain/awab446
3. Xu J, Farsad HL, Hou Y, et al (2023) Human striatal glia differentially contribute to AD- and PD-specific neurodegeneration. Nat Aging. https://doi.org/10.1038/s43587-023-00363-8
4. Martirosyan A, Ansari R, Pestana F, et al (2024) Unravelling cell type-specific responses to Parkinson's Disease at single cell resolution. Mol Neurodegener 19:7
